# Interventions for treatment of COVID-19: Second edition of a living systematic review with meta-analyses and trial sequential analyses (The LIVING Project)

**DOI:** 10.1371/journal.pone.0248132

**Published:** 2021-03-11

**Authors:** Sophie Juul, Emil Eik Nielsen, Joshua Feinberg, Faiza Siddiqui, Caroline Kamp Jørgensen, Emily Barot, Johan Holgersson, Niklas Nielsen, Peter Bentzer, Areti Angeliki Veroniki, Lehana Thabane, Fanlong Bu, Sarah Klingenberg, Christian Gluud, Janus Christian Jakobsen

**Affiliations:** 1 Copenhagen Trial Unit–Centre for Clinical Intervention Research, Rigshospitalet, Copenhagen University Hospital, Copenhagen, Denmark; 2 Department of Internal Medicine–Cardiology Section, Holbæk Hospital, Holbæk, Denmark; 3 Department of Clinical Sciences Lund, Anesthesia & Intensive Care, Helsingborg Hospital, Lund University, Lund, Sweden; 4 Department of Primary Education, School of Education, University of Ioannina, Ioannina, Greece; 5 Knowledge Translation Program, Li Ka Shing Knowledge Institute, St. Michael’s Hospital, Toronto, Ontario, Canada; 6 Department of Health Research Methods, Evidence, and Impact, McMaster University, Hamilton, ON, Canada; 7 Centre for Evidence-based Chinese Medicine, Beijing University of Chinese Medicine, Beijing, China; 8 Faculty of Health Sciences, University of Southern Denmark, Odense, Denmark; Humanitas University, ITALY

## Abstract

**Background:**

COVID-19 is a rapidly spreading disease that has caused extensive burden to individuals, families, countries, and the world. Effective treatments of COVID-19 are urgently needed. This is the second edition of a living systematic review of randomized clinical trials assessing the effects of all treatment interventions for participants in all age groups with COVID-19.

**Methods and findings:**

We planned to conduct aggregate data meta-analyses, trial sequential analyses, network meta-analysis, and individual patient data meta-analyses. Our systematic review was based on PRISMA and Cochrane guidelines, and our eight-step procedure for better validation of clinical significance of meta-analysis results. We performed both fixed-effect and random-effects meta-analyses. Primary outcomes were all-cause mortality and serious adverse events. Secondary outcomes were admission to intensive care, mechanical ventilation, renal replacement therapy, quality of life, and non-serious adverse events. According to the number of outcome comparisons, we adjusted our threshold for significance to *p* = 0.033. We used GRADE to assess the certainty of evidence. We searched relevant databases and websites for published and unpublished trials until November 2, 2020. Two reviewers independently extracted data and assessed trial methodology. We included 82 randomized clinical trials enrolling a total of 40,249 participants. 81 out of 82 trials were at overall high risk of bias. Meta-analyses showed no evidence of a difference between corticosteroids versus control on all-cause mortality (risk ratio [RR] 0.89; 95% confidence interval [CI] 0.79 to 1.00; *p* = 0.05; I^2^ = 23.1%; eight trials; very low certainty), on serious adverse events (RR 0.89; 95% CI 0.80 to 0.99; *p* = 0.04; I^2^ = 39.1%; eight trials; very low certainty), and on mechanical ventilation (RR 0.86; 95% CI 0.55 to 1.33; *p* = 0.49; I^2^ = 55.3%; two trials; very low certainty). The fixed-effect meta-analyses showed indications of beneficial effects. Trial sequential analyses showed that the required information size for all three analyses was not reached. Meta-analysis (RR 0.93; 95% CI 0.82 to 1.07; *p* = 0.31; I^2^ = 0%; four trials; moderate certainty) and trial sequential analysis (boundary for futility crossed) showed that we could reject that remdesivir versus control reduced the risk of death by 20%. Meta-analysis (RR 0.82; 95% CI 0.68 to 1.00; *p* = 0.05; I^2^ = 38.9%; four trials; very low certainty) and trial sequential analysis (required information size not reached) showed no evidence of difference between remdesivir versus control on serious adverse events. Fixed-effect meta-analysis showed indications of a beneficial effect of remdesivir on serious adverse events. Meta-analysis (RR 0.40; 95% CI 0.19 to 0.87; *p* = 0.02; I^2^ = 0%; two trials; very low certainty) showed evidence of a beneficial effect of intravenous immunoglobulin versus control on all-cause mortality, but trial sequential analysis (required information size not reached) showed that the result was severely underpowered to confirm or reject realistic intervention effects. Meta-analysis (RR 0.63; 95% CI 0.35 to 1.14; *p* = 0.12; I^2^ = 77.4%; five trials; very low certainty) and trial sequential analysis (required information size not reached) showed no evidence of a difference between tocilizumab versus control on serious adverse events. Fixed-effect meta-analysis showed indications of a beneficial effect of tocilizumab on serious adverse events. Meta-analysis (RR 0.70; 95% CI 0.51 to 0.96; *p* = 0.02; I^2^ = 0%; three trials; very low certainty) showed evidence of a beneficial effect of tocilizumab versus control on mechanical ventilation, but trial sequential analysis (required information size not reached) showed that the result was severely underpowered to confirm of reject realistic intervention effects. Meta-analysis (RR 0.32; 95% CI 0.15 to 0.69; *p* < 0.00; I^2^ = 0%; two trials; very low certainty) showed evidence of a beneficial effect of bromhexine versus standard care on non-serious adverse events, but trial sequential analysis (required information size not reached) showed that the result was severely underpowered to confirm or reject realistic intervention effects. Meta-analyses and trial sequential analyses (boundary for futility crossed) showed that we could reject that hydroxychloroquine versus control reduced the risk of death and serious adverse events by 20%. Meta-analyses and trial sequential analyses (boundary for futility crossed) showed that we could reject that lopinavir-ritonavir versus control reduced the risk of death, serious adverse events, and mechanical ventilation by 20%. All remaining outcome comparisons showed that we did not have enough information to confirm or reject realistic intervention effects. Nine single trials showed statistically significant results on our outcomes, but were underpowered to confirm or reject realistic intervention effects. Due to lack of data, it was not relevant to perform network meta-analysis or possible to perform individual patient data meta-analyses.

**Conclusions:**

No evidence-based treatment for COVID-19 currently exists. Very low certainty evidence indicates that corticosteroids might reduce the risk of death, serious adverse events, and mechanical ventilation; that remdesivir might reduce the risk of serious adverse events; that intravenous immunoglobin might reduce the risk of death and serious adverse events; that tocilizumab might reduce the risk of serious adverse events and mechanical ventilation; and that bromhexine might reduce the risk of non-serious adverse events. More trials with low risks of bias and random errors are urgently needed. This review will continuously inform best practice in treatment and clinical research of COVID-19.

**Systematic review registration:**

PROSPERO CRD42020178787.

## Introduction

In December 2019, the emergence of a novel coronavirus, the severe acute respiratory syndrome coronavirus 2 (SARS-CoV-2), caused a rapid international outbreak of the respiratory illness COVID-19 [1]. Since the initial outbreak in China, SARS-CoV-2 has spread globally and COVID-19 is currently labeled a public health emergency of global concern by the World Health Organization [2]. The full clinical spectrum of COVID-19 ranges from asymptomatic infection to mild, self-limiting respiratory tract illness to severe progressive pneumonia, multiorgan failure, and death [3]. Critically ill patients might die from massive alveolar damage and progressive respiratory failure [4–6].

Many randomized clinical trials assessing the effects of different potential treatments for COVID-19 are currently underway. However, it is rare that a single trial can sufficiently assess the effects of any intervention. Therefore, there is an urgent need to continuously surveil the emerging evidence and present aggregate data so that effective treatments, if such exist, are rapidly implemented in clinical practice [7].

The present living systematic review with aggregate meta-analyses and trial sequential analyses aims to continuously inform evidence-based guideline recommendations for the treatment of COVID-19, taking risks of systematic errors (‘bias’), risks of random errors (‘play of chance’), and certainty of the findings into consideration [8].

## Methods

We report this systematic review based on the Preferred Reporting Items for Systematic Reviews and Meta-Analysis (PRISMA) guidelines (**S1 Text**) [9, 10]. The updated methodology used in this living systematic review is according to the Cochrane Handbook of Systematic Reviews of Interventions [11] and described in our protocol [8], which was registered in the PROSPERO database (ID: CRD42020178787) prior to the systematic literature search.

### Search strategy and selection criteria

#### Electronic searches

An information specialist searched the Cochrane Central Register of Controlled Trials (CENTRAL) in The Cochrane Library, Medical Literature Analysis and Retrieval System Online (MEDLINE Ovid), Excerpta Medica database (Embase Ovid), Latin American and Caribbean Health Sciences Literature (LILACS; Bireme), Science Citation Index Expanded (SCI-EXPANDED; Web of Science), Conference Proceedings Citation Index–Science (CPCI-S; Web of Science), BIOSIS (Web of Science), CINAHL (EBSCO host), Chinese Biomedical Literature Database (CBM), China Network Knowledge Information (CNKI), Chinese Science Journal Database (VIP), and Wanfang Database to identify relevant trials. We searched all databases from their inception and until November 2, 2020. Trials were included irrespective of language, publication status, publication year, and publication type. For the detailed search strategies for all electronic searches, see **S2 Text.**

#### Searching other resources

The reference lists of relevant trial publications were checked for any unidentified randomized clinical trials. To identify unpublished trials, we searched clinical trial registries (e.g. clinicaltrials.gov, clinicaltrialregister.eu, who.int/ictrp, chictr.org.cn) of Europe, USA, and China, and websites of pharmaceutical companies and of U.S. Food and Drug Administration (FDA) and European Medicines Agency (EMA). We also searched the COVID-19 Study Registry [12] and the real-time dashboard of randomized trials [13].

We included unpublished and grey literature trials and assessed relevant retraction statements and errata for included trials. We also searched preprint servers (bioRxiv, medRxiv) for unpublished trials. We contacted all corresponding authors to obtain individual patient data.

### Living systematic review

In this living systematic review, two independent investigators receive a weekly updated literature search file, and continuously include relevant newly published or unpublished trials. The relevant meta-analyses, trial sequential analyses, and network meta-analysis will be continuously updated, and if new evidence is available (judged by the author group), the results will be submitted for publication. Every month, the author group will discuss whether searching once a week is necessary. For a detailed overview of the living systematic review work flow, see our protocol [8]. As this is a living systematic review analyzing results of randomized clinical trials, no ethical approval is required.

### Data extraction

Two authors (EEN and JF) independently screened relevant trials. Seven authors in pairs (SJ, EEN, JF, FS, CKJ, EB, JH) independently extracted data using a standardized data extraction sheet. Any discrepancies were resolved through discussion, or if required, through discussion with a third author (JCJ). We contacted corresponding authors if relevant data were unclear or missing.

### Risk of bias assessment

Risk of bias was assessed with the Cochrane Risk of Bias tool–version 2 (RoB 2) [11, 14]. Seven authors in pairs (SJ, EEN, JF, FS, CKJ, EB, JH) independently assessed risk of bias. Any discrepancies were resolved through discussion or, if required, through discussion with a third author (JCJ). Bias was assessed with the following domains: bias arising from the randomization process, bias due to deviations from the intended interventions, bias due to missing outcome data, bias in measurement of outcomes, and bias arising from selective reporting of results [11, 14]. We contacted corresponding authors of trials with unclear or missing data.

### Outcomes and subgroup analyses

Primary and secondary outcomes were predefined in our protocol [8]. Primary outcomes were all-cause mortality and serious adverse events (as defined by the ICH-GCP guidelines) [8, 15]. Secondary outcomes were admission to intensive care (as defined by trialists), mechanical ventilation (as defined by trialists), renal replacement therapy (as defined by trialists), quality of life, and non-serious adverse events. We classified non-serious adverse events as any adverse event not assessed as serious according to the ICH-GCP definition.

We chose to add time to clinical improvement as a post hoc outcome. We planned several subgroup analyses, which are described in detail in our protocol [8]. For all outcomes, we used the trial results reported at maximum follow-up.

### Assessment of statistical and clinical significance

We performed our aggregate data meta-analyses according to Cochrane [11], Keus et al. [16], and the eight-step assessment by Jakobsen et al. [17] for better validation of meta-analytic results in systematic reviews. Review Manager version 5.4 [18] and Stata 16 (StataCorp LLC, College Station, TX, USA) [19] were used for all statistical analyses. We used risk ratios (RR) for dichotomous outcomes. We assessed a total of two primary outcomes per comparison, and we therefore adjusted our thresholds for significance [17] and considered a *p*-value of 0.033 or less as the threshold for statistical significance [8, 17]. Because we primarily considered results of secondary outcomes as hypothesis generating, we did not adjust the *p*-value for secondary outcomes. We conducted both random-effects (DerSimonian-Laird) and fixed-effect (Mantel-Haenszel) meta-analyses for all analyses and chose the most conservative result as our primary result [11, 17, 20, 21]. We used trial sequential analysis to control for random errors [22–30]. Trial sequential analysis estimates the diversity-adjusted required information size (DARIS), which is the number of participants needed in a meta-analysis to detect or reject a certain intervention effect. Statistical heterogeneity was quantified by calculating heterogeneity (I2) for traditional meta-analyses and diversity (D2) for trial sequential analysis. We used Grading Recommendations Assessment Development Evaluation (GRADE) to assess the certainty of evidence. We downgraded imprecision in GRADE by two levels if the accrued number of participants were below 50% of the DARIS, and one level if between 50% and 100% of DARIS [17]. We did not downgrade if benefit, harm, futility or DARIS were reached. We used Fisher’s exact test to calculate *p*-values for all single trial results.

## Results

### Study characteristics

**Fig 1:**
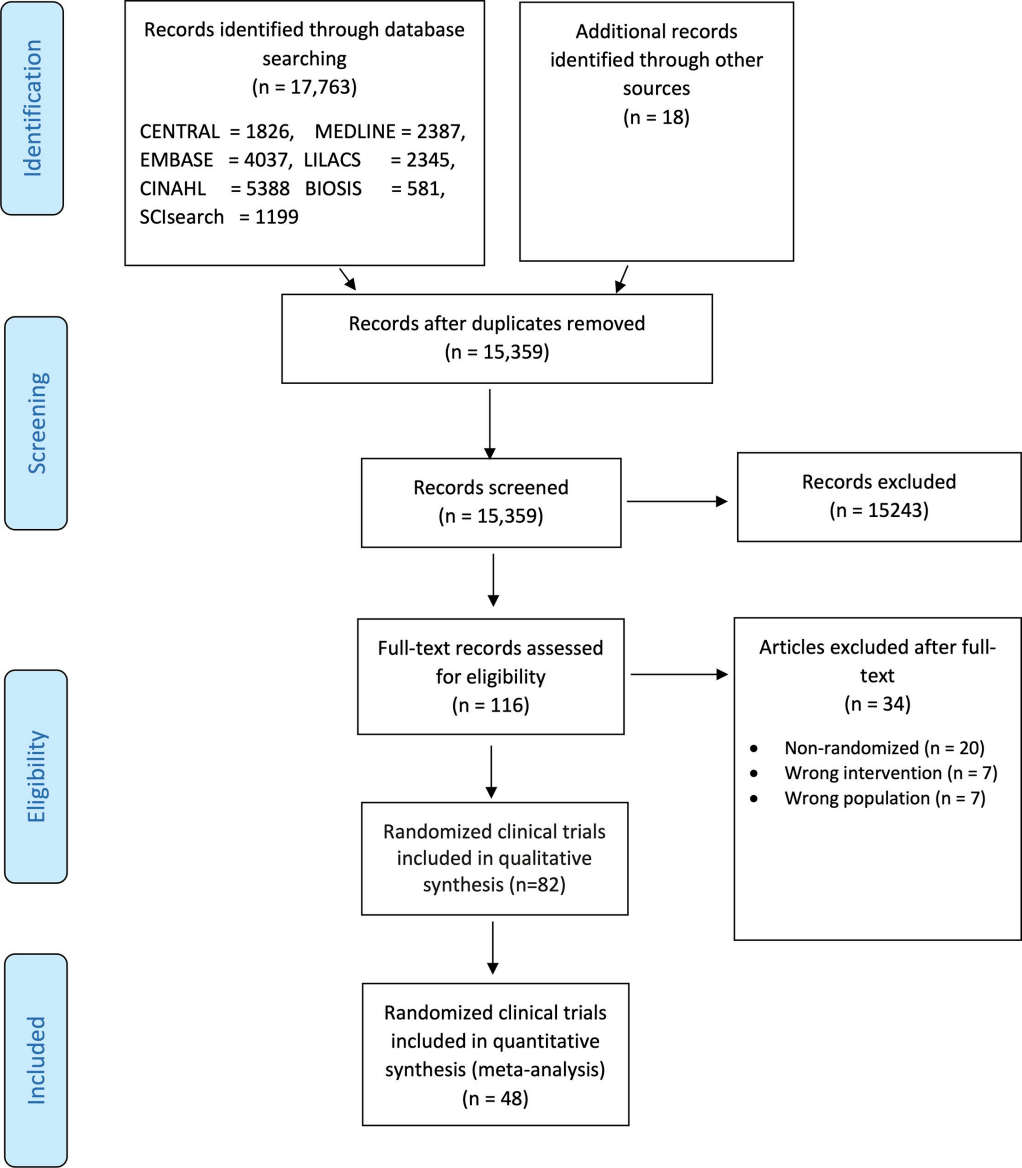
PRISMA flow diagram.

On November 2, 2020 our literature searches identified 15,359 records after duplicates were removed. We included a total of 82 clinical trials randomizing 40,249 participants (**Fig 1**) [31–113]. We identified several trials including participants *suspected* of COVID-19 [114, 115]. None of these trials reported separate data on COVID-19 positive participants compared to the remaining participants. We included trials if approximately 50% or more participants had a confirmed COVID-19 diagnosis. We wrote to all authors requesting separate data on COVID-19 confirmed participants, but we have received no responses yet. For at detailed overview of excluded trials, see **S1 Table.**

Characteristics of included trials and the trial results can be found in **S2 Table**. Most trials were at high risk of bias (**S3 Table**).

The identified trials compared the following interventions: 10 trials compared corticosteroids versus standard care [51, 55, 86, 88, 95–98] or placebo [67, 87]; four trials compared remdesivir versus standard care [85, 109] or placebo [42, 64]; 13 trials compared hydroxychloroquine versus standard care [33, 34, 41, 47, 53, 54, 57, 58, 104, 109], or placebo [52, 107]; five trials compared lopinavir-ritonavir versus standard care [3, 39, 105, 109] or a co-intervention alone [44]; two trials compared interferon beta-1a versus standard care [35, 109]; four trials compared convalescent plasma versus standard care [38, 50, 77, 90]; three trials compared azithromycin versus standard care [82] or co-interventions with standard care [53] or without standard care [81]; three trials compared colchicine versus standard care [48], placebo plus standard care [91], or placebo plus a co-intervention [106]; two trials compared immunoglobulin versus standard care [56] or placebo [94]; six trials compared tocilizumab versus standard care [92, 110–112], placebo with standard care [89] or favipiravir alone as co-intervention [113];two trials compared bromhexine versus standard care [93, 103]; and three trials compared favipiravir versus standard care [40, 66] or a co-intervention alone [113].

The remaining trial comparisons included: favipiravir versus umifenovir [32]; umifenovir versus lopinavir-ritonavir [39]; umifenovir versus standard care [39]; novaferon versus novaferon plus lopinavir-ritonavir [44]; novaferon plus lopinavir-ritonavir versus lopinavir-ritonavir [44]; novaferon versus lopinavir-ritonavir [44]; alpha lipotic acid versus placebo [45]; baloxavir marboxil versus favipiravir [40]; baloxavir marboxil versus standard care [40]; triple combination of interferon beta-1b plus lopinavir-ritonavir plus ribavirin versus lopinavir-ritonavir [37]; remdesivir for 5 days versus remdesivir for 10 days [36]; high-flow nasal oxygenation versus standard bag-valve oxygenation [43]; hydroxychloroquine versus chloroquine [47]; chloroquine versus standard care [47]; high dosage chloroquine diphosphate versus low dosage chloroquine diphosphate [49]; hydroxychloroquine plus azithromycin versus standard care [53]; triple combination of darunavir plus cobicistat plus interferon alpha-2b versus interferon alpha-2b [60]; lopinavir-ritonavir plus interferon alpha versus ribavirin plus interferon alpha [60]; ribavirin plus lopinavir-ritonavir plus interferon alpha versus ribavirin plus interferon alpha [60]; ribavirin plus lopinavir-ritonavir plus interferon alpha versus lopinavir-ritonavir plus interferon alpha [60]; lincomycin versus azithromycin [61]; 99-mTc-methyl diphosphonate (99mTc-MDP) injection versus standard care [62]; interferon alpha-2b plus interferon gamma versus interferon alpha-2b [63]; telmisartan versus standard care [65]; avifavir 1800/800 versus avifavir 1600/600 [66]; dexamethasone plus aprepitant versus dexamethasone [68]; anti-C5a antibody versus standard care [69]; azvudine versus standard care [72]; human plasma-derived C1 esterase/kallikrein inhibitor versus standard care [71]; icatibant acetate versus standard care [71]; icatibant acetate versus human plasma-derived C1 esterase/kallikrein inhibitor [71]; pulmonary rehabilitation program versus isolation at home [70]; auxora (calcium release-activated calcium channel inhibitors) versus standard care [73]; umbilical cord stem cell infusion versus standard care [74]; vitamin C versus placebo [75]; sofosbuvir plus daclatasvir versus standard care [79]; sofosbuvir plus daclatasvir plus ribavirin versus hydroxychloroquine plus lopinavir-ritonavir with or without ribavirin [78]; interferon beta-1b versus standard care [80]; calcifediol versus standard care [83]; recombinant human granulocyte colony–stimulating factor versus standard care [84]; intravenous and/or nebulized electrolyzed saline with dose escalation versus standard care [99]; nasal irrigation with hypertonic saline plus surfactant versus no intervention [100]; nasal irrigation with hypertonic saline plus surfactant versus nasal irrigation with hypertonic saline [100]; nasal irrigation with hypertonic saline versus no intervention [100]; triazavirin versus placebo [101]; N-acetylcysteine versus placebo [102]; tocilizumab versus favipiravir [113].

The maximum follow-up time ranged from five [33, 34] to 60 days [89, 98] after randomization. For several of our outcomes it was not possible to conduct meta-analysis due to insufficient data.

### Corticosteroids versus control

We identified 10 trials (11 comparisons) randomizing 7,918 participants to corticosteroids versus standard care [51, 55, 86, 88, 95–98] or placebo [67, 87]. One trial was assessed at low risk of bias [87]. The remaining trials were assessed at high risk of bias (**S3 Table**). Five trials assessed the effects of methylprednisolone [55, 67, 95–97], three trials assessed the effects of dexamethasone [51, 86, 98], and two trials (three comparisons) assessed the effects of hydrocortisone [87, 88]. One trial assessing the effects of methylprednisolone was not eligible for meta-analysis, as approximately half of the participants in the experimental group were non-randomized [55]. We contacted the trial authors and asked for separate data for all randomized participants, but did not receive any response. Another trial assessing the effects of methylprednisolone was not eligible for meta-analysis, as the trial did not report on any of our review outcomes [95]. We requested data for our review outcomes from the trial authors but did not receive a response.

#### Meta-analysis of all-cause mortality

**Fig 2:**
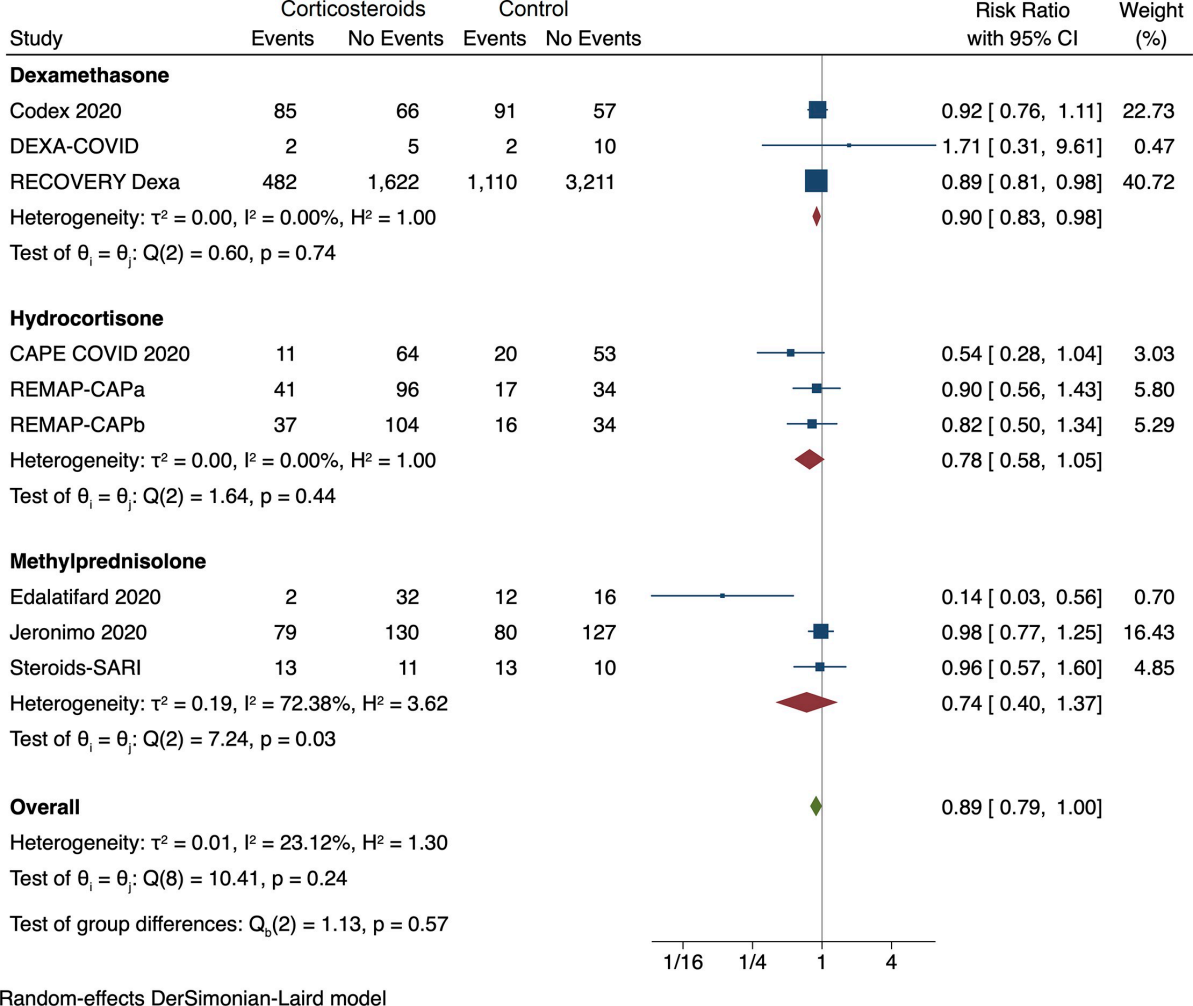
Random-effects meta-analysis for corticosteroids versus control (standard care or placebo) on all-cause mortality.

**Fig 3:**
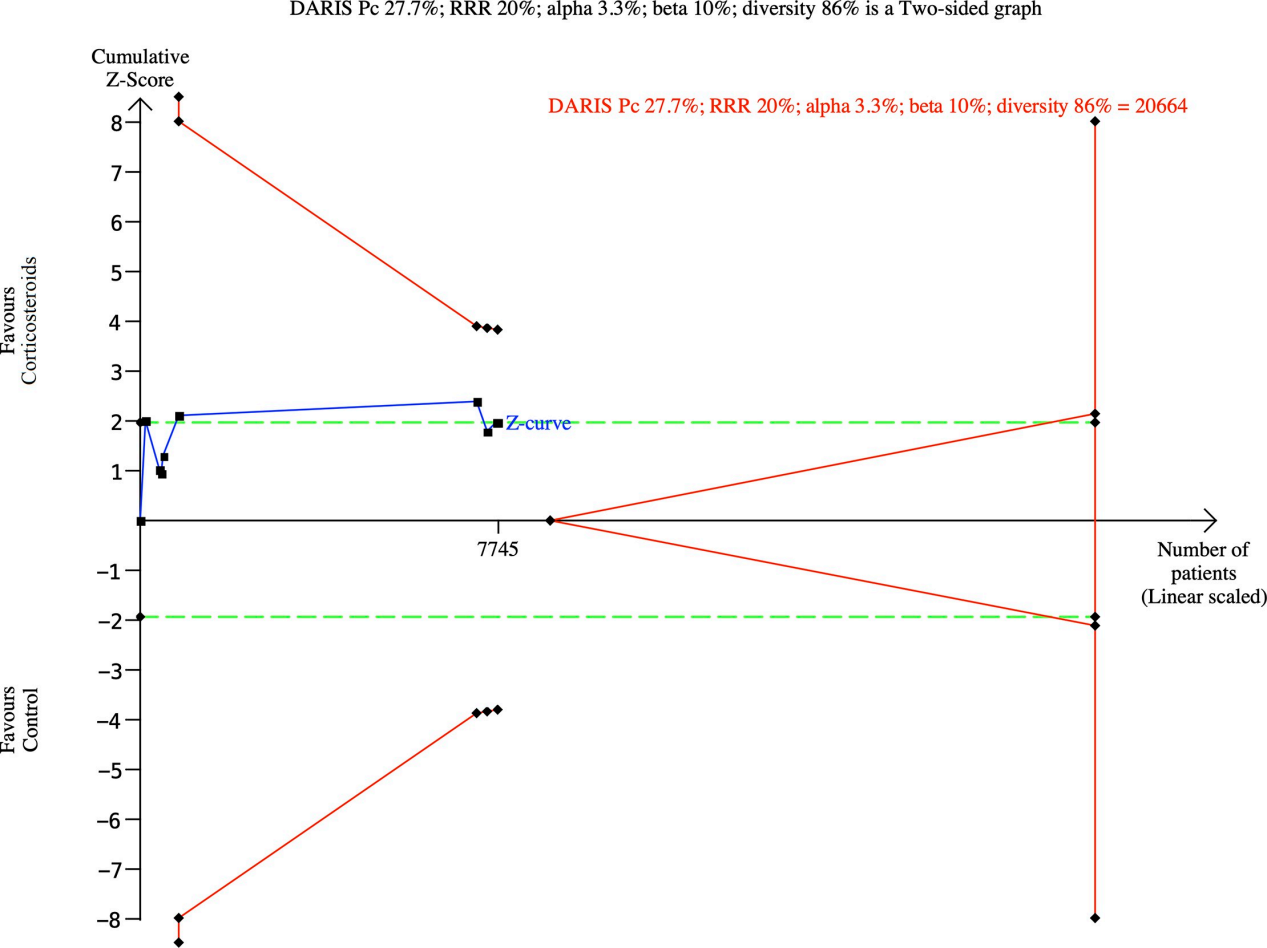
Trial sequential analysis for corticosteroids versus control (standard care or placebo) on all-cause mortality.

Random-effects meta-analysis showed no evidence of a difference between corticosteroids and control on all-cause mortality (RR 0.89; 95% CI 0.79 to 1.00; *p* = 0.05) (**Fig 2, S4 Table**). Fixed-effect meta-analysis showed evidence of a beneficial effect of corticosteroids versus control on all-cause mortality (RR 0.88; 95% CI 0.82 to 0.95; *p* = 0.00) (**S1 Fig**). Visual inspection of the forest plot and measures to quantify heterogeneity (I^2^ = 23.1%) indicated no substantial heterogeneity. The time-points of assessment varied from 21 [87] to 30 days after randomization [96, 116]. The trial sequential analysis showed that we did not have enough information to confirm or reject that corticosteroids versus control reduce the risk of all-cause mortality with a relative risk reduction of 20% (**Fig 3**). The subgroup analysis assessing the effects of the different corticosteroids versus control showed no significant subgroup differences (*p* = 0.57) (**Fig 2**). The subgroup analysis assessing the effects of disease severity as defined by trialists (mild, moderate, severe, or a combination) showed no significant subgroup differences (*p* = 0.42) (**S2 Fig**).

#### Meta-analysis of serious adverse events

Random-effects meta-analysis showed no evidence of a difference between corticosteroids and control on serious adverse events (RR 0.89; 95% CI 0.80 to 0.99; *p* = 0.04) (**S3 Fig, S4 Table**). Fixed-effect meta-analysis showed evidence of a beneficial effect of corticosteroids versus control on serious adverse events (RR 0.88; 95% CI 0.82 to 0.95; *p* = 0.00) (**S4 Fig**). Visual inspection of the forest plot and measures to quantify heterogeneity (I^2^ = 39.1%) indicated no substantial heterogeneity. The time-points of assessment varied from 21 [87] to 30 days after randomization [96, 116]. The trial sequential analysis showed that we did not have enough information to confirm or reject that corticosteroids versus control reduce the risk of serious adverse events with a relative risk reduction of 20% (**S5 Fig**). The subgroup analysis assessing the effects of the different corticosteroids versus control showed no significant subgroup differences (*p* = 0.71) (**S3 Fig**). The serious adverse event data is predominately based on mortality data, and assessed according to the ICH-GCP definition of a serious adverse event [15].

#### Meta-analysis of mechanical ventilation

Random-effects meta-analysis showed no evidence of a difference between corticosteroids versus control on mechanical ventilation (RR 0.86; 95% CI 0.55 to 1.33; *p* = 0.49) (**S6 Fig, S4 Table**). Fixed-effect meta-analysis showed evidence of a beneficial effect of corticosteroids versus control on mechanical ventilation (RR 0.77; 95% CI 0.63 to 0.94; *p* = 0.01) (**S7 Fig**). Visual inspection of the forest plot and measures to quantify heterogeneity (I^2^ = 55.3%) indicated moderate heterogeneity. The time-points of assessment varied from 7 days [67] to 28 days after randomization [46]. The trial sequential analysis showed that we did not have enough information to confirm or reject that corticosteroids versus control reduce the risk of receiving mechanical ventilation with a relative risk reduction of 20% (**S8 Fig**). The subgroup analysis assessing the effects of the different corticosteroids versus control showed no significant subgroup differences (*p* = 0.13) (**S6 Fig**). One of the two trials [67] had substantial missing data for this outcome, but it was a small trial that did not contribute with much data compared to the second trial.

### Remdesivir versus control

We identified four trials randomizing 7,370 participants to remdesivir versus standard care [85, 109] or placebo [42, 64]. All trials were assessed at high risk of bias (**S3 Table**). One trial assessed two different dosages of remdesivir versus standard care [85], and the two comparisons were both included in the meta-analysis. We halved the control group to avoid double counting [11].

#### Meta-analysis of all-cause mortality

**Fig 4:**
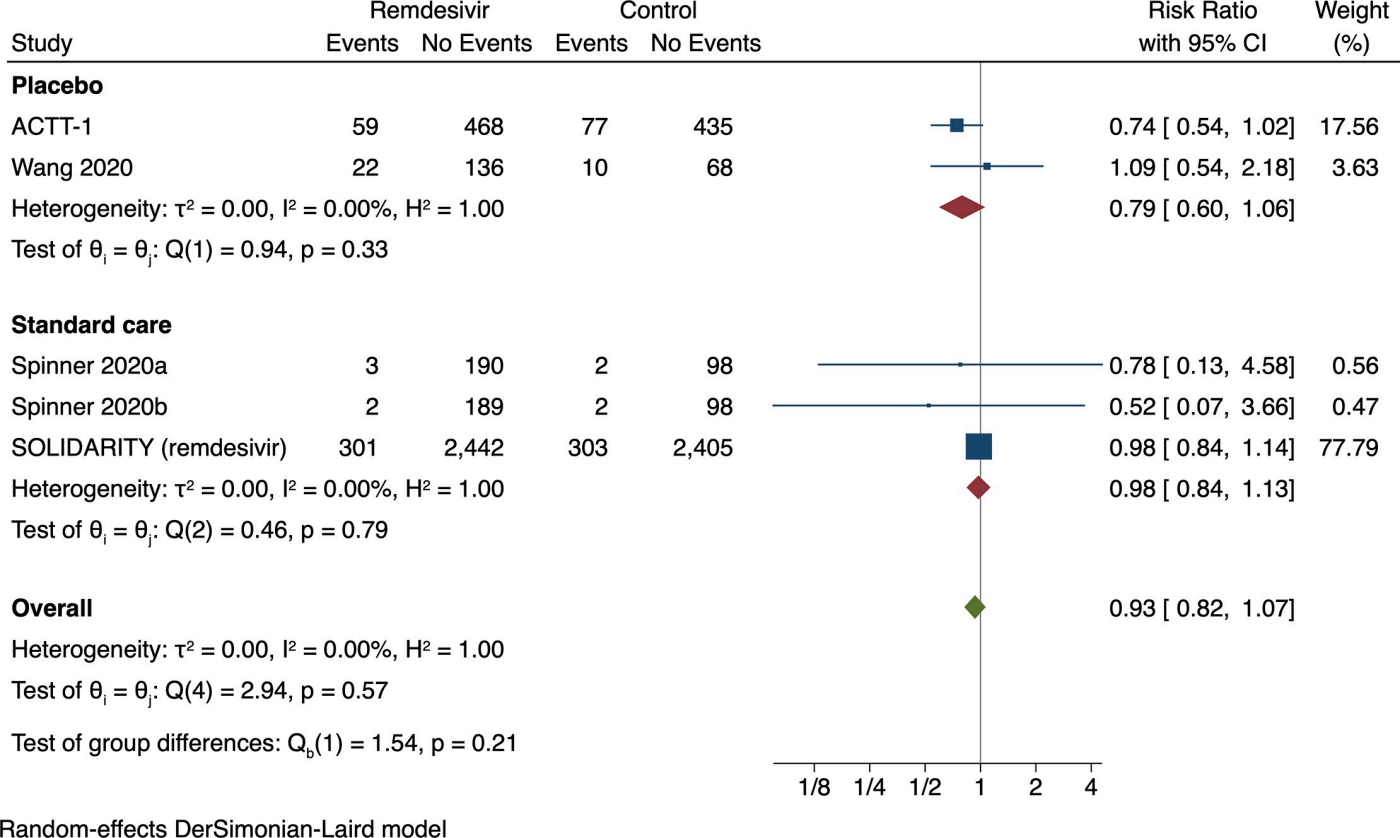
Random-effects meta-analysis for remdesivir versus control (standard care or placebo) on all-cause mortality.

**Fig 5:**
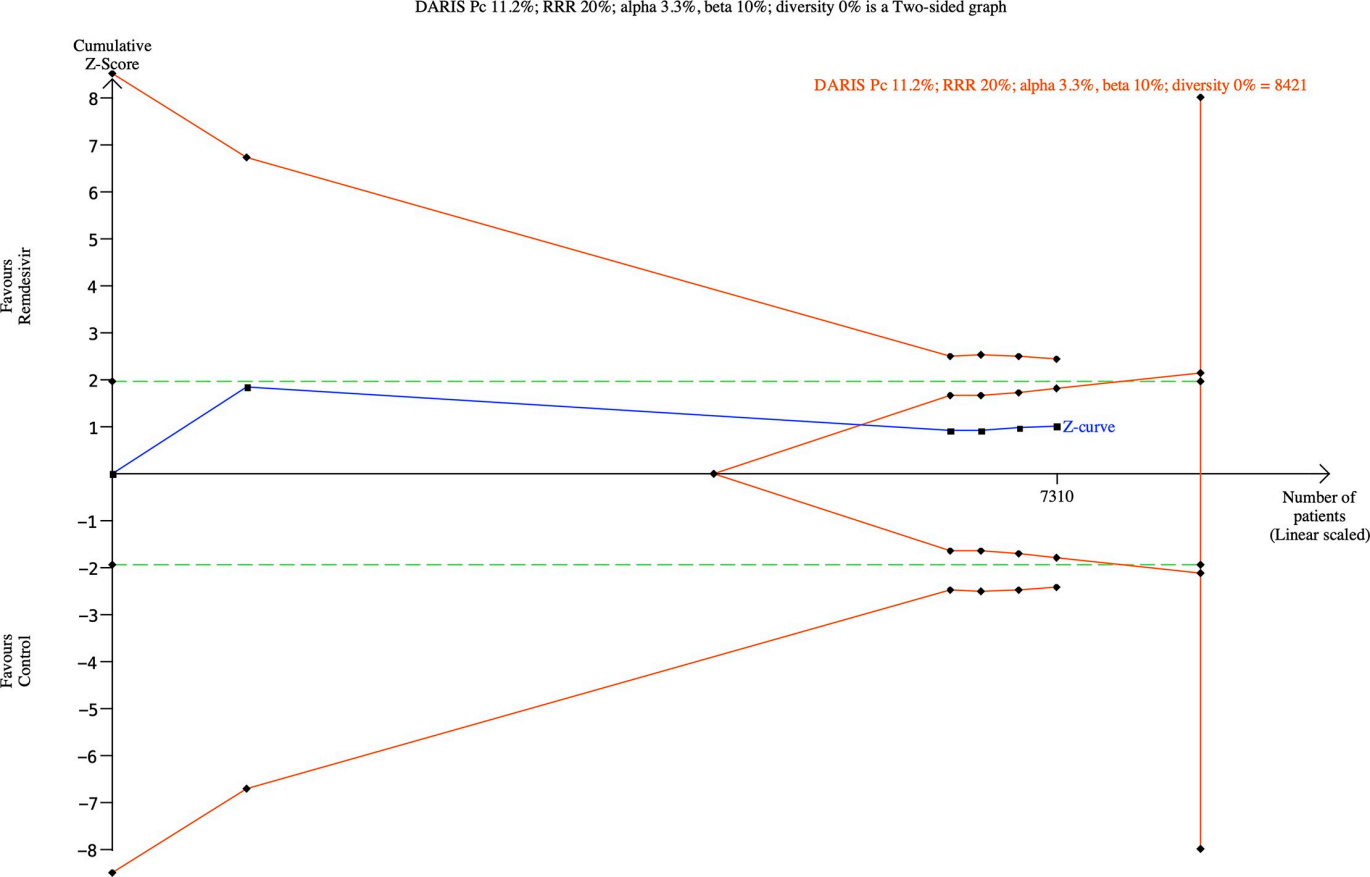
Trial sequential analysis for remdesivir versus control (standard care or placebo) on all-cause mortality.

Random-effects meta-analysis showed no evidence of a difference between remdesivir versus control on all-cause mortality (RR 0.93; 95% CI 0.82 to 1.07; *p* = 0.31) (**Fig 4**, **S5 Table**). Visual inspection of the forest plot and measures to quantify heterogeneity (I^2^ = 0%) indicated no heterogeneity. The assessment time points were 28 [42, 85, 109] and 29 days after randomization [64]. The trial sequential analysis showed that we had enough information to reject that remdesivir versus control reduces the risk of all-cause mortality with a relative risk reduction of 20% (**Fig 5**). The subgroup analysis assessing the effects of the different control interventions showed no significant subgroup differences (*p* = 0.21) (**Fig 4**). The subgroup analysis assessing the effects of early versus late intervention (defined as no oxygen versus oxygen or respiratory support at baseline) showed no significant subgroup differences (*p* = 0.85) (**S9 Fig**).

#### Meta-analysis of serious adverse events

Random-effects meta-analysis showed no evidence of difference between remdesivir versus control on serious adverse events (RR 0.82; 95% CI 0.68 to 1.00; *p* = 0.05) (**S10 Fig, S5 Table**). Fixed-effect meta-analysis showed evidence of a beneficial effect of remdesivir versus control on serious adverse events (RR 0.88; 95% CI 0.79 to 0.99; *p* = 0.03) (**S11 Fig**). Visual inspection of the forest plot and measures to quantify heterogeneity (I^2^ = 38.9%) indicated some heterogeneity. The assessment time points were 28 [42, 85, 109] and 29 days after randomization [64]. The trial sequential analysis showed that we did not have enough information to confirm or reject that remdesivir versus control reduces the risk of serious adverse events with a relative risk reduction of 20% (**S12 Fig**). The subgroup analysis assessing the effects of the different control interventions showed no significant subgroup differences (*p* = 0.83) (**S10 Fig**).

#### Meta-analysis of mechanical ventilation

Random-effects meta-analysis showed no evidence of a difference between remdesivir versus control on mechanical ventilation (RR 0.73; 95% CI 0.42 to 1.27; *p* = 0.27) (**S13 Fig, S5 Table**). Visual inspection of the forest plot and measures to quantify heterogeneity (I^2^ = 83.1%) indicated substantial heterogeneity. The assessment time points were 28 [42, 109] and 29 days after randomization [64]. The trial sequential analysis showed that we did not have enough information to confirm or reject that remdesivir versus control reduces the risk of receiving mechanical ventilation with a relative risk reduction of 20% (**S14 Fig**). The subgroup analysis assessing the effects of the different control interventions showed evidence of a significant subgroup difference between placebo and standard care (*p* = 0.00) (**S13 Fig**).

#### Meta-analysis of non-serious adverse events

Fixed-effect meta-analysis showed no evidence of a difference between remdesivir versus control on non-serious adverse events (RR 0.99; 95% CI 0.91 to 1.08; *p* = 0.86) (**S15 Fig, S5 Table**). Visual inspection of the forest plot and measures to quantify heterogeneity (I^2^ = 56.4%) indicated moderate heterogeneity. The assessment time point was 28 days after randomization [42, 64, 85]. The Trial Sequential Analysis showed that we had enough information to reject that remdesivir versus control reduces the risk of non-serious adverse events with a relative risk reduction of 20% (**S16 Fig**). The subgroup analysis assessing the effects of the different control interventions showed evidence of subgroup difference between placebo and standard care (*p* = 0.02) (**S15 Fig**).

### Hydroxychloroquine versus control

We identified 13 trials randomizing 10,276 participants to hydroxychloroquine versus standard care [33, 34, 41, 47, 53, 54, 57, 58, 104, 109], or placebo [52, 107]. All trials were assessed at high risk of bias (**S3 Table**). One trial was not eligible for meta-analysis, as the results were not reported in a usable way; i.e., the results were reported as per-protocol and several participants crossed over [41].

#### Meta-analysis of all-cause mortality

Fixed-effect meta-analysis showed no evidence of a difference between hydroxychloroquine versus control on all-cause mortality (RR 1.09; 95% CI 0.99 to 1.20; *p* = 0.08) (**S17 Fig, S6 Table**). Visual inspection of the forest plot and measures to quantify heterogeneity (I^2^ = 0%) indicated no heterogeneity. The assessment time points varied from five days after randomization [33, 34] to 30 days after randomization [107]. The trial sequential analysis showed that we had enough information to reject that hydroxychloroquine versus control reduces the risk of all-cause mortality with a relative risk reduction of 20% (**S18 Fig**). The subgroup analysis assessing the effects of hydroxychloroquine versus different control interventions showed no significant subgroup differences (*p* = 0.92) (**S17 Fig**).

#### Meta-analysis of serious adverse events

Fixed-effect meta-analysis showed no evidence of a difference between hydroxychloroquine versus control on serious adverse events (RR 1.08; 95% CI 0.98 to 1.19; *p* = 0.11) (**S19 Fig, S6 Table**). Visual inspection of the forest plot and measures to quantify heterogeneity (I^2^ = 0%) indicated no heterogeneity. The assessment time points varied from five days after randomization [33, 34] to 30 days after randomization [107]. The trial sequential analysis showed that we had enough information to reject that hydroxychloroquine versus control reduces the risk of serious adverse events with a relative risk reduction of 20% (**S20 Fig**). The subgroup analysis assessing the effects of hydroxychloroquine versus different control interventions showed no significant subgroup differences (*p* = 0.90) (**S19 Fig**).

#### Meta-analysis of admission to intensive care

Fixed-effect meta-analysis showed no evidence of a difference between hydroxychloroquine versus control on admission to intensive care (RR 0.74; 95% CI 0.44 to 1.25; *p* = 0.26) (**S21 Fig, S6 Table**). Visual inspection of the forest plot and measures to quantify heterogeneity (I^2^ = 0%) indicated no substantial heterogeneity. The assessment time points were 28 days after randomization [76] and 30 days after randomization [107]. The trial sequential analysis showed that we did not have enough information to confirm or reject that hydroxychloroquine versus control reduces the risk of admission to intensive care with a relative risk reduction of 20% (**S22 Fig**). The subgroup analysis assessing the effects of hydroxychloroquine versus different control interventions showed no significant subgroup differences (*p* = 0.61) (**S21 Fig**).

#### Meta-analysis of mechanical ventilation

Fixed-effect meta-analysis showed no evidence of a difference between hydroxychloroquine versus control on mechanical ventilation (RR 1.10; 95% CI 0.84 to 1.45; *p* = 0.48) (**S23 Fig, S6 Table**). Visual inspection of the forest plot and measures to quantify heterogeneity (I^2^ = 0%) indicated no heterogeneity. The assessment time points were 15 days after randomization [53] and 30 days after randomization [107]. The trial sequential analysis showed that we did not have enough information to confirm or reject that hydroxychloroquine versus control reduces the risk of receiving mechanical ventilation with a relative risk reduction of 20% (**S24 Fig**). The subgroup analysis assessing the effects of hydroxychloroquine versus different control interventions showed no significant subgroup differences (*p* = 0.84) (**S23 Fig**).

#### Meta-analysis of non-serious adverse events

Random-effects meta-analysis showed evidence of a harmful effect of hydroxychloroquine versus control on non-serious adverse events (RR 2.09; 95% CI 1.14 to 3.80; *p* = 0.02) (**S25 Fig, S6 Table**). Visual inspection of the forest plot and measures to quantify heterogeneity (I^2^ = 92.1%) indicated substantial heterogeneity. The assessment time points were five days after randomization [33] and 30 days after randomization [107]. The trial sequential analysis showed that we did not have enough information to confirm or reject that hydroxychloroquine versus control reduces the risk of non-serious adverse events with a relative risk reduction of 20%. The subgroup analysis assessing the effects of hydroxychloroquine versus different control interventions showed a significant subgroup difference between standard care and placebo (*p* = 0.39) (**S25 Fig**).

### Lopinavir-ritonavir versus control

We identified four trials randomizing 8,081 participants to lopinavir-ritonavir versus standard care [3, 39, 105, 109]. We also identified one trial randomizing 60 participants to lopinavir-ritonavir and novaferon versus novaferon alone [44]. All trials were assessed at high risk of bias (**S3 Table**).

#### Meta-analysis of all-cause mortality

Fixed-effect meta-analysis showed no evidence of a difference between lopinavir-ritonavir versus control on all-cause mortality (RR 1.01; 95% CI 0.92 to 1.12; *p* = 0.77) (**S26 Fig, S7 Table**). Visual inspection of the forest plot and measures to quantify heterogeneity (I^2^ = 0.0%) indicated no heterogeneity. The time-points of assessment were nine days after randomization in one trial [44], 21 days after randomization in one trial [39], 28 days after randomization in two trials [3, 109], and 28 days or until discharge or death in one trial [105]. The trial sequential analysis showed that we had enough information to reject that lopinavir-ritonavir versus control reduces the risk of all-cause mortality with a relative risk reduction of 20% (**S27 Fig**). The subgroup analysis assessing the effects of lopinavir-ritonavir in combination with novaferon versus lopinavir-ritonavir alone showed no significant subgroup differences (p = 0.99) (**S26 Fig**).

#### Meta-analysis of serious adverse events

Random-effects meta-analysis showed no evidence of a difference between lopinavir-ritonavir versus control on serious adverse events (RR 1.00; 95% CI 0.91 to 1.11; *p* = 0.93) (**S28 Fig, S7 Table**). Visual inspection of the forest plot and measures to quantify heterogeneity (I^2^ = 1.2%) indicated no substantial heterogeneity. The time-points of assessment were nine days after randomization in one trial [44], 21 days after randomization in one trial [39], 28 days after randomization in two trials [3, 109], and 28 days or until discharge or death in one trial [105]. The trial sequential analysis showed that we had enough information to reject that lopinavir-ritonavir versus control reduces the risk of serious adverse events with a relative risk reduction of 20% (**S29 Fig**). The subgroup analysis assessing the effects of lopinavir-ritonavir in combination with novaferon versus lopinavir-ritonavir alone showed no significant subgroup differences (p = 0.99) (**S28 Fig**).

#### Meta-analysis of mechanical ventilation

Random-effects meta-analysis showed no evidence of a difference between lopinavir-ritonavir versus control on mechanical ventilation (RR 1.08; 95% CI 0.94 to 1.25; *p* = 0.29) (**S30 Fig, S7 Table**). Visual inspection of the forest plot and measures to quantify heterogeneity (I^2^ = 0.0%) indicated no heterogeneity. The time-points of assessment were 28 days after randomization in two trials [3, 78] and 28 days or until discharge or death for one trial [105]. The trial sequential analysis showed that we had enough information to reject that lopinavir-ritonavir versus control reduces the risk of receiving mechanical ventilation with a relative risk reduction of 20% (**S31 Fig**).

#### Meta-analysis of renal replacement therapy

Random-effects meta-analysis showed no evidence of a difference between lopinavir-ritonavir versus control on renal replacement therapy (RR 0.97; 95% CI 0.73 to 1.28; *p* = 0.81) (**S32 Fig, S7 Table**). Visual inspection of the forest plot and measures to quantify heterogeneity (I^2^ = 0.0%) indicated no heterogeneity. The time-points of assessment was 28 days for the first trial [3] and 28 days or until discharge or death for the second trial [105]. The trial sequential analysis showed that we did not have enough information to confirm or reject that lopinavir-ritonavir versus control reduces the risk of renal replacement therapy with a relative risk reduction of 20% (**S33 Fig**).

#### Meta-analysis of non-serious adverse events

Random-effects meta-analysis showed no evidence of a difference between lopinavir–ritonavir versus standard care on non-serious adverse events (RR 1.14; 95% CI 0.85–1.53; *p* = 0.38) (**S34 Fig**; **S7 Table**). Visual inspection of the forest plot and measures to quantify heterogeneity (I^2^ = 75%) indicated substantial heterogeneity. The assessment time point was 21 days after randomization in the first trial [39] and 28 days after randomization in the second trial [3]. The trial sequential analysis showed that we did not have enough information to confirm or reject that lopinavir–ritonavir compared with standard care reduces nonserious adverse events with a relative risk reduction of 20%.

### Interferon β-1a versus control

We identified two trials randomizing 4,219 participants to interferon β-1a versus standard care [35, 109]. In one of the trials, the first 1,200 participants received interferon β-1a and lopinavir-ritonavir or lopinavir-ritonavir alone, while the remaining 2,927 participants received interferon β-1a or standard care [109]. All trials were assessed at high risk of bias (**S3 Table**).

#### Meta-analysis of all-cause mortality

Random-effects meta-analysis showed no evidence of a difference between interferon β-1a versus standard care on all-cause mortality (RR 0.75; 95% CI 0.30 to 1.88; *p* = 0.54) (**S35 Fig, S8 Table**). Visual inspection of the forest plot and measures to quantify heterogeneity (I^2^ = 84.1%) indicated substantial heterogeneity. The time-point of assessment was 28 days after randomization in both trials [35, 109]. The trial sequential analysis showed that we did not have enough information to confirm or reject that interferon β-1a versus standard care reduces the risk of all-cause mortality with a relative risk reduction of 20%.

#### Meta-analysis of serious adverse events

Random-effects meta-analysis showed no evidence of a difference between interferon β-1a versus standard care on serious adverse events (RR 0.75; 95% CI 0.30 to 1.88; *p* = 0.54) (**S36 Fig, S8 Table**). However, the data was solely based on all-cause mortality data, since no other serious adverse events were reported [15]. Visual inspection of the forest plot and measures to quantify heterogeneity (I^2^ = 84.1%) indicated substantial heterogeneity. The time-point of assessment was 28 days after randomization in both trials [35, 109]. The trial sequential analysis showed that we did not have enough information to confirm or reject that interferon β-1a versus standard care reduces the risk of serious adverse events with a relative risk reduction of 20%.

### Convalescent plasma versus control

We identified four trials randomizing 734 participants to convalescent plasma versus standard care [38, 50, 77, 90]. All trials were assessed as at high risk of bias (**S3 Table**).

#### Meta-analysis of all-cause mortality

Random-effects meta-analysis showed no evidence of difference between convalescent plasma versus standard care on all-cause mortality (RR 0.77; 95% CI 0.47 to 1.24; *p* = 0.28) (**S37 Fig, S9 Table**). Visual inspection of the forest plot and measures to quantify heterogeneity (I^2^ = 27.5%) indicated some heterogeneity. The outcome was assessed 28 days after randomization in two trials [38, 90], at 29 days after randomization in one trial [77] and up to hospital discharge or 60 days in one trial [50]. The trial sequential analysis showed that we did not have enough information to confirm or reject that convalescent plasma versus standard care reduces the risk of all-cause mortality with a relative risk reduction of 20% (**S38 Fig**).

#### Meta-analysis of serious adverse events

Fixed-effect meta-analysis showed no evidence of a difference between convalescent plasma versus standard care on serious adverse events (RR 0.93; 95% CI 0.64 to 1.35; *p* = 0.70) (**S39 Fig, S9 Table**). Visual inspection of the forest plot and measures to quantify heterogeneity (I^2^ = 0.0%) indicated no substantial heterogeneity. The time-point of assessment was 28 days after randomization in two trials [38, 90], 29 days after randomization in one trial [77], and up to hospital discharge or 60 days in the last trial [50]. The trial sequential analysis showed that we did not have enough information to confirm or reject that convalescent plasma versus standard care reduces the risk of serious adverse events with a relative risk reduction of 20% (**S40 Fig**).

### Azithromycin versus control

We identified three trials randomizing 996 participants to azithromycin versus standard care [82] or versus co-interventions with standard care [53], or without standard care [81]. All trials were assessed at high risk of bias (**S3 Table**). One trial assessed the effects of azithromycin versus standard care [53], one trial assessed the effects of azithromycin plus lopinavir-ritonavir and hydroxychloroquine versus lopinavir-ritonavir and hydroxychloroquine alone [81], and one trial assessed the effects of azithromycin plus hydroxychloroquine and standard care versus hydroxychloroquine and standard care alone [53].

#### Meta-analysis of all-cause mortality

Fixed-effect meta-analysis showed no evidence of a difference between azithromycin versus control on all-cause mortality (RR 0.99; 95% CI 0.79 to 1.25; *p* = 0.95) (**S41 Fig, S10 Table**). Visual inspection of the forest plot and measures to quantify heterogeneity (I^2^ = 7.4%) indicated no substantial heterogeneity. The time-point of assessment was 15 days after randomization in the first trial [53], 29 days after randomization in the second trial [82], and unclear in the third trial [81]. We have contacted the trial authors and requested information on the assessment time-point, but we have not received a response yet. The trial sequential analysis showed that we did not have enough information to confirm or reject that azithromycin versus control reduces the risk of all-cause mortality with a relative risk reduction of 20% (**S42 Fig**). The subgroup analysis assessing the effects of azithromycin versus different control interventions showed no significant subgroup differences (*p* = 0.35) (**S41 Fig**).

#### Meta-analysis of serious adverse events

Random-effects meta-analysis showed no evidence of a difference between azithromycin versus control on serious adverse events (RR 0.95; 95% CI 0.55 to 1.63; *p* = 0.84) (**S43 Fig, S10 Table**). Visual inspection of the forest plot and measures to quantify heterogeneity (I^2^ = 16%) indicated no substantial heterogeneity. The time-point of assessment was 15 days after randomization in the first trial [53], and 29 days after randomization in the second trial [82], and unclear in the third trial [51]. We have contacted the trial authors and requested information on the assessment time-point, but we have not received a response yet. The trial sequential analysis showed that we did not have enough information to confirm or reject that azithromycin versus control reduces the risk of serious adverse events with a relative risk reduction of 20%. The subgroup analysis assessing the effects of azithromycin versus different control interventions showed no significant subgroup differences (*p* = 0.30) (**S43 Fig**).

#### Meta-analysis of mechanical ventilation

Fixed-effect meta-analysis showed no evidence of a difference between azithromycin versus control on mechanical ventilation (RR 1.07; 95% CI 0.59 to 1.94; *p* = 0.83 (**S44 Fig, S10 Table**). Visual inspection of the forest plot and measures to quantify heterogeneity (I^2^ = 53%) indicated moderate heterogeneity. The time-point of assessment was 15 days after randomization in the first trial [53], and unclear in the second trial [81]. We have contacted the trial authors and requested information on the assessment time-point, but we have not received a response yet. The trial sequential analysis showed that we did not have enough information to confirm or reject that azithromycin versus control reduces the risk of receiving mechanical ventilation with a relative risk reduction of 20%. The subgroup analysis assessing the effects of azithromycin versus different control interventions showed no significant subgroup differences (*p* = 0.15) (**S44 Fig**).

#### Meta-analysis of non-serious adverse events

Fixed-effect meta-analysis showed no evidence of a difference between azithromycin versus control on non-serious adverse events (RR 1.09; 95% CI 0.89 to 1.34; *p* = 0.38) (**S45 Fig, S10 Table**). Visual inspection of the forest plot and measures to quantify heterogeneity (I^2^ = 0%) indicated no heterogeneity. The time-point of assessment was 15 days after randomization in the first trial [53], and 29 days after randomization in the second trial [82]. The trial sequential analysis showed that we did not have enough information to confirm or reject that azithromycin versus control reduces the risk of non-serious adverse events with a relative risk reduction of 20% (**S46 Fig**). The subgroup analysis assessing the effects of azithromycin versus different control interventions showed no significant subgroup differences (*p* = 0.71) (**S45 Fig**).

### Colchicine versus control

We identified three trials randomizing 248 participants to colchicine versus standard care [48], placebo plus standard care [91], or placebo plus hydroxychloroquine [106]. In the latter trial, the colchicine group also received hydroxychloroquine as a co-intervention [106]. All trials were assessed as at high risk of bias (**S3 Table**).

#### Meta-analysis of all-cause mortality

Fixed-effect meta-analysis showed no evidence of a difference between colchicine versus control on all-cause mortality (RR 1.03; 95% CI 0.07 to 16.01; *p* = 0.98) (**S47 Fig, S11 Table**). Visual inspection of the forest plot and measures to quantify heterogeneity (I^2^ = 0%) indicated no heterogeneity. The time-point of assessment was unclear in both trials [91, 106]. We have contacted the trial authors and requested information on the assessment time-points, but we have not received a response yet. The trial sequential analysis showed that we did not have enough information to confirm or reject that colchicine versus control reduces the risk of all-cause mortality with a relative risk reduction of 20%. The subgroup analysis assessing the effects of colchicine versus different control interventions showed no evidence of a significant subgroup difference (*p* = 0.98) (**S47 Fig**).

#### Meta-analysis of non-serious adverse events

Random-effects meta-analysis showed no evidence of a difference between colchicine versus control on non-serious adverse events (RR 0.88; 95% CI 0.18 to 4.39; *p* = 0.87) (**S48 Fig, S11 Table**). Visual inspection of the forest plot and measures to quantify heterogeneity (I^2^ = 79.1%) indicated substantial heterogeneity. The time-point of assessment was 21 days after randomization in one trial [48], but unclear in the other two trials [91, 106]. We have contacted the trial authors and requested information on the assessment time-points, but we have not received a response yet. The trial sequential analysis showed that we did not have enough information to confirm or reject that colchicine versus control reduces the risk of non-serious adverse events with a relative risk reduction of 20%. The subgroup analysis assessing the effects of colchicine versus different control interventions showed evidence of significant subgroup differences (*p* = 0.01) (**S48 Fig**).

### Intravenous immunoglobin versus control

We identified two trials randomizing 93 participants to intravenous immunoglobulin versus standard care [56] or placebo [94]. Both trials included immunoglobulin from healthy donors [56, 94]. Both trials were assessed at high risk of bias (**S3 Table**).

#### Meta-analysis of all-cause mortality

Fixed-effect meta-analysis showed evidence of a beneficial effect of intravenous immunoglobulin versus control on all-cause mortality (RR 0.40; 95% CI 0.19 to 0.87; *p* = 0.02) (**S49 Fig, S12 Table**). Visual inspection of the forest plot and measures to quantify heterogeneity (I^2^ = 0%) indicated no heterogeneity. The outcome was assessed only in hospital in the first trial [94] and up to 30 days in the second trial [56]. The trial sequential analysis showed that we did not have enough information to confirm or reject that intravenous immunoglobulin versus control reduces the risk of all-cause mortality with a relative risk reduction of 20% (**S50 Fig**). The subgroup analysis assessing the effects of different control interventions showed no evidence of a significant subgroup difference between placebo and standard care (*p* = 0.89) (**S49 Fig**).

#### Meta-analysis of serious adverse events

Fixed-effect meta-analysis showed evidence of a beneficial effect of intravenous immunoglobulin versus control on serious adverse events (RR 0.40; 95% CI 0.19 to 0.87; *p* = 0.02) (**S51 Fig, S12 Table**). This data is solely based on all-cause mortality data according to the ICH-GCP guidelines [15], since no other serious adverse events were reported. Visual inspection of the forest plot and measures to quantify heterogeneity (I^2^ = 0%) indicated no heterogeneity. The outcome was assessed only in hospital in the first trial [94] and up to 30 days in the second trial [56]. The trial sequential analysis showed that we did not have enough information to confirm or reject that intravenous immunoglobulin versus control reduces the risk of all-cause mortality with a relative risk reduction of 20% (**S52 Fig**). The subgroup analysis assessing the effects of different control interventions showed no evidence of a significant subgroup difference between placebo and standard care (*p* = 0.89) (**S51 Fig**).

### Tocilizumab versus control

We identified six trials randomizing 1038 patients to tocilizumab versus standard care [92, 110–112], placebo with standard care [89] or favipiravir alone as co-intervention [113]. All trials were assessed as at high risk of bias (**S3 Table**).

#### Meta-analysis of all-cause mortality

Random-effects meta-analysis showed no evidence of a difference between tocilizumab and control interventions on all-cause mortality (RR 1.03; 95% CI 0.72 to 1.46; *p* = 0.89) (**S53 Fig, S13 Table**). Visual inspection of the forest plot and measures to quantify heterogeneity (I^2^ = 0.0%) indicated no heterogeneity. The time-points of assessment were 28 days [89, 110, 112] and 30 days [111] after randomization. The trial sequential analysis showed that we did not have enough information to confirm or reject that tocilizumab versus control reduces the risk of all-cause mortality with a relative risk reduction of 20% (**S54 Fig**). The subgroup analysis assessing the effects of different control interventions showed no significant subgroup differences (p = 0.87) (**S53 Fig**).

#### Meta-analysis of serious adverse events

Random-effects meta-analysis showed no evidence of a difference between tocilizumab and control interventions on serious adverse events (RR 0.63; 95% CI 0.35 to 1.14; *p* = 0.12) (**S55 Fig, S13 Table**). Fixed-effect meta-analysis showed evidence of a beneficial effect of tocilizumab versus control on serious adverse events (RR 0.68; 95% CI 0.57 to 0.81; *p* = 0.00) (**S56 Fig**). Visual inspection of the forest plot and measures to quantify heterogeneity (I^2^ = 77.4%) indicated heterogeneity. The time-point of assessment was either unclear [89, 113], 28 days [110, 112], or 30 days [111] after randomization. The trial sequential analysis showed that we did not have enough information to confirm or reject that tocilizumab versus control reduces the risk of serious adverse events with a relative risk reduction of 20%. The subgroup analysis assessing the effects of different control interventions showed no significant subgroup differences (p = 0.13) (**S55 Fig**).

#### Meta-analysis of admission to intensive care

Random-effects meta-analysis showed no evidence of a difference between tocilizumab and control interventions on admission to intensive care (RR 0.71; 95% CI 0.37 to 1.38; *p* = 0.32) (**S57 Fig, S13 Table**). Visual inspection of the forest plot and measures to quantify heterogeneity (I^2^ = 36%) indicated no substantial heterogeneity. The time-point of assessment was either unclear [89] or 30 days [111] after randomization. The trial sequential analysis showed that we did not have enough information to confirm or reject that tocilizumab versus control reduces the risk of admission to intensive care with a relative risk reduction of 20%. The subgroup analysis assessing the effects of control interventions showed no significant subgroup differences (p = 0.21) (**S57 Fig**).

#### Meta-analysis of mechanical ventilation

Random-effects meta-analysis showed evidence of a beneficial effect of tocilizumab versus control on mechanical ventilation (RR 0.70; 95% CI 0.51 to 0.96; *p* = 0.02) (**S58 Fig, S13 Table**). Visual inspection of the forest plot and measures to quantify heterogeneity (I^2^ = 0%) indicated no heterogeneity. The time-point of assessment was either unclear [89] or 28 days [110, 112] after randomization. The trial sequential analysis showed that we did not have enough information to confirm or reject that tocilizumab reduce the risk of mechanical ventilation with a relative risk reduction of 20% (**S59 Fig**). The subgroup analysis assessing the effects of control interventions showed no significant subgroup differences (p = 0.34) (**S58 Fig**).

#### Meta-analysis of non-serious adverse events

Fixed-effect meta-analysis showed no evidence of a difference between tocilizumab versus control on non-serious adverse events (RR 1.03; 95% CI 0.92 to 1.14; *p* = 0.63) (**S60 Fig, S13 Table**). Visual inspection of the forest plot and measures to quantify heterogeneity (I^2^ = 57.9%) indicated moderate heterogeneity. The time-point of assessment was either unclear [89, 92, 113], 28 days [110], or 30 days [111] after randomization. The trial sequential analysis showed that we did not have enough information to confirm or reject that tocilizumab versus control reduces the risk of non-serious adverse events with a relative risk reduction of 20%. The subgroup analysis assessing the effects of different control interventions showed no significant subgroup differences (p = 0.27) (**S60 Fig**).

### Bromhexine versus control

We identified two trials randomizing 96 participants to bromhexine versus standard care [93, 103]. Both trials were assessed at high risk of bias (**S3 Table**).

#### Meta-analysis of all-cause mortality

Random-effects meta-analysis showed no evidence of a difference between bromhexine versus standard care on all-cause mortality (RR 0.17; 95% CI 0.02 to 1.70; *p* = 0.13) (**S61 Fig, S14 Table**). Visual inspection of the forest plot and measures to quantify heterogeneity (I^2^ = 0%) indicated no heterogeneity. The time-point of assessment was 28 days for the first trial [103] and unclear for the second trial [93]. The trial sequential analysis showed that we did not have enough information to confirm or reject that bromhexine versus standard care reduces the risk of all-cause mortality with a relative risk reduction of 20%.

#### Meta-analysis of non-serious adverse events

Random-effects meta-analysis showed evidence of a beneficial effect of bromhexine versus standard care on non-serious adverse events (RR 0.32; 95% CI 0.15 to 0.69; *p* < 0.00) (**S62 Fig, S14 Table**). Visual inspection of the forest plot and measures to quantify heterogeneity (I^2^ = 0%) indicated no heterogeneity. The time-point of assessment was 28 days for the first trial [103] and unclear for the second trial [93]. The trial sequential analysis showed that we did not have enough information to confirm or reject that bromhexine versus standard care reduces the risk of non-serious adverse events with a relative risk reduction of 20%.

### Remaining trial data

Because of lack of relevant data, it was not possible to conduct other meta-analyses, individual patient data meta-analyses, or network meta-analysis. Nine single trials showed statistically significant results but were all underpowered to confirm or reject realistic intervention effects. We post hoc defined a ‘realistic intervention effect’ as a relative risk between 0.7 and 0.9.

One trial randomizing 402 participants compared five versus ten days of remdesivir and showed evidence of a beneficial effect of five days of remdesivir on serious adverse events (*p* = 0.003 (Fisher’s exact test)) [36]. One trial randomizing 92 participants compared the immunomodulator interferon β-1a added to standard care versus standard care alone and showed evidence of a beneficial effect of interferon β-1a on all-cause mortality (*p* = 0.029) [35]. This trial also showed evidence of a harmful effect of interferon β-1a on non-serious adverse events (*p* = 0.006) [35]. One single trial randomizing 81 participants compared high-dosage versus low-dosage chloroquine diphosphate and showed evidence of a beneficial effect of low-dosage chloroquine on all-cause mortality (*p* = 0.024) [49]. One single three group trial randomizing 667 participants to hydroxychloroquine with or without azithromycin versus standard care and showed evidence of a harmful effect of hydroxychloroquine with azithromycin on adverse events not considered serious (*p* = 0.015) [53]. One single trial randomizing 76 participants compared calcifediol versus standard care and showed evidence of a beneficial effect of calcifediol on admittance to intensive care (*p* = 0.0001) [83]. One single trial randomizing 200 participants compared recombinant human granulocyte colony–stimulating factor (rhG-CSF) versus standard care and showed evidence of a beneficial effect of rhG-CSF on all-cause mortality (*p* = 0.017), and receipt of mechanical ventilation (*p* = 0.003) [84]. This trial also showed evidence of a harmful effect of rhG-CSF on non-serious adverse events (*p* = 0.0001) [84]. One single trial randomizing 84 participants compared electrolyzed saline versus standard care and showed evidence of a beneficial effect of electrolyzed saline on all-cause mortality (*p* = 0.019) [99]. One single trial randomizing 78 participants compared bromhexine hydrochloride versus standard care and showed evidence of a beneficial effect of bromhexine hydrochloride on admittance to intensive care (*p* = 0.013) and receipt of mechanical ventilation (*p* = 0.014) [103]. One single trial randomizing 100 participants compared hydroxychloroquine combined with arbidol versus hydroxychloroquine combined with lopinavir-ritonavir and showed evidence of a beneficial effect of hydroxychloroquine combined with arbidol on admission to intensive care (*p =* 0.0001) [108].

None of the remaining single trial results showed evidence of a difference on our predefined review outcomes. Two trials did not report the results in a usable way; one trial reported results of the experimental group with a proportion of participants being non-randomized [55], and the second trial reported the results as per-protocol, and there was participant crossover [41]. Seven trials did not report on our review outcomes [43, 61, 62, 66, 70, 95, 100]. We have contacted all corresponding authors, but we have not been able to obtain outcomes for our analyses from the trialists yet. Most trials were assessed at high risk of bias (**S3 Table**). Characteristics of the trials and their results on the review outcomes can be found in **S2 Table**. Certainty of the evidence was assessed as ‘low’ or ‘very low’ for all outcomes (**S15–S66 Tables**).

### Possible future contributions of ongoing trials

On November 2, 2020, a search on the Cochrane COVID-19 Study Register revealed 2527 registered randomized clinical trials [13]. From these, 106 different interventions for treatment of COVID-19 patients were identified [13]. The ten most investigated experimental interventions were hydroxychloroquine (162 trials), convalescent plasma (55 trials), azithromycin (52 trials), lopinavir and ritonavir (40 trials), tocilizumab (33 trials), chloroquine (30 trials), favipiravir (24 trials), remdesivir (15 trials), sarilumab (15 trials), and dexamethasone (13 trials). Eligible trials will continuously be included in the present living systematic review once results become available.

## Discussion

We conducted the second edition of our living systematic review assessing the beneficial and harmful effects of any intervention for COVID-19. We searched relevant databases and websites for published and unpublished trials until November 2, 2020. We included a total of 82 trials randomizing 40,249 participants. Our present study showed that no evidence-based treatment for COVID-19 currently exists.

Very low certainty evidence indicated that corticosteroids might reduce the risk of death, serious adverse events, and mechanical ventilation.

Moderate certainty evidence showed that we could reject that remdesivir reduces the risk of death by 20%. Very low certainty evidence indicated that remdesivir might reduce the risk of serious adverse events.

Very low certainty evidence indicated that intravenous immunoglobin might reduce the risk of death and serious adverse events, that tocilizumab might reduce the risk of serious adverse events and mechanical ventilation, and that bromhexine might reduce the risk of non-serious adverse events.

Moderate certainty evidence showed that we could reject that hydroxychloroquine reduces the risk of death and serious adverse events by 20%, and that we could reject that lopinavir-ritonavir reduces the risk of death, serious adverse events, and risk of mechanical ventilation by 20%.

Otherwise, we could neither confirm nor reject the effects of other interventions for COVID-19. More trials with low risks of bias and random errors are urgently needed. For several interventions we found a large number of currently ongoing trials.

The present review concludes that no evidence-based treatment currently exists for COVID-19. Previous studies [116–118] including our first edition of the present review [118] have concluded that both corticosteroids and remdesivir showed promising results. Since our last edition, we have included 49 more trials randomizing 26.937 more participants, and we therefore have more information, causing this difference. One previous systematic review published in JAMA assessed the association between corticosteroids and 28-day all-cause mortality and concluded that corticosteroids are effective for treating critically ill patients with COVID-19 in reducing all-cause mortality [116]. However, the conclusions of this review are limited to critically ill patients. This review assessed the certainty of evidence for all-cause mortality to be moderate, while we assessed the certainty of evidence to be very low (see **S4 Table**).

Our present results showed a discrepancy between the random-effects meta-analysis result and the fixed-effect meta-analysis result (due to heterogeneity) of corticosteroids versus control interventions when assessing all-cause mortality, i.e., the fixed-effect meta-analysis indicated a more beneficial effect of corticosteroids. Due to the discrepancy between the random-effects and the fixed-effect model we believe that these results should be interpreted with great caution considering the uncertainty of the evidence. Furthermore, the meta-analytic effect estimate was 0.89 which may be considered relatively small.

The U.S. Food and Drug Administration (FDA) recently approved remdesivir for use in adult and pediatric patients 12 years of age and older for the treatment of COVID-19 requiring hospitalization [119]. Based on the current evidence, we conclude that remdesivir is not effective in reducing all-cause mortality, neither for patients not requiring oxygen nor for patients requiring oxygen or respiratory support at baseline. There was a discrepancy between the random-effects and the fixed-effect meta-analysis (due to heterogeneity) of remdesivir versus control on serious adverse events, i.e., the fixed-effect meta-analysis indicated a more beneficial effect of remdesivir. Due to the discrepancy between the random-effects and the fixed-effect model we believe that these results should be interpreted with great caution considering the uncertainty of the evidence. On all other outcomes when assessing the effects of remdesivir, we conclude that more information is needed to confirm or reject the effects of remdesivir. Hence, the clinical effects of remdesivir are unclear based on current evidence.

Our results are similar to the results of a preprint of an international collaborative meta-analysis of randomized clinical trials assessing mortality outcomes with hydroxychloroquine and chloroquine for participants with COVID-19 [120]. This review included some unpublished data [120]. We have contacted the trialists of the trials that provided unpublished data for this review, but we have not received any data yet. Nevertheless, our conclusions that hydroxychloroquine does not reduce mortality in COVID-19 patients are the same [120].

Although we could exclude an intervention effect at 20% or above for most of our interventions with our trial sequential analyses, we did not assess smaller and still worthwhile intervention effects. If patients and investigators feel that such smaller intervention effects are worth pursuing, then we recommend the conduct of trials with much larger sample sizes than the ones we have identified in the present systematic review. That will require more national and international collaboration [121].

Our living systematic review has a number of strengths. The predefined methodology was based on the Cochrane Handbook for Systematic Reviews of Interventions [11], the eight-step assessment suggested by Jakobsen et al. [17], and trial sequential analysis [22]. Hence, this review considers both risks of systematic errors and risk of random errors. Another strength is the living systematic review design, which allows us to continuously surveil and update the evidence-base of existing interventions for treatment of COVID-19 resulting in a decreased timespan from publication of our results to optimization of clinical practice. This is particularly important in this international health-care crisis, where a large number of new randomized clinical trials are continuously registered and published.

Our living systematic review also has limitations. First, the primary limitation is the paucity of trials currently available, and the results from most current meta-analyses are of low or very low certainty. This must be considered when interpreting our meta-analysis results. Second, the trials that we included were all at risks of systematic errors so our results presumably overestimate the beneficial effects and underestimate the harmful effects of the included interventions [122–129]. Third, it was not relevant to perform individual patient data meta-analyses, network-meta-analysis, or several of the planned subgroup analyses due to lack of relevant data. We contacted all trial authors requesting individual patient data, but until now we only received five datasets [37, 68, 78, 79, 106]. We did not perform network meta-analysis because the ranking of the interventions is not unclear, i.e., no evidence-based intervention currently exits for COVID-19. Fourth, we included ‘time to clinical improvement’ as an outcome post hoc. We did not initially plan to assess ‘time to clinical improvement’ [8] because this outcome is poorly defined and if outcome assessors are not adequately blinded, assessments of ‘improvement’ may be biased. Furthermore, time to clinical improvement is not one of the most patient-important outcomes. As an example, most patients would rather survive without complications than recover a few days sooner. Fifth, the included trials assessed the outcomes at different time points, which might contribute to increased heterogeneity. Sixth, some data are included from preprints, and these might be subject to change following peer-review. Therefore, some results, bias risk assessments, and GRADE summaries might change in later editions of this living systematic review following inclusion of the published peer-reviewed manuscripts. Seventh, we follow our protocol [8] as well as Cochrane Handbook for Systematic Reviews of Interventions [11], and hence, we only consider formal tests for publication bias when approximately more than 10 trials are included in a meta-analysis. Therefore, we have not performed such analysis in the present edition of the review.

We have identified two reviews that are comparable to our present project [117, 130]. The first is a network meta-analysis published in BMJ [117]. However, that review only includes drug treatments for COVID-19, does not include individual patient data meta-analyses, and does not use trial sequential analysis or similar methods to handle problems with multiplicity (repeating updating of meta-analysis, multiple comparisons due to inclusion of multiple interventions, assessing multiple outcomes). Therefore, the conclusiveness of the presented evidence in that review is unclear.

The second project is a living mapping of ongoing randomized clinical trials with network meta-analysis on all interventions for COVID-19 [130]. The authors are producing and disseminating preliminary results through an open platform [130]. This review includes both prevention and treatment and does not use trial sequential analysis or similar methods to handle problems with multiplicity [8]. Therefore, the conclusiveness of the presented evidence is unclear.

Our assessment of the certainty of the evidence primarily concerning the effects of corticosteroids and remdesivir was lower compared to the two above-mentioned similar projects. These discrepancies are primarily caused by the described differences in choice of review methods.

## Conclusions

No evidence-based treatment for COVID-19 currently exists. Very low certainty evidence indicates that corticosteroids might reduce the risk of death, serious adverse events, and mechanical ventilation; that remdesivir might reduce the risk of serious adverse events; that intravenous immunoglobin might reduce the risk of death and serious adverse events; that tocilizumab might reduce the risk of serious adverse events and mechanical ventilation, and that bromhexine might reduce the risk of non-serious adverse events. More trials with low risks of bias and random errors are urgently needed. This review will continuously inform best practice in treatment and clinical research of COVID-19.

### Differences between the protocol and the review

We erroneously reported the adjusted TSA alpha as 2% in our published protocol [8]. This has now been corrected to 3.3% according to two primary outcomes [17]. Further, we included ‘time to clinical improvement’ as an outcome post hoc.

## Supporting information

S1 Text PRISMA 2009 checklist. (DOC)

S2 Text Search strategies. (DOC)

S1 Table Excluded trials. (DOCX)

S2 Table Characteristics of included studies. (XLSX)

S3 Table Risk of bias assessments. (TIFF)

S4 Table Summary of findings table of corticosteroids versus control interventions (standard care or placebo). (DOCX)

S5 Table Summary of findings table of remdesivir versus control interventions (standard care or placebo). (DOCX)

S6 Table Summary of findings table of hydroxychloroquine versus control interventions (standard care or placebo). (DOCX)

S7 Table Summary of findings table of lopinavir-ritonavir versus control interventions (standard care or placebo). (DOCX)

S8 Table Summary of findings table of interferon beta-1a versus standard care. (DOCX)

S9 Table Summary of findings table of convalescent plasma versus control interventions (standard care or placebo). (DOCX)

S10 Table Summary of findings table of azithromycin versus control interventions (standard care or placebo). (DOCX)

S11 Table Summary of findings table of colchicine versus control interventions (standard care or placebo). (DOCX)

S12 Table Summary of findings table of intravenous immunoglobin versus control interventions (standard care or placebo). (DOCX)

S13 Table Summary of findings table of tocilizumab versus control interventions (standard care or placebo). (DOCX)

S14 Table Summary of findings table of bromhexine versus control interventions (standard care or placebo). (DOCX)

S15 Table Summary of findings table of favipiravir versus control interventions (standard care or placebo). (DOCX)

S16 Table Summary of findings table of favipiravir versus umifenovir. (DOCX)

S17 Table Summary of findings table of umifenovir versus lopinavir-ritonavir. (DOCX)

S18 Table Summary of findings table of umifenovir versus standard care. (DOCX)

S19 Table Summary of findings table of novaferon versus novaferon + lopinavir-ritonavir. (DOCX)

S20 Table Summary of findings table of novaferon + lopinavir-ritonavir versus lopinavir-ritonavir. (DOCX)

S21 Table Summary of findings table of novaferon versus lopinavir-ritonavir. (DOCX)

S22 Table Summary of findings table of alpha lipotic acid versus placebo. (DOCX)

S23 Table Summary of findings table of baloxavir marboxil versus favipiravir. (DOCX)

S24 Table Summary of findings table of baloxavir marboxil versus standard care. (DOCX)

S25 Table Summary of findings table of triple combination of interferon beta-1b + lopinavir-ritonavir + ribavirin versus lopinavir-ritonavir. (DOCX)

S26 Table Summary of findings table of remdesivir for 10 days versus remdesivir for 5 days. (DOCX)

S27 Table Summary of findings table of high-flow nasal oxygenation versus standard bag-valve oxygenation. (DOCX)

S28 Table Summary of findings table of hydroxychloroquine versus chloroquine. (DOCX)

S29 Table Summary of findings table of chloroquine versus standard care. (DOCX)

S30 Table Summary of findings table of high dosage chloroquine diphosphate versus low dosage chloroquine diphosphate. (DOCX)

S31 Table Summary of findings table of hydroxychloroquine + azithromycin versus standard care. (DOCX)

S32 Table Summary of findings table of triple combination of darunavir + cobicistat + interferon alpha-2b versus interferon alpha-2b. (DOCX)

S33 Table Summary of findings table of lopinavir-ritonavir + interferon alpha versus ribavirin + interferon alpha. (DOCX)

S34 Table Summary of findings table of ribavirin + lopinavir-ritonavir + interferon alpha versus ribavirin + interferon alpha. (DOCX)

S35 Table Summary of findings table of ribavirin + lopinavir-ritonavir + interferon alpha versus lopinavir-ritonavir + interferone alpha. (DOCX)

S36 Table Summary of findings table of Lincocin® versus Azitro®. (DOCX)

S37 Table Summary of findings table of ^99m^Tc-MDP injection versus standard care. (DOCX)

S38 Table Summary of findings table of interferon alpha-2b + interferon gamma versus interferone alpha-2b. (DOCX)

S39 Table Summary of findings table of telmisartan versus standard care. (DOCX)

S40 Table Summary of findings table of avifavir 1600/600 versus avifavir 1800/800. (DOCX)

S41 Table Summary of findings table of dexamethasone + aprepitant versus dexamethasone. (DOCX)

S42 Table Summary of findings table of anti-C5a antibody versus standard care. (DOCX)

S43 Table Summary of findings table of azvudine versus standard care. (DOCX)

S44 Table Summary of findings table of human plasma-derived C1 esterase/kallikrein inhibitor versus standard care. (DOCX)

S45 Table Summary of findings table of icatibant acetate versus standard care. (DOCX)

S46 Table Summary of findings table of icatibant acetate versus human plasma-derived C1 esterase/kallikrein inhibitor. (DOCX)

S47 Table Summary of findings table of pulmonary rehabilitation program versus isolation at home. (DOCX)

S48 Table Summary of findings table of auxora (calcium release-activated calcium channel inhibitors) versus standard care. (DOCX)

S49 Table Summary of findings table of umbilical cord stem cell infusion versus standard care. (DOCX)

S50 Table Summary of findings table of vitamin C versus placebo. (DOCX)

S51 Table Summary of findings table of sofosbuvir + daclatasvir versus standard care. (DOCX)

S52 Table Summary of findings table of sofosbuvir + daclatasvir + ribavirin versus hydroxychloroquine + lopinavir-ritonavir with or without ribavirin. (DOCX)

S53 Table Summary of findings table of interferon beta-1b versus standard care. (DOCX)

S54 Table Summary of findings table of calcifediol versus standard care. (DOCX)

S55 Table Summary of findings table of rhG-CSF versus standard care. (DOCX)

S56 Table Summary of findings table of intravenous and/or nebulized electrolyzed saline with dose escalation versus standard care. (DOCX)

S57 Table Summary of findings table of nasal irrigation with hypertonic saline + surfactant versus no intervention. (DOCX)

S58 Table Summary of findings table of nasal irrigation with hypertonic saline versus nasal irrigation with hypertonic saline + surfactant. (DOCX)

S59 Table Summary of findings table of nasal irrigation with hypertonic saline versus no intervention. (DOCX)

S60 Table Summary of findings table of triazavirin versus placebo. (DOCX)

S61 Table Summary of findings table of N-acetylcysteine versus placebo. (DOCX)

S62 Table Summary of findings table of hydroxychloroquine + arbidiol versus hydroxychloroquine + lopinavir-ritonavir. (DOCX)

S63 Table Summary of findings table of tocilizumab versus favipiravir. (DOCX)

S64 Table Summary of findings table of avifavir 1600/600 versus standard care. (DOCX)

S65 Table Summary of findings table of avifavir 1800/800 versus standard care. (DOCX)

S66 Table Summary of findings table of tocilizumab + favipiravir versus tocilizumab. (DOCX)

S1 Fig Fixed-effect meta-analysis of corticosteroids versus control interventions (standard care or placebo) on all-cause mortality. (TIFF)

S2 Fig Subgroup analysis of disease severity for corticosteroids versus control interventions (standard care or placebo) on all-cause mortality. (TIFF)

S3 Fig Random-effects meta-analysis of corticosteroids versus control interventions (standard care or placebo) on serious adverse events. (TIFF)

S4 Fig Fixed-effect meta-analysis of corticosteroids versus control interventions (standard care or placebo) on serious adverse events. (TIFF)

S5 Fig Trial sequential analysis of corticosteroids versus control interventions (standard care or placebo) on serious adverse events. (TIFF)

S6 Fig Random-effects meta-analysis of corticosteroids versus control interventions (standard care or placebo) on receipt of mechanical ventilation. (TIFF)

S7 Fig Fixed-effect meta-analysis of corticosteroids versus control interventions (standard care or placebo) on receipt of mechanical ventilation. (TIFF)

S8 Fig Trial sequential analysis of corticosteroids versus control interventions (standard care or placebo) on receipt of mechanical ventilation. (TIFF)

S9 Fig Subgroup analysis of early versus late intervention for remdesivir versus control interventions (standard care or placebo) on all-cause mortality. (TIFF)

S10 Fig Random-effects meta-analysis of remdesivir versus control interventions (standard care or placebo) on serious adverse events. (TIFF)

S11 Fig Fixed-effect meta-analysis of remdesivir versus control interventions (standard care or placebo) on serious adverse events. (TIFF)

S12 Fig Trial sequential analysis of remdesivir versus control interventions (standard care or placebo) on serious adverse events. (TIFF)

S13 Fig Random-effects meta-analysis of remdesivir versus control interventions (standard care or placebo) on receipt of mechanical ventilation. (TIFF)

S14 Fig Trial sequential analysis of remdesivir versus control interventions (standard care or placebo) on receipt of mechanical ventilation. (TIFF)

S15 Fig Fixed-effect meta-analysis of remdesivir versus control interventions (standard care or placebo) on non-serious adverse events. (TIFF)

S16 Fig Trial sequential analysis of remdesivir versus control interventions (standard care or placebo) on non-serious adverse events. (TIFF)

S17 Fig Fixed-effect meta-analysis of hydroxychloroquine versus control interventions (standard care or placebo) on all-cause mortality. (PDF)

S18 Fig Trial sequential analysis of hydroxychloroquine versus control interventions (standard care or placebo) on all-cause mortality. (PDF)

S19 Fig Fixed-effect meta-analysis of hydroxychloroquine versus control interventions (standard care or placebo) on serious adverse events. (PDF)

S20 Fig Trial sequential analysis of hydroxychloroquine versus control interventions (standard care or placebo) on serious adverse events. (PDF)

S21 Fig Fixed-effect meta-analysis of hydroxychloroquine versus control interventions (standard care or placebo) on admission to intensive care. (PDF)

S22 Fig Trial sequential analysis of hydroxychloroquine versus control interventions (standard care or placebo) on admission to intensive care. (PDF)

S23 Fig Fixed-effect meta-analysis of hydroxychloroquine versus control interventions (standard care or placebo) on receipt of mechanical ventilation. (PDF)

S24 Fig Trial sequential analysis of hydroxychloroquine versus control interventions (standard care or placebo) on receipt of mechanical ventilation. (PDF)

S25 Fig Random-effects meta-analysis of hydroxychloroquine versus control interventions (standard care or placebo) on non-serious adverse events. (PDF)

S26 Fig Fixed-effect meta-analysis of lopinavir-ritonavir versus control interventions (standard care or placebo) on all-cause mortality. (TIFF)

S27 Fig Trial sequential analysis of lopinavir-ritonavir versus control interventions (standard care or placebo) on all-cause mortality. (TIFF)

S28 Fig Random-effects meta-analysis of lopinavir-ritonavir versus control interventions (standard care or placebo) on serious adverse events. (TIFF)

S29 Fig Trial sequential analysis of lopinavir-ritonavir versus control interventions (standard care or placebo) on serious adverse events. (TIFF)

S30 Fig Random-effects meta-analysis of lopinavir-ritonavir versus control interventions (standard care or placebo) on receipt of mechanical ventilation. (TIFF)

S31 Fig Trial sequential analysis of lopinavir-ritonavir versus control interventions (standard care or placebo) on receipt of mechanical ventilation. (TIFF)

S32 Fig Random-effects meta-analysis of lopinavir-ritonavir versus control interventions (standard care or placebo) on receipt of renal replacement therapy. (TIFF)

S33 Fig Trial sequential analysis of lopinavir-ritonavir versus control interventions on receipt of renal replacement therapy. (TIFF)

S34 Fig Fixed-effect meta-analysis of lopinavir-ritonavir versus control interventions (standard care or placebo) on non-serious adverse events. (TIFF)

S35 Fig Random-effects meta-analysis of interferon β-1a versus control interventions (standard care or placebo) on all-cause mortality. (TIFF)

S36 Fig Random-effects meta-analysis of interferon β-1a versus control interventions (standard care or placebo) on serious adverse events. (TIFF)

S37 Fig Random-effects meta-analysis of convalescent plasma versus control interventions (standard care or placebo) on all-cause mortality. (PDF)

S38 Fig Fixed-effect meta-analysis of azithromycin versus control interventions (standard care or placebo) on all-cause mortality. (PDF)

S39 Fig Trial sequential analysis of azithromycin versus control interventions (standard care or placebo) on all-cause mortality. (PDF)

S40 Fig Random-effects meta-analysis of azithromycin versus control interventions (standard care or placebo) on serious adverse events. (PDF)

S41 Fig Fixed-effect meta-analysis of azithromycin versus control interventions (standard care or placebo) on receipt of mechanical ventilation. (PDF)

S42 Fig Fixed-effect meta-analysis of azithromycin versus control interventions (standard care or placebo) on non-serious adverse events. (PDF)

S43 Fig Trial sequential analysis of azithromycin versus control interventions (standard care or placebo) on non-serious adverse events. (PDF)

S44 Fig Fixed-effect meta-analysis of colchicine versus control interventions (standard care or placebo) on all-cause mortality. (PDF)

S45 Fig Random-effects meta-analysis of colchicine versus control interventions (standard care or placebo) on non-serious adverse events. (PDF)

S46 Fig Fixed-effect meta-analysis of intravenous immunoglobin versus control interventions (standard care or placebo) on all-cause mortality. (PDF)

S47 Fig Trial sequential analysis of intravenous immunoglobin versus control interventions (standard care or placebo) on all-cause mortality. (PDF)

S48 Fig Fixed-effect meta-analysis of intravenous immunoglobin versus control interventions on serious adverse events. (PDF)

S49 Fig Trial sequential analysis of intravenous immunoglobin versus control interventions (standard care or placebo) on all-cause mortality. (PDF)

S50 Fig Random-effects meta-analysis of tocilizumab versus control interventions (standard care or placebo) on all-cause mortality. (PDF)

S51 Fig Trial sequential analysis of tocilizumab versus control interventions (standard care or placebo) on all-cause mortality. (PDF)

S52 Fig Random-effects meta-analysis of tocilizumab versus control interventions (standard care, placebo, or a co-intervention alone) on serious adverse events. (PDF)

S53 Fig Fixed-effect meta-analysis of tocilizumab versus control interventions (standard care, placebo, or a co-intervention alone) on serious adverse events. (TIFF)

S54 Fig Random-effects meta-analysis of tocilizumab versus control interventions (standard care or placebo) on admission to intensive care. (TIFF)

S55 Fig Random-effects meta-analysis of tocilizumab versus control interventions (standard care or placebo) on mechanical ventilation. (TIFF)

S56 Fig Trial sequential analysis of tocilizumab versus control interventions (standard care or placebo) on mechanical ventilation. (TIFF)

S57 Fig Fixed-effect meta-analysis of tocilizumab versus control interventions (standard care, placebo, or a co-intervention alone) on non-serious adverse events. (TIFF)

S58 Fig Random-effects meta-analysis of bromhexine versus control interventions (standard care) on all-cause mortality. (TIFF)

S59 Fig Random-effects meta-analysis of bromhexine versus control interventions (standard care) on non-serious adverse events. (TIFF)

S60 Fig Fixed-effect meta-analysis of tocilizumab versus control interventions (standard care, placebo, or a co-intervention alone) on non-serious adverse events. (TIFF)

S61 Fig Random-effects meta-analysis of bromhexine versus control interventions (standard care) on all-cause mortality. (PDF)

S62 Fig Random-effects meta-analysis of bromhexine versus control interventions (standard care) on non-serious adverse events. (PDF)

## References

[1] Guan W, Ni Z-y, Hu Y, Liang W-h, Ou C-q, He J-x, et al. Clinical characteristics of coronavirus disease 2019 in China. N Engl J Med. 2020;382(18):1708–20. 10.1056/NEJMoa2002032 32109013 PMC7092819

[2] World Health Organization. Novel Coronavirus (2019-nCOV). Situation Report 51. 2020 [Available from: https://www.who.int/docs/default-source/coronaviruse/situation-reports/20200311-sitrep-51-covid-19.pdf?sfvrsn=1ba62e57_10]

[3] Cao B, Wang Y, Wen D, Liu W, Wang J, Fan G, et al. A trial of lopinavir–ritonavir in adults hospitalized with severe Covid-19. N Engl J Med. 2020;382:1787–99. 10.1056/NEJMoa2001282 32187464 PMC7121492

[4] Huang C, Wang Y, Li X, Ren L, Zhao J, Hu Y, et al. Clinical features of patients infected with 2019 novel coronavirus in Wuhan, China. Lancet. 2020;395(10223):497–506. 10.1016/S0140-6736(20)30183-5 31986264 PMC7159299

[5] Chan JF, Yuan S, Kok K-H, To KK-W, Chu H, Yang J, et al. A familial cluster of pneumonia associated with the 2019 novel coronavirus indicating person-to-person transmission: a study of a family cluster. Lancet. 2020;395(10223):514–23. 10.1016/S0140-6736(20)30154-9 31986261 PMC7159286

[6] Xu Z, Shi L, Wang Y, Zhang J, Huang L, Zhang C, et al. Pathological findings of COVID-19 associated with acute respiratory distress syndrome. Lancet Respir Med. 2020;8(4):420–2. 10.1016/S2213-2600(20)30076-X 32085846 PMC7164771

[7] Fauci AS, Lane HC, Redfield RR. Covid-19—navigating the uncharted. N Engl J Med. 2020;382:1268–69 10.1056/NEJMe2002387 32109011 PMC7121221

[8] Juul S, Nielsen N, Bentzer P, Veroniki AA, Thabane L, Linder A, et al. Interventions for treatment of COVID-19: a protocol for a living systematic review with network meta-analysis including individual patient data (The LIVING Project). Syst Rev. 2020;9(1):108. 10.1186/s13643-020-01371-0 32386514 PMC7210799

[9] Moher D, Liberati A, Tetzlaff J, Altman DG, Group P. Preferred reporting items for systematic reviews and meta-analyses: the PRISMA statement. PLOS Med, 2009, 6(7), 10.1371/journal.pmed.1000097 PMC2707599 19621072

[10] Liberati A, Altman DG, Tetzlaff J, Mulrow C, Gøtzsche PC, Ioannidis JP, et al. The PRISMA statement for reporting systematic reviews and meta-analyses of studies that evaluate health care interventions: explanation and elaboration. BMJ. 2009;339:b2700 10.1136/bmj.b2700 19622552 PMC2714672

[11] Higgins J, Thomas J, Chandler J, Cumpston M, Li T, Page M, et al. Cochrane Handbook for Systematic Reviews of Interventions version 6.0 (updated July 2019). Cochrane, 2019. Available at www.training.cochrane.org/handbook. [Accessed November 4, 2020]

[12] The Cochrane Collaboration. Cochrane COVID-19 Study Register. Available at: https://covid-19.cochrane.org/: [Accessed November 2, 2020]

[13] Thorlund K, Dron L, Park J, Hsu G, Forrest JI, Mills EJ. A real-time dashboard of clinical trials for COVID-19. Lancet Dig Health. 2020; 2(6):e286–87 10.1016/S2589-7500(20)30086-8 32363333 PMC7195288

[14] Sterne JA, Savović J, Page MJ, Elbers RG, Blencowe NS, Boutron I, et al. RoB 2: a revised tool for assessing risk of bias in randomised trials. BMJ. 2019;366. 10.1136/bmj.l4898 31462531

[15] International Conference on Harmonisation of Technical Requirements for Registration of Pharmaceuticals for Human Use. ICH Harmonised Guideline: Integrated Addendum to ICH E6(R1): Guideline for Good Clinical Practice (ICH-GCP). 2015. Available from: https://ichgcp.net/ [Accessed November 4, 2020]

[16] Keus F, Wetterslev J, Gluud C, van Laarhoven CJ. Evidence at a glance: error matrix approach for overviewing available evidence. BMC Med Res Methodol. 2010;10(1):90. 20920306 10.1186/1471-2288-10-90 PMC2959031

[17] Jakobsen JC, Wetterslev J, Winkel P, Lange T, Gluud C. Thresholds for statistical and clinical significance in systematic reviews with meta-analytic methods. BMC Med Res Methodol. 2014;14(1):120. 10.1186/1471-2288-14-120 25416419 PMC4251848

[18] Review Manager (RevMan). Version 5.4. Copenhagen: The Nordic Cochrane Centre, The Cochrane Collaboration; 2020. Available at: https://training.cochrane.org/online-learning/core-software-cochrane-reviews/revman [Accessed November 10, 2020]

[19] StataCorp. Stata Statistical Software: Release 16. College Station, TX: StataCorp LLC.2019. Available at: www.stata.com [Accessed November 10, 2020]

[20] Higgins J, Green S. Cochrane Handbook for Systematic Reviews of Interventions. Version 5.1.0 [updated March 2011]. The Cochrane Collaboration. 2011. Available from: https://handbook-5-1.cochrane.org/ [Accessed November 4, 2020]

[21] Higgins JP, Spiegelhalter DJ. Being sceptical about meta-analyses: a Bayesian perspective on magnesium trials in myocardial infarction. Int J Epidemiol. 2002;31(1):96–104. 10.1093/ije/31.1.96 11914302

[22] Copenhagen Trial Unit. TSA—Trial Sequential Analysis. Available at: http://www.ctu.dk/tsa/ [Accessed November 2, 2020]

[23] Wetterslev J, Thorlund K, Brok J, Gluud C. Trial sequential analysis may establish when firm evidence is reached in cumulative meta-analysis. J Clin Epidemiol. 2008;61(1):64–75. 10.1016/j.jclinepi.2007.03.013 18083463

[24] Brok J, Thorlund K, Gluud C, Wetterslev J. Trial sequential analysis reveals insufficient information size and potentially false positive results in many meta-analyses. J Clin Epidemiol. 2008;61(8):763–9. 10.1016/j.jclinepi.2007.10.007 18411040

[25] Brok J, Thorlund K, Wetterslev J, Gluud C. Apparently conclusive meta-analyses may be inconclusive—trial sequential analysis adjustment of random error risk due to repetitive testing of accumulating data in apparently conclusive neonatal meta-analyses. Int J Epidemiol. 2008;38(1):287–98. 10.1093/ije/dyn188 18824466

[26] Thorlund K, Devereaux P, Wetterslev J, Guyatt G, Ioannidis JP, Thabane L, et al. Can trial sequential monitoring boundaries reduce spurious inferences from meta-analyses? Int J Epidemiol. 2008;38(1):276–86 10.1093/ije/dyn179 18824467

[27] Wetterslev J, Thorlund K, Brok J, Gluud C. Estimating required information size by quantifying diversity in random-effects model meta-analyses. BMC Med Res Methodol. 2009;9(1):86. 10.1186/1471-2288-9-86 20042080 PMC2809074

[28] Thorlund K, Engstrøm J, Wetterslev J, Brok J, Imberger G, Gluud C. User manual for trial sequential analysis (TSA). 2011. Available at: http://wwwctudk/tsa/files/tsa_manualpdf [Accessed July 4, 2020]

[29] Thorlund K, Anema A, Mills E. Interpreting meta-analysis according to the adequacy of sample size. An example using isoniazid chemoprophylaxis for tuberculosis in purified protein derivative negative HIV-infected individuals. Clin Epidemiol. 2010;2:57. 10.2147/clep.s9242 20865104 PMC2943189

[30] Imberger G, Thorlund K, Gluud C, Wetterslev J. False-positive findings in Cochrane meta-analyses with and without application of trial sequential analysis: an empirical review. BMJ Open. 2016;6(8):e011890. 10.1136/bmjopen-2016-011890 27519923 PMC4985805

[31] Beigel JH, Tomashek KM, Dodd LE, Mehta AK, Zingman BS, Kalil AC, et al. Remdesivir for the treatment of Covid-19—Preliminary report. N Engl J Med. 2020. 10.1056/NEJMoa2007764 [Epub ahead of print] 32649078

[32] Chen C, Zhang Y, Huang J, Yin P, Cheng Z, Wu J, et al. Favipiravir versus arbidol for COVID-19: A randomized clinical trial. medRxiv. 2020:2020.03.17.20037432. [Preprint]

[33] Chen J, Liu D, Liu L, Liu P, Xu Q, Xia L, et al. A pilot study of hydroxychloroquine in treatment of patients with common coronavirus disease-19 (COVID-19). J Zhejiang Univ (Med Sci). 2020;49(1) 10.3785/j.issn.1008-9292.2020.03.03 PMC8800713 32391667

[34] Chen Z, Hu J, Zhang Z, Jiang S, Han S, Yan D, et al. Efficacy of hydroxychloroquine in patients with COVID-19: results of a randomized clinical trial. medRxiv. 2020:2020.03.22.20040758. [Preprint] 10.1001/jama.2020.22240 33165621 PMC7653542

[35] Davoudi-Monfared E, Rahmani H, Khalili H, Hajiabdolbaghi M, Salehi M, Abbasian L, et al. Efficacy and safety of interferon beta-1a in treatment of severe COVID-19: a randomized clinical trial. medRxiv. 2020. 10.1128/AAC.01061-20 [Preprint] 32661006 PMC7449227

[36] Goldman JD, Lye DCB, Hui DS, Marks KM, Bruno R, Montejano R, et al. Remdesivir for 5 or 10 days in patients with severe Covid-19. N Engl J Med. 2020. 10.1056/NEJMoa2015301 [Epub ahead of print] 32459919 PMC7377062

[37] Hung IF-N, Lung K-C, Tso EY-K, Liu R, Chung TW-H, Chu M-Y, et al. Triple combination of interferon beta-1b, lopinavir–ritonavir, and ribavirin in the treatment of patients admitted to hospital with COVID-19: an open-label, randomised, phase 2 trial. Lancet. 2020. 395, 1695–704 10.1016/S0140-6736(20)31042-4 32401715 PMC7211500

[38] Li L, Zhang W, Hu Y, Tong X, Zheng S, Yang J, et al. Effect of convalescent plasma therapy on time to clinical improvement in patients with severe and life-threatening COVID-19: a randomized clinical trial. JAMA. 2020; 324(5):1–11 10.1001/jama.2020.10044 32492084 PMC7270883

[39] Li Y, Xie Z, Lin W, et al. Efficacy and safety of lopinavir/ritonavir or arbidol in adult patients with mild/moderate COVID-19: an exploratory randomized controlled trial. Cell Press. 2020 [Pre-proof] 10.1016/j.medj.2020.04.001 PMC7235585 32838353

[40] Lou Y, Liu L, Yao H, Hu X, Su J, Xu K, et al. Clinical outcomes and plasma concentrations of baloxavir marboxil and favipiravir in COVID-19 patients: an exploratory randomized, controlled trial. medRxiv. 2020. [Preprint] 10.1016/j.ejps.2020.105631 33115675 PMC7585719

[41] Tang W, Cao Z, Han M, et al. Hydroxychloroquine in patients with mainly mild to moderate coronavirus disease 2019: open label, randomised controlled trial. BMJ. 2020;369:1849. 10.1136/bmj.m1849 32409561 PMC7221473

[42] Wang Y, Zhang D, Du G, Du R, Zhao J, Jin Y, et al. Remdesivir in adults with severe COVID-19: a randomised, double-blind, placebo-controlled, multicentre trial. Lancet. 2020;395(10236):1569–78 10.1016/S0140-6736(20)31022-9 32423584 PMC7190303

[43] Wu CN, Xia LZ, Li KH, Ma WH, Yu DN, Qu B, et al. High-flow nasal-oxygenation-assisted fibreoptic tracheal intubation in critically ill patients with COVID-19 pneumonia: a prospective randomised controlled trial. Br J Anaesth. 2020;125(1):e166–68 10.1016/j.bja.2020.02.020 32200994 PMC7269901

[44] Zheng F, Zhou Y, Zhou Z, Ye F, Huang B, Huang Y, et al. A novel protein drug, novaferon, as the potential antiviral drug for COVID-19. medRxiv. 2020. 10.1101/2020.04.24.20077735 [Preprint]

[45] Zhong M, Sun A, Xiao T, Yao G, Sang L, Zheng X, et al. A randomized, single-blind, group sequential, active-controlled study to evaluate the clinical efficacy and safety of α-Lipoic acid for critically ill patients with coronavirus disease 2019 (COVID-19). medRxiv. 2020. 2020.04.15.20066266. [Preprint] 10.3389/fmed.2021.566609 PMC8854372 35186959

[46] Horby P, Lim WS, Emberson J, et al. Effect of dexamethasone in hospitalized patients with COVID-19 –preliminary report. medRxiv. 2020. 10.1101/2020.06.22.20137273 [Preprint]

[47] Chen L, Zhang Z-Y, Fu J-G, et al. Efficacy and safety of chloroquine or hydroxychloroquine in moderate type of COVID-19: a prospective open-label randomized controlled study. medRxiv. 2020. 10.1101/2020.06.19.20136093 [Preprint]

[48] Deftereos SG, Giannopoulos G, Vrachatis DA, et al. Effect of colchicine vs standard care on cardiac and inflammatory biomarkers and clinical outcomes in patients hospitalized with coronavirus disease 2019: the GRECCO-19 randomized clinical trial. JAMA Netw Open. 2020;3(6):e2013136. 10.1001/jamanetworkopen.2020.13136 32579195 PMC7315286

[49] Borba MGS, Val FFA, Sampaio VS, et al. Effect of high vs low doses of chloroquine diphosphate as adjunctive therapy for patients hospitalized with severe acute respiratory syndrome coronavirus 2 (SARS-CoV-2) infection. JAMA Netw Open. 2020;3(4). 10.1001/jamanetworkopen.2020.8857 32330277 PMC12124691

[50] Gharbharan A, Jordans CCE, Geurtsvankessel C, et al. Convalescent plasma for COVID-19: a randomized clinical trial. medrRxiv. 2020: 10.1101/2020.07.01.20139857 [Preprint]

[51] RECOVERY Collaborative Group. Dexamethasone in hospitalized patients with COVID-19—preliminary report. N Engl J Med. 2020. 10.1056/NEJMoa2021436 [Epub ahead of print] 32678530 PMC7383595

[52] Skipper CP, Pastick KA, Engen NW, et al. Hydroxychloroquine in nonhospitalized adults with early COVID-19: a randomized trial. Ann Intern Med. 2020 [Epub ahead of print] 10.7326/M20-4207 32673060 PMC7384270

[53] Cavalcanti AB, Zampieri FG, Rosa RG, et al. Hydroxychloroquine with or without azithromycin in mild-to-moderate Covid-19. *N Engl J Med*. 2020. 10.1056/NEJMoa2019014 [Epub ahead of print] 32706953 PMC7397242

[54] Mitjà O, Corbacho-Monné M, Ubals M, et al. Hydroxychloroquine for early treatment of adults with mild covid-19: a randomized-controlled trial. Clin Infect Dis. 2020 [Epub ahead of print] 10.1093/cid/ciaa1009 32674126 PMC7454406

[55] Corral-Gudino L, Bahamonde A, Arnaiz delas Revillas F, et al. GLUCOCOVID: A controlled trial of methylprednisolone in adults hospitalized with COVID-19 pneumonia. medRxiv. 2020. 10.1101/2020.06.17.20133579 [Preprint]

[56] Sakoulas G, Geriak M, Kullar R, et al. Intravenous Immunoglobulin (IVIG) significantly reduces respiratory morbidity in COVID-19 pneumonia: a prospective randomized trial. medRxiv. 2020. 10.1101/2020.07.20.20157891. [Preprint]

[57] Horby P, Mafham M, Linsell L, et al. Effect of Hydroxychloroquine in Hospitalized Patients with COVID-19: preliminary results from a multi-centre, randomized, controlled trial. medRxiv. 2020. 10.1101/2020.07.15.20151852. [Preprint]

[58] Chen C-P, Lin Y-C, Chen T-C, et al. A Multicenter, randomized, open-label, controlled trial to evaluate the efficacy and tolerability of hydroxychloroquine and a retrospective study in adult patients with mild to moderate coronavirus disease 2019 (COVID-19). medRxiv. 2020. 10.1371/journal.pone.0242763 [Preprint] 33264337 PMC7710068

[59] Chen J, Xia L, Liu L, et al. Antiviral activity and safety of darunavir/cobicistat for the treatment of COVID-19. Open Forum Infect Dis. 2020;7(7): ofaa241–ofaa. 10.1093/ofid/ofaa241 32671131 PMC7337805

[60] Chen Y-K, Huang Y-Q, Tang S-Q, et al. Comparative effectiveness and safety of ribavirin plus interferon-alpha, lopinavir/ritonavir plus interferon-alpha and ribavirin plus lopinavir/ritonavir plus interferon-alpha in patients with mild to moderate novel coronavirus pneumonia: results of a randomized, open-labeled prospective study. SSRN. 2020. 10.2139/ssrn.3576905. [Preprint]

[61] Guvenmez O, Keskin H, Ay B, et al. The comparison of the effectiveness of lincocin® and azitro® in the treatment of covid-19-associated pneumonia: A prospective study. J Popul Ther Clin Pharmacol. 2020;27(SP1):e5–e10. 32543164 10.15586/jptcp.v27iSP1.684

[62] Yuan X, Yi W, Liu B, et al. Pulmonary radiological change of COVID-19 patients with 99mTc-MDP treatment. medRxiv. 2020. 10.1101/2020.04.07.20054767. [Preprint]

[63] Idelsis E-M, Jesus P-E, Yaquelin D-R, et al. Effect and safety of combination of interferon alpha-2b and gamma or interferon alpha-2b for negativization of SARS-CoV-2 viral RNA. Preliminary results of a randomized controlled clinical trial. medRxiv. 2020. 10.1101/2020.07.29.20164251. [Preprint]

[64] Beigel JH, Tomashek KM, Dodd LE, et al. Remdesivir for the treatment of COVID-19—final report. N Eng J Med. 2020: 383:1813–26 10.1056/NEJMoa2007764 PMC7262788 32445440

[65] Duarte M, Pelorosso FG, Nicolosi L, et al. Telmisartan for treatment of Covid-19 patients: an open randomized clinical trial. Preliminary report. medRxiv. 2020:2020.08.04.20167205 [Preprint]

[66] Ivashchenko AA, Dmitriev KA, Vostokova NV, et al. AVIFAVIR for treatment of patients with moderate COVID-19: interim results of a phase II/III multicenter randomized clinical trial. medRxiv. 2020:2020.07.26.20154724 [Preprint] 10.1093/cid/ciaa1176 32770240 PMC7454388

[67] Jeronimo CMP, Farias MEL, Val FFA, et al. Methylprednisolone as adjunctive therapy for patients hospitalized with COVID-19 (METCOVID): a randomised, double-blind, phase IIb, placebo-controlled trial. Clin Infect Dis. 2020. 10.1093/cid/ciaa1177 [Epub ahead of print] 32785710 PMC7454320

[68] Mehboob R, Ahmad F, Qayyum A, et al. Aprepitant as a combinant with dexamethasone reduces the inflammation via neurokinin 1 receptor antagonism in severe to critical COVID-19 patients and potentiates respiratory recovery: a novel therapeutic approach. medRxiv. 2020:2020.08.01.20166678 [Preprint]

[69] Vlaar AP, de Bruin S, Busch M, et al. Anti-C5a Antibody (IFX-1) Treatment of severe COVID-19: An exploratory phase 2 randomized controlled trial. SSRN. 2020. 10.2139/ssrn.3658226 [Preprint] PMC7521913 33015643

[70] Duymaz T. Pulmonary rehabilitation in post-acute period of COVID-19 infection: prospective randomized controleld trial. SSRN. 2020. 10.2139/ssrn.3590506. [Preprint]

[71] Mansour E, Palma AC, Ulaf RG, et al. Pharmacological inhibition of the kinin-kallikrein system in severe COVID-19: a proof-of-concept study. medRxiv. 2020:2020.08.11.20167353 [Preprint]

[72] Ren Z, Luo H, Yu Z, et al. A randomized, open-label, controlled clinical trial of azvudine tablets in the treatment of mild and common COVID-19, a pilot study. Adv Sci. 2020;7(19) 10.1002/advs.202001435 PMC7404576 35403380

[73] Miller J, Bruen C, Schnaus M, et al. Auxora versus standard of care for the treatment of severe or critical COVID-19 pneumonia: results from a randomized controlled trial. Crit Care. 2020;24(1):502. 10.1186/s13054-020-03220-x 32795330 PMC7427272

[74] Shu L, Niu C, Li R, et al. Treatment of severe COVID-19 with human umbilical cord mesenchymal stem cells. Stem Cell Res Ther. 2020;11(1):361. 10.1186/s13287-020-01875-5 32811531 PMC7432540

[75] Zhang J, Rao X, Li Y, et al. High-dose vitamin C infusion for the treatment of critically ill COVID-19. Research Square. 2020. 10.21203/rs.3.rs-52778/v1 [Preprint]

[76] Abd-Elsalam S, Esmail ES, Khalaf M, et al. Hydroxychloroquine in the treatment of COVID-19: a multicenter randomized controlled study. Am J Trop Med Hyg. 2020;103(4):1635–9. 10.4269/ajtmh.20-0873 32828135 PMC7543820

[77] Avendano-Sola C, Ramos-Martinez A, Munez-Rubio E, et al. Convalescent plasma for COVID-19: A multicenter, randomized clinical trial. medRxiv. 2020:2020.08.26.20182444.

[78] Abbaspour Kasgari H, Moradi S, Shabani AM, et al. Evaluation of the efficacy of sofosbuvir plus daclatasvir in combination with ribavirin for hospitalized COVID-19 patients with moderate disease compared with standard care: a single-centre, randomized controlled trial. J Antimicrob Chemother. 2020. 10.1093/jac/dkaa332 [Epub ahead of print] 32812025 PMC7454669

[79] Sadeghi A, Ali Asgari A, Norouzi A, et al. Sofosbuvir and daclatasvir compared with standard of care in the treatment of patients admitted to hospital with moderate or severe coronavirus infection (COVID-19): a randomized controlled trial. J Antimicrob Chemother. 2020:75(11) 10.1093/jac/dkaa334 32812039 PMC7454592

[80] Rahmani H, Davoudi-Monfared E, Nourian A, et al. Interferon β-1b in treatment of severe COVID-19: a randomized clinical trial. Int Immunopharmacol. 2020;88:106903. 10.1016/j.intimp.2020.106903 32862111 PMC7445008

[81] Sekhavati E, Jafari F, SeyedAlinaghi S, et al. Safety and effectiveness of azithromycin in patients with COVID-19: an open-label randomised trial. Int J Antimicrob Agents. 2020:106143 10.1016/j.ijantimicag.2020.106143 32853672 PMC7445147

[82] Furtado RHM, Berwanger O, Fonseca HA, et al. Azithromycin in addition to standard of care versus standard of care alone in the treatment of patients admitted to the hospital with severe COVID-19 in Brazil (COALITION II): a randomised clinical trial. Lancet. 2020;396:959–67 10.1016/S0140-6736(20)31862-6 32896292 PMC7836431

[83] Castillo ME, Entrenas Costa LM, Vaquero Barrios JM, et al. Effect of calcifediol treatment and best available therapy versus best available therapy on intensive care unit admission and mortality among patients hospitalized for COVID-19: a pilot randomized clinical study. J Steroid Biochem Mol Biol. 2020. 10.1016/j.jsbmb.2020.105751):105751 [Epub ahead of print] PMC7456194 32871238

[84] Cheng L-l, Guan W-j, Duan C-y, et al. Effect of recombinant human granulocyte colony–stimulating factor for patients with coronavirus disease 2019 (COVID-19) and lymphopenia: a randomized clinical trial. JAMA Intern med. 2020. 10.1001/jamainternmed.2020.5503 [Epub ahead of print] 32910179 PMC7489414

[85] Spinner CD, Gottlieb RL, Criner GJ, et al. Effect of remdesivir vs standard care on clinical status at 11 days in patients with moderate COVID-19: a randomized clinical trial. JAMA. 2020;324(11):1048–57. 10.1001/jama.2020.16349 32821939 PMC7442954

[86] Tomazini BM, Maia IS, Cavalcanti AB, et al. Effect of dexamethasone on days alive and ventilator-free in patients with moderate or severe acute respiratory distress syndrome and COVID-19: the CoDEX randomized clinical trial. JAMA. 2020. 10.1001/jama.2020.17021 [Epub ahead of print] 32876695 PMC7489411

[87] Dequin P-F, Heming N, Meziani F, et al. Effect of hydrocortisone on 21-Day mortality or respiratory support among critically ill patients with COVID-19: a randomized clinical trial. JAMA. 2020;324(13);1298–1306 10.1001/jama.2020.16761 32876689 PMC7489432

[88] The Writing Committee for the REMAP-CAP Investigators. Effect of hydrocortisone on mortality and organ support in patients with severe COVID-19: the REMAP-CAP COVID-19 corticosteroid domain randomized clinical trial. JAMA. 2020;324(13);1317–29 10.1001/jama.2020.17022 32876697 PMC7489418

[89] Rosas I, Bräu N, Waters M, et al. Tocilizumab in hospitalized patients with COVID-19 pneumonia. medRxiv. 2020. 10.1101/2020.08.27.20183442 [Preprint]

[90] Agarwal A, Mukherjee A, Kumar G, et al. Convalescent plasma in the management of moderate COVID-19 in India: an open-label parallel-arm phase II multicentre randomized controlled trial (PLACID Trial). medRxiv. 2020. [Preprint] 10.1136/bmj.m3939 33093056 PMC7578662

[91] Lopes MIF, Bonjorno LP, Giannini MC, et al. Beneficial effects of colchicine for moderate to severe COVID-19: an interim analysis of a randomized, double-blinded, placebo controlled clinical trial. medRxiv. 2020. 10.1101/2020.08.06.20169573 [Preprint] PMC7868202 33542047

[92] Wang D, Fu B, Peng Z, et al. Tocilizumab ameliorates the hypoxia in COVID-19 moderate patients with bilateral pulmonary lesions: a randomized, controlled, open-label, multicenter trial. SSRN. 2020: [Preprint]

[93] Li T, Sun L, Zhang W, et al. Bromhexine hydrochloride tablets for the treatment of moderate COVID-19: an open-label randomized controlled pilot study. Clin Transl Sci. 2020. 10.1111/cts.12881 [Epub ahead of print] 32881359 PMC7719397

[94] Gharebaghi N, Nejadrahim R, Mousavi SJ, et al. The use of intravenous immunoglobulin gamma for the treatment of severe coronavirus disease 2019: a randomised placebo-controlled double-blind clinical trial. Research Square. 2020. 10.21203/rs.3.rs-40899/v2 [Preprint] PMC7576972 33087047

[95] Farahani RH, Mosaed R, Nezami-Asl A, et al. Evaluation of the efficacy of methylprednisolone pulse therapy in treatment of covid-19 adult patients with severe respiratory failure: randomized, clinical trial. Research Square. 2020. 10.21203/rs.3.rs-66909/v1 [Preprint]

[96] ClinicalTrials.gov National Library of Medicine (US). Identifier NCT04244591: Glucocorticoid therapy for COVID-19 critically ill patients with severe acute respiratory failure Available from: https://clinicaltrials.gov/ct2/show/NCT042445912020 [Accessed November 2, 2020)

[97] Edalatifard M, Akhtari M, Salehi M, et al. Intravenous methylprednisolone pulse as a treatment for hospitalised severe COVID-19 patients: results from a randomised controlled clinical trial. Eur Res J. 2020. 10.1183/13993003.02808-2020 [Epub ahead of print] 32943404 PMC7758541

[98] ClinicalTrials.gov National Library of Medicine (US). Identifier NCT04325061: Efficacy of dexamethasone treatment for patients with ARDS caused by COVID-19 (DEXA-COVID19) Available at: https://clinicaltrials.gov/ct2/show/NCT043250612020 [Accessed November 2, 2020]

[99] Delgado-Enciso I, Paz-Garcia J, Carlos E Barajas-Saucedo C. Patient-reported health outcomes after treatment of COVID-19 with nebulized and/or intravenous neutral electrolyzed saline combined with usual medical care versus usual medical care alone: a randomized, open-label, controlled trial. Research Square. [Preprint] 10.21203/rs.3.rs-68403/v1 34306189 PMC8281484

[100] Kimura KS, Freeman MH, Wessinger BC, et al., editors. Interim analysis of an open-label randomized controlled trial evaluating nasal irrigations in non-hospitalized patients with COVID-19. Int Forum Allergy Rhinol. 2020. 10.1002/alr.22703 [Epub ahead of print] PMC7722064 32914928

[101] Wu X, Yu K, Wang Y, et al. Efficacy and safety of triazavirin therapy for coronavirus disease 2019: a pilot randomized controlled trial. Engineering. 2020. [Epub ahead of print] 10.1016/j.eng.2020.08.011 32923016 PMC7476906

[102] de Alencar JCG, Moreira CdL, Müller AD, et al. Double-blind, randomized, placebo-controlled trial with N-acetylcysteine for treatment of severe acute respiratory syndrome caused by COVID-19. Clin Infect Dis. 2020. [Epub ahead of print] 10.1093/cid/ciaa1443 32964918 PMC7543361

[103] Ansarin K, Tolouian R, Ardalan M, et al. Effect of bromhexine on clinical outcomes and mortality in COVID-19 patients: a randomized clinical trial. BioImpacts. 2020;10(4):209–15. 10.34172/bi.2020.27 32983936 PMC7502909

[104] Lyngbakken MN, Berdal J-E, Eskesen A, et al. A pragmatic randomized controlled trial reports the efficacy of hydroxychloroquine on coronavirus disease 2019 viral kinetics. Research Square. 2020. 10.1038/s41467-020-19056-6 [Preprint] 33082342 PMC7576792

[105] Horby PW, Mafham M, Bell JL, et al. Lopinavir-ritonavir in patients admitted to hospital with COVID-19 (RECOVERY): a randomised, controlled, open-label, platform trial. Lancet. 2020. [Epub ahead of print] 10.1016/S0140-6736(20)32013-4 33031764 PMC7535623

[106] Salehzadeh F, Pourfarzi F, Ataei S. The impact of colchicine on the COVID-19 patients: a clinical trial study. Research Square. 2020. 10.21203/rs.3.rs-69374/v1 [Preprint] PMC9450200 36128202

[107] Ulrich RJ, Troxel AB, Carmody E, et al. Treating COVID-19 with hydroxychloroquine (TEACH): a multicenter, double-blind, randomized controlled trial in hospitalized patients. Open Forum Infect Dis. 2020. [Epub ahead of print] 10.1093/ofid/ofaa446 33134417 PMC7543602

[108] Nojomi M, Yasin Z, Keyvani H, et al. Effect of arbidol on COVID-19: a randomized controlled trial. Research Square. 2020. 10.1186/s12879-020-05698-w [Preprint] 33317461 PMC7734453

[109] Pan H, Peto R, Abdool Karim Q, et al. Repurposed antiviral drugs for COVID-19; interim WHO SOLIDARITY trial results. medRxiv. 2020. 10.1101/2020.10.15.20209817 [Preprint] PMC7727327 33264556

[110] Hermine O, Mariette X, Tharaux P-L, et al. Effect of tocilizumab vs usual care in adults hospitalized with COVID-19 and moderate or severe pneumonia: a randomized clinical trial. JAMA Intern Med. 2020. 10.1001/jamainternmed.2020.6820 [Epub ahead of print] 33080017 PMC7577198

[111] Salvarani C, Dolci G, Massari M, et al. Effect of tocilizumab vs standard care on clinical worsening in patients hospitalized with COVID-19 pneumonia: a randomized clinical trial. JAMA Intern Med. 2020. 10.1001/jamainternmed.2020.6615 [Epub ahead of print]. 33080005 PMC7577199

[112] Stone JH, Frigault MJ, Serling-Boyd NJ, et al. Efficacy of tocilizumab in patients hospitalized with COVID-19. N Eng J Med. 2020. 10.1056/NEJMoa2028836 [Epub ahead of print] 33085857 PMC7646626

[113] Zhao H, Zhu Q, Zhang C, et al. Tocilizumab combined with favipiravir in the treatment of COVID-19: A multicenter trial in a small sample size. Biomed Pharmacother. 2020. 10.1016/j.biopha.2020.110825 (Pre-proof). PMC7524677 33378989

[114] Cao Y, Wei J, Zou L, et al. Ruxolitinib in treatment of severe coronavirus disease 2019 (COVID-19): A multicenter, single-blind, randomized controlled trial. J Allergy Clin Immunol. 2020. 10.1016/j.jaci.2020.05.019 [Epub ahead of print] 32470486 PMC7250105

[115] Davoodi L, Abedi SM, Salehifar E, et al. Febuxostat therapy in outpatients with suspected COVID-19: A clinical trial. Int J Clin Pract. 2020:e13600. 10.1111/ijcp.13600 32603531 PMC7361151

[116] Sterne JA, Murthy S, Diaz JV, et al. Association between administration of systemic corticosteroids and mortality among critically ill patients with COVID-19: a meta-analysis. *JAMA*. 2020: 10.1001/jama.2020.17023 32876694 PMC7489434

[117] Siemieniuk RA, Bartoszko JJ, Ge L, et al. Drug treatments for covid-19: living systematic review and network meta-analysis. BMJ. 2020;370:m2980. 10.1136/bmj.m2980 32732190 PMC7390912

[118] Juul S, Nielsen EE, Feinberg J, et al. Interventions for treatment of COVID-19: A living systematic review with meta-analyses and trial sequential analyses (The LIVING Project). PLOS Med. 2020;17(9):e1003293. 10.1371/journal.pmed.1003293 32941437 PMC7498193

[119] U.S. Food and Drug Administration (FDA) News Release, FDA approves first treatment for COVID-19 [press release]. Available at: https://www.fda.gov/news-events/press-announcements/fda-approves-first-treatment-covid-19 [Accessed October 23, 2020]

[120] Axfors C, Schmitt AM, Janiaud P, et al. Mortality outcomes with hydroxychloroquine and chloroquine in COVID-19: an international collaborative meta-analysis of randomized trials. *medRxiv*. 2020. 10.1101/2020.09.16.20194571 [Preprint]. PMC8050319 33859192

[121] Garattini S, Jakobsen JC, Wetterslev J, et al. Evidence-based clinical practice: Overview of threats to the validity of evidence and how to minimise them. Eur J Intern Med. 2016;32:13–21. 10.1016/j.ejim.2016.03.020 27160381

[122] Gluud LL. Bias in clinical intervention research. Am J Epidemiol. 2006;163(6):493–501. 10.1093/aje/kwj069 16443796

[123] Kjaergard LL, Villumsen J, Gluud C. Reported methodologic quality and discrepancies between large and small randomized trials in meta-analyses. Ann Intern Med. 2001;135(11):982–9. 10.7326/0003-4819-135-11-200112040-00010 11730399

[124] Jüni P, Altman DG, Egger M. Assessing the quality of controlled clinical trials. BMJ. 2001;323(7303):42–6. 10.1136/bmj.323.7303.42 11440947 PMC1120670

[125] Moher D, Jones A, Cook DJ, et al. Does quality of reports of randomised trials affect estimates of intervention efficacy reported in meta-analyses? Lancet. 1998;352(9128):609–13. 10.1016/S0140-6736(98)01085-X 9746022

[126] Schulz KF, Chalmers I, Hayes RJ, et al. Empirical evidence of bias: dimensions of methodological quality associated with estimates of treatment effects in controlled trials. JAMA. 1995;273(5):408–12. 10.1001/jama.273.5.408 7823387

[127] Hrobjartsson A, Emanuelsson F, Skou Thomsen AS, et al. Bias due to lack of patient blinding in clinical trials. A systematic review of trials randomizing patients to blind and nonblind sub-studies. Int J Epidemiol. 2014;43(4):1272–83. 10.1093/ije/dyu115 24881045 PMC4258786

[128] Hrobjartsson A, Skou Thomsen AS, Emanuelsson F, et al. Observer bias in randomized clinical trials with measurement scale outcomes: a systematic review of trials with both blinded and nonblinded assessors. CMAJ. 2013;185(4). 10.1503/cmaj.120744 23359047 PMC3589328

[129] Hrobjartsson A, Thomsen AS, Emanuelsson F, et al. Observer bias in randomised clinical trials with binary outcomes: systematic review of trials with both blinded and non-blinded outcome assessors. BMJ. 2012;344:e1119. 10.1136/bmj.e1119 22371859

[130] The Cochrane Collaboration. Living mapping and living systematic review of Covid-19 studies. Available at: www.covid-nma.com [Accessed November 2, 2020]

