# Supplementary figures and images for: Interventions for treatment of COVID-19: Second edition of a living systematic review with meta-analyses and trial sequential analyses (The LIVING Project)
Source: PLoS One. 2021 Mar 11;16(3):e0248132. doi: 10.1371/journal.pone.0248132 (PMC7954033; doi:10.1371/journal.pone.0248132)

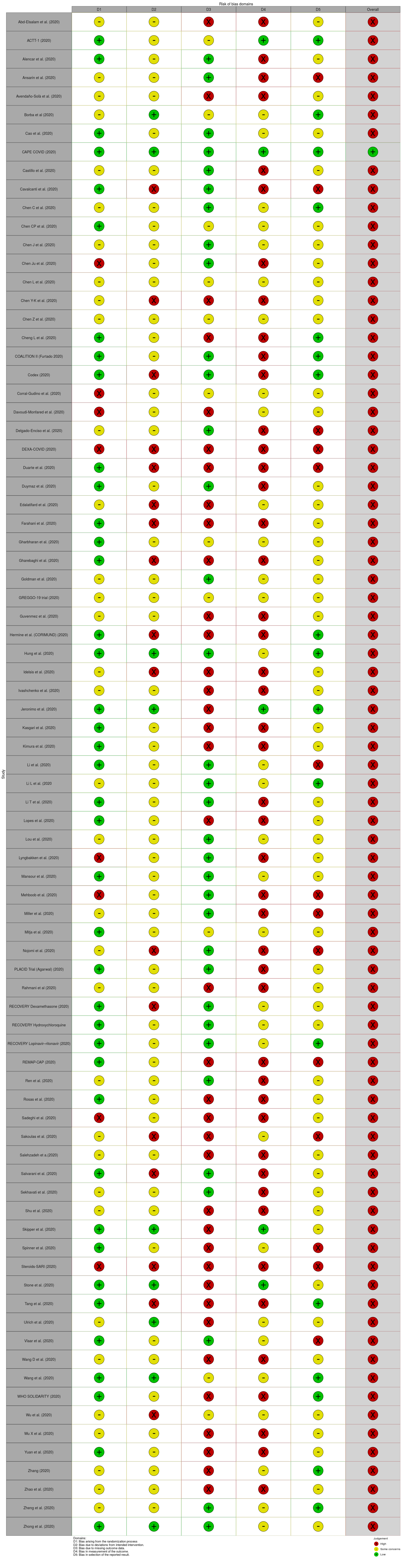

Supplement: S3 Table — (TIFF) [file pone.0248132.s005.tiff]

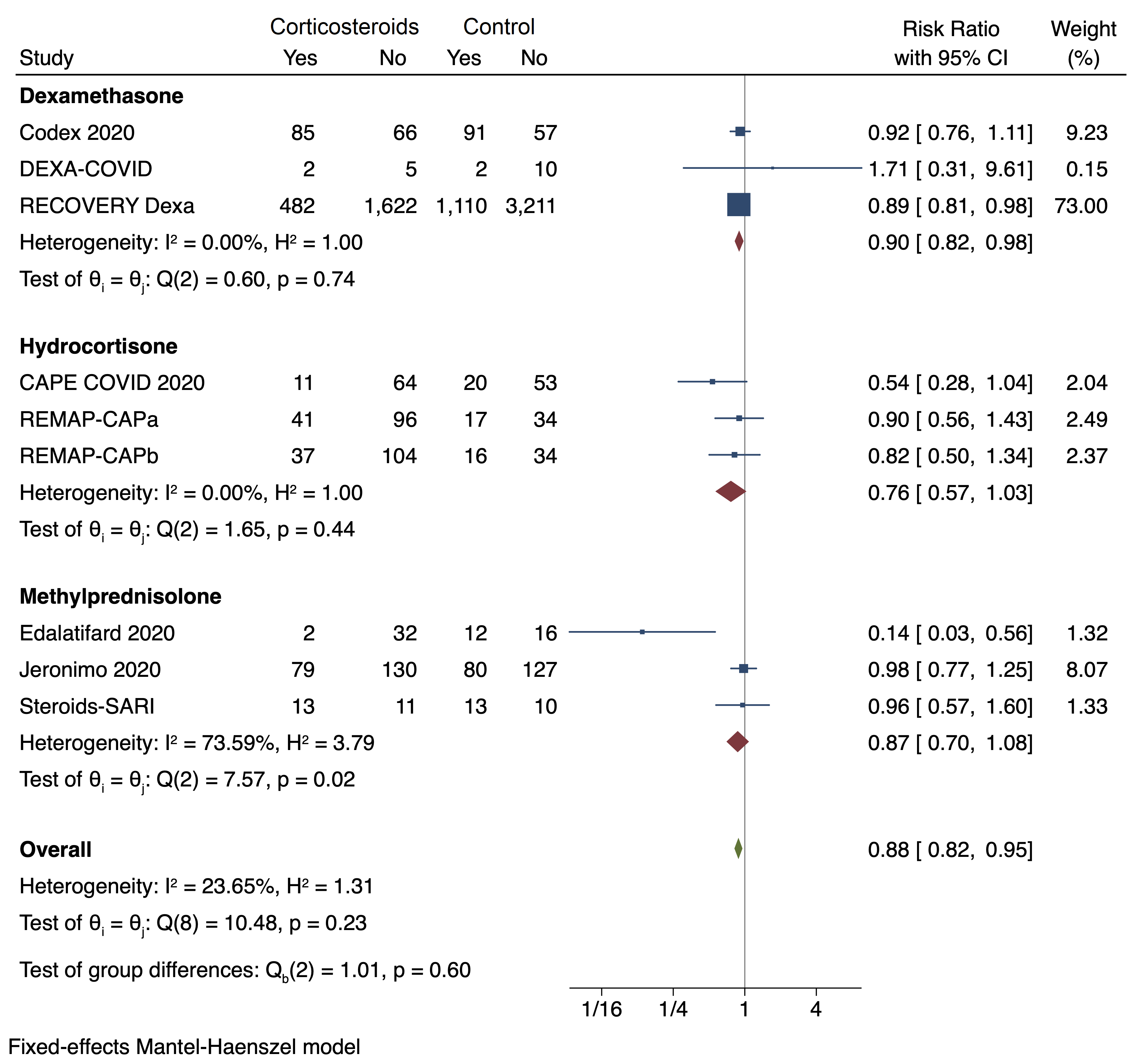

Supplement: S1 Fig — (TIFF) [file pone.0248132.s069.tiff]

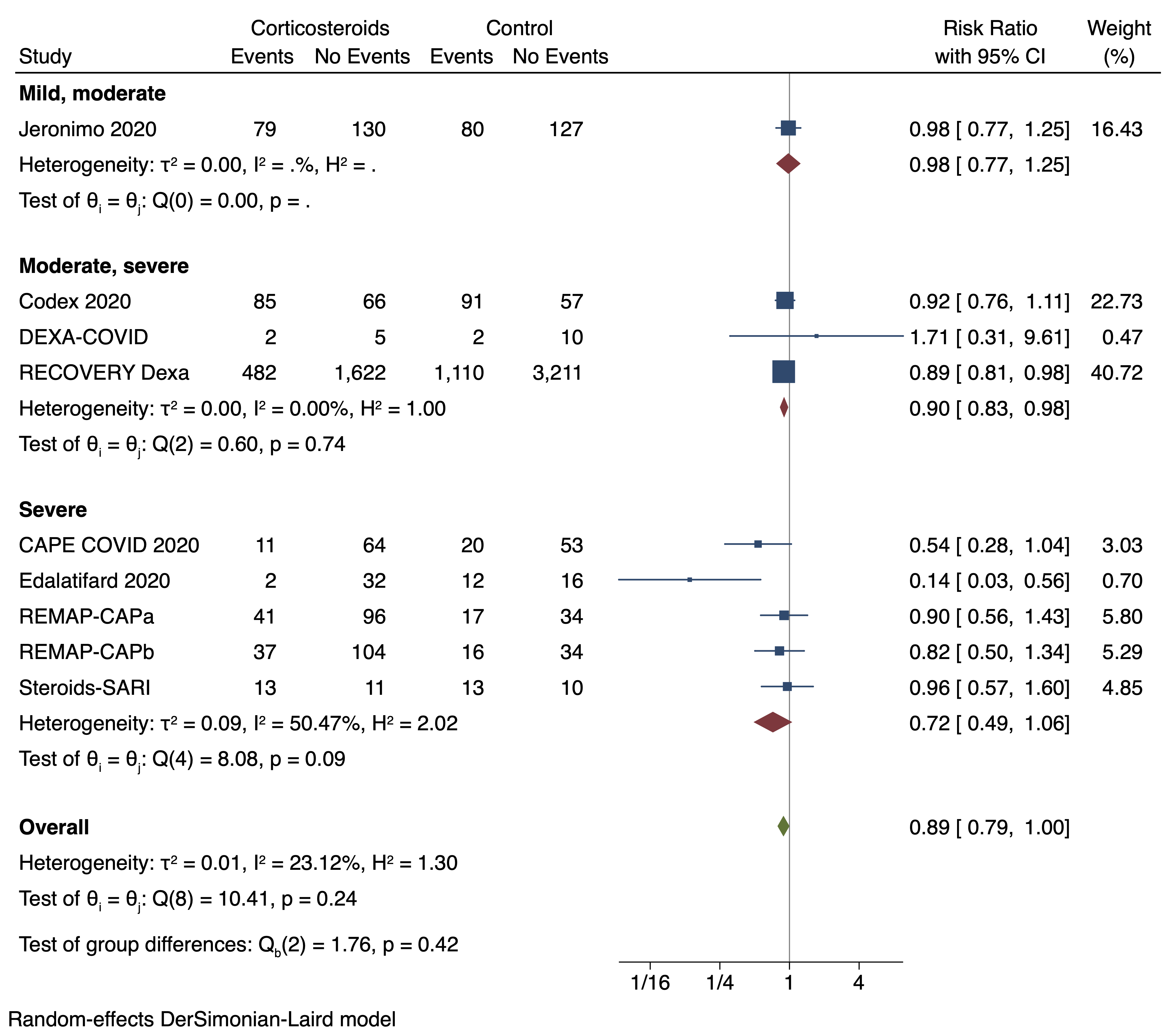

Supplement: S2 Fig — (TIFF) [file pone.0248132.s070.tiff]

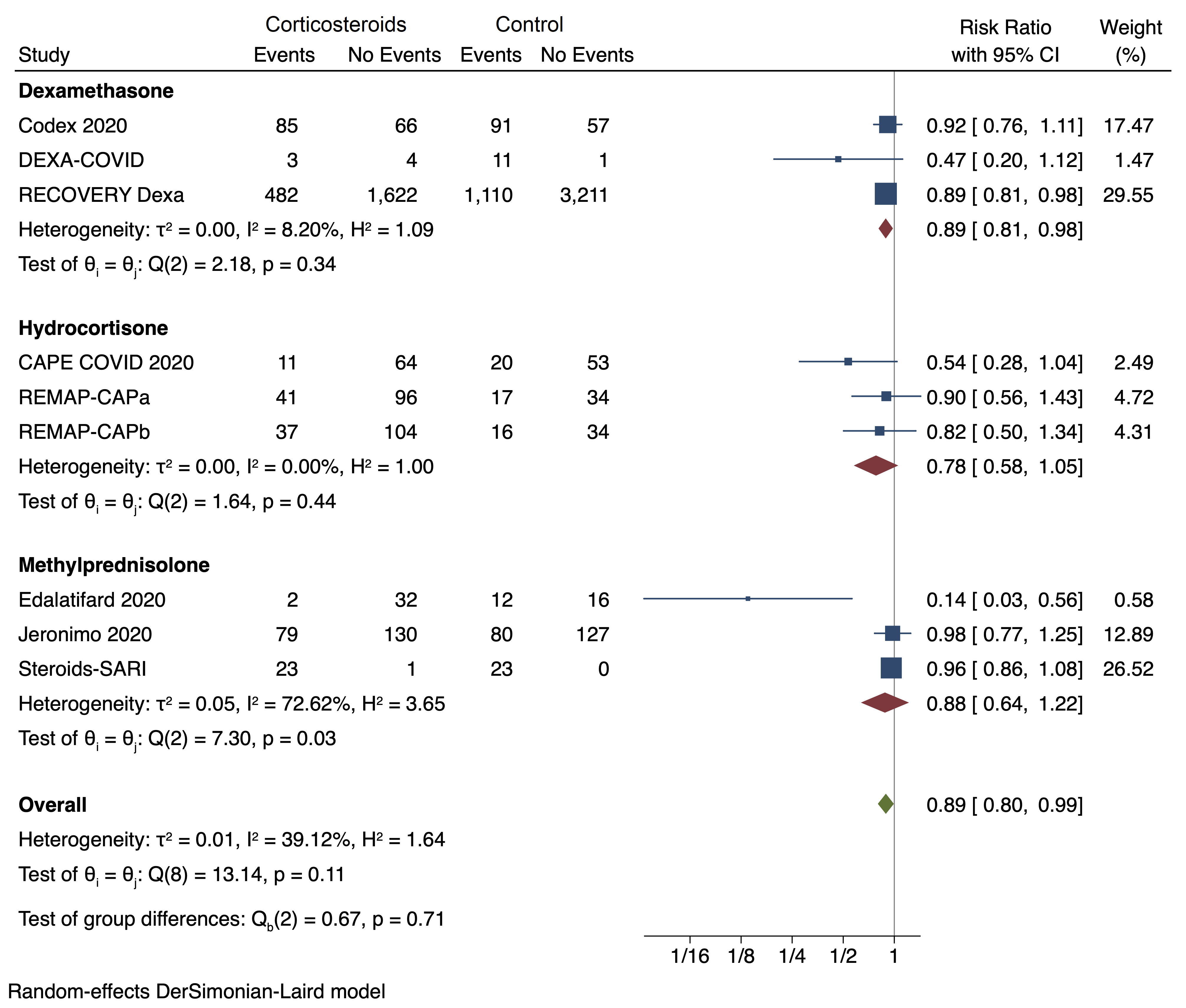

Supplement: S3 Fig — (TIFF) [file pone.0248132.s071.tiff]

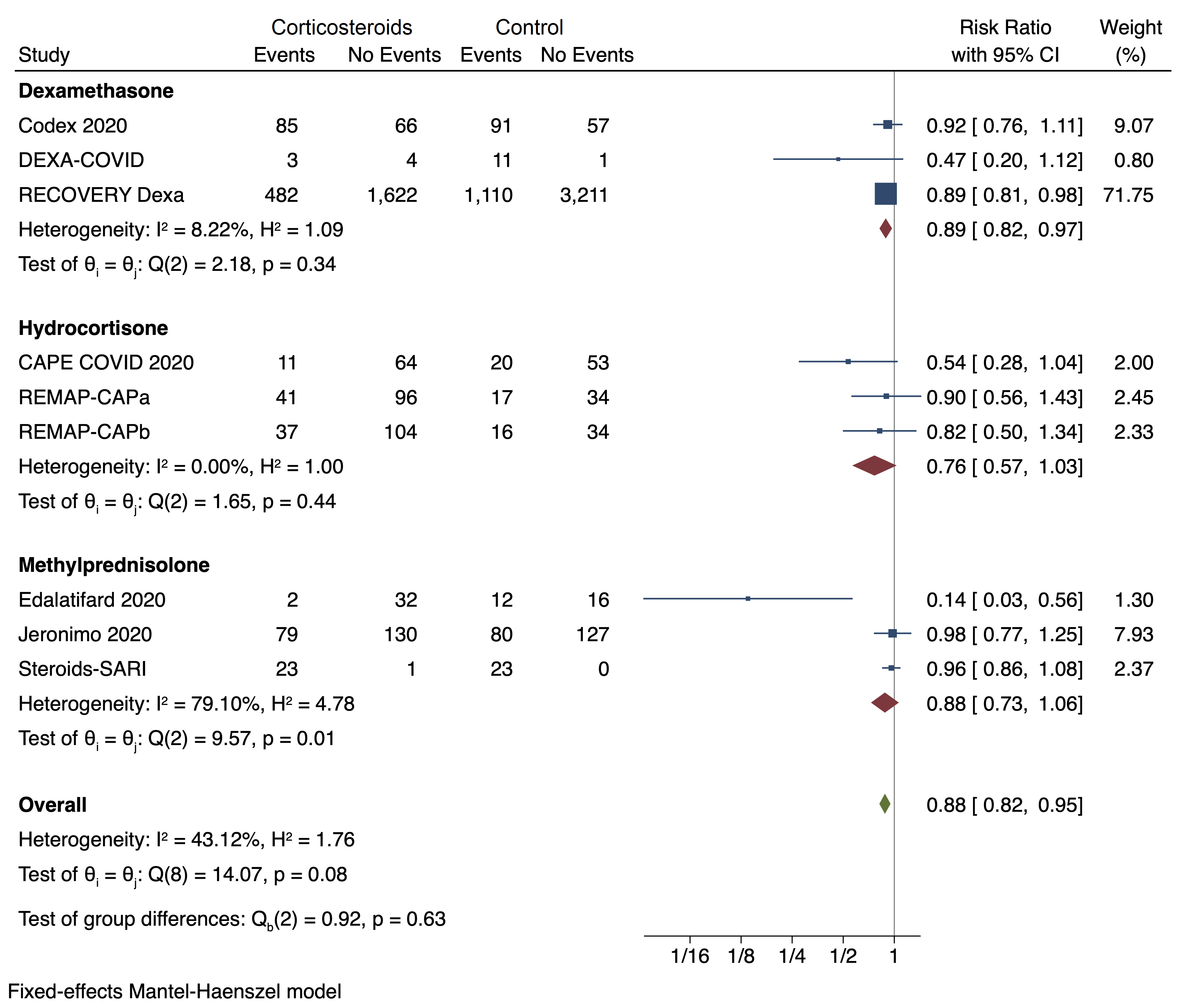

Supplement: S4 Fig — (TIFF) [file pone.0248132.s072.tiff]

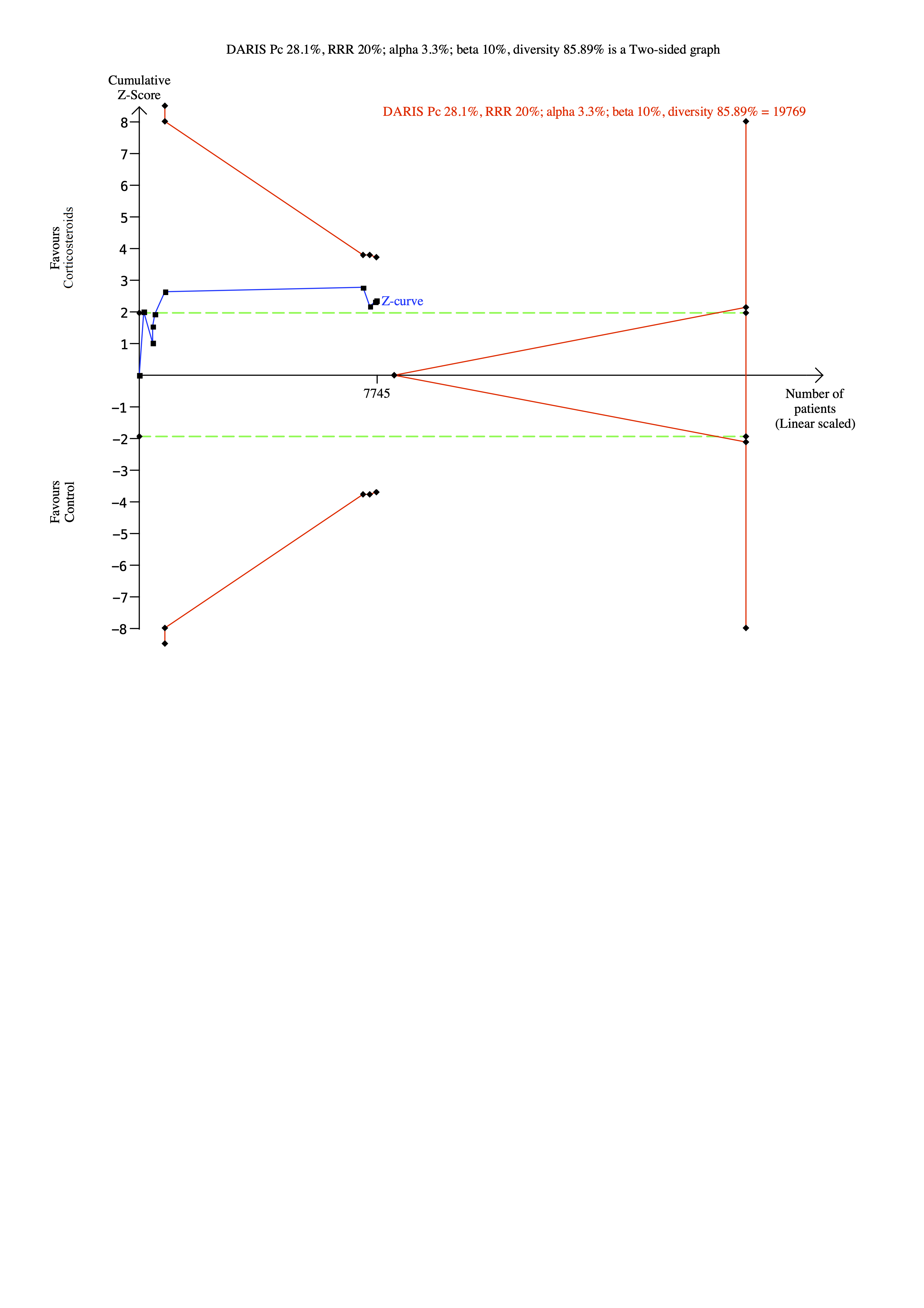

Supplement: S5 Fig — (TIFF) [file pone.0248132.s073.tiff]

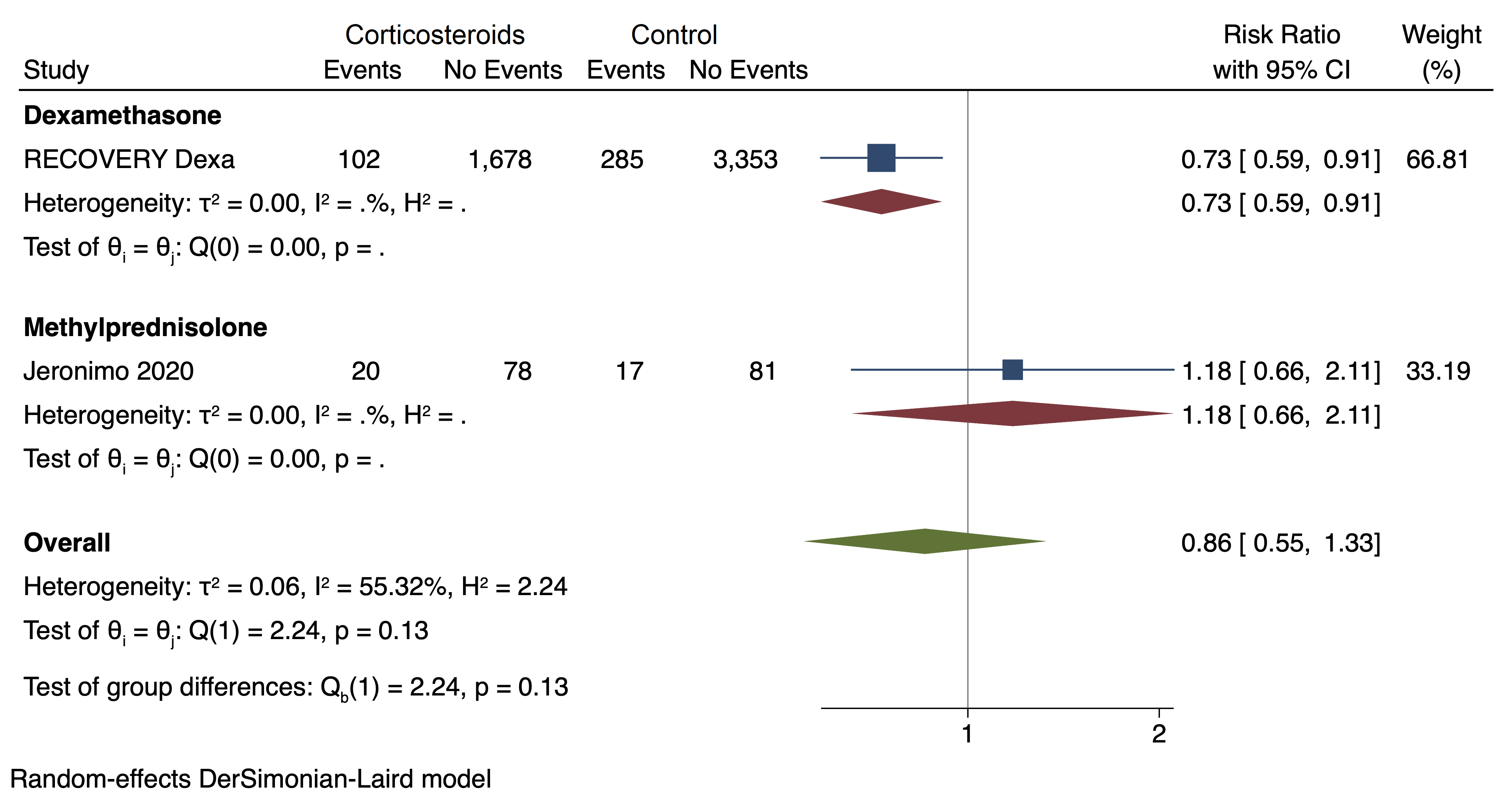

Supplement: S6 Fig — (TIFF) [file pone.0248132.s074.tiff]

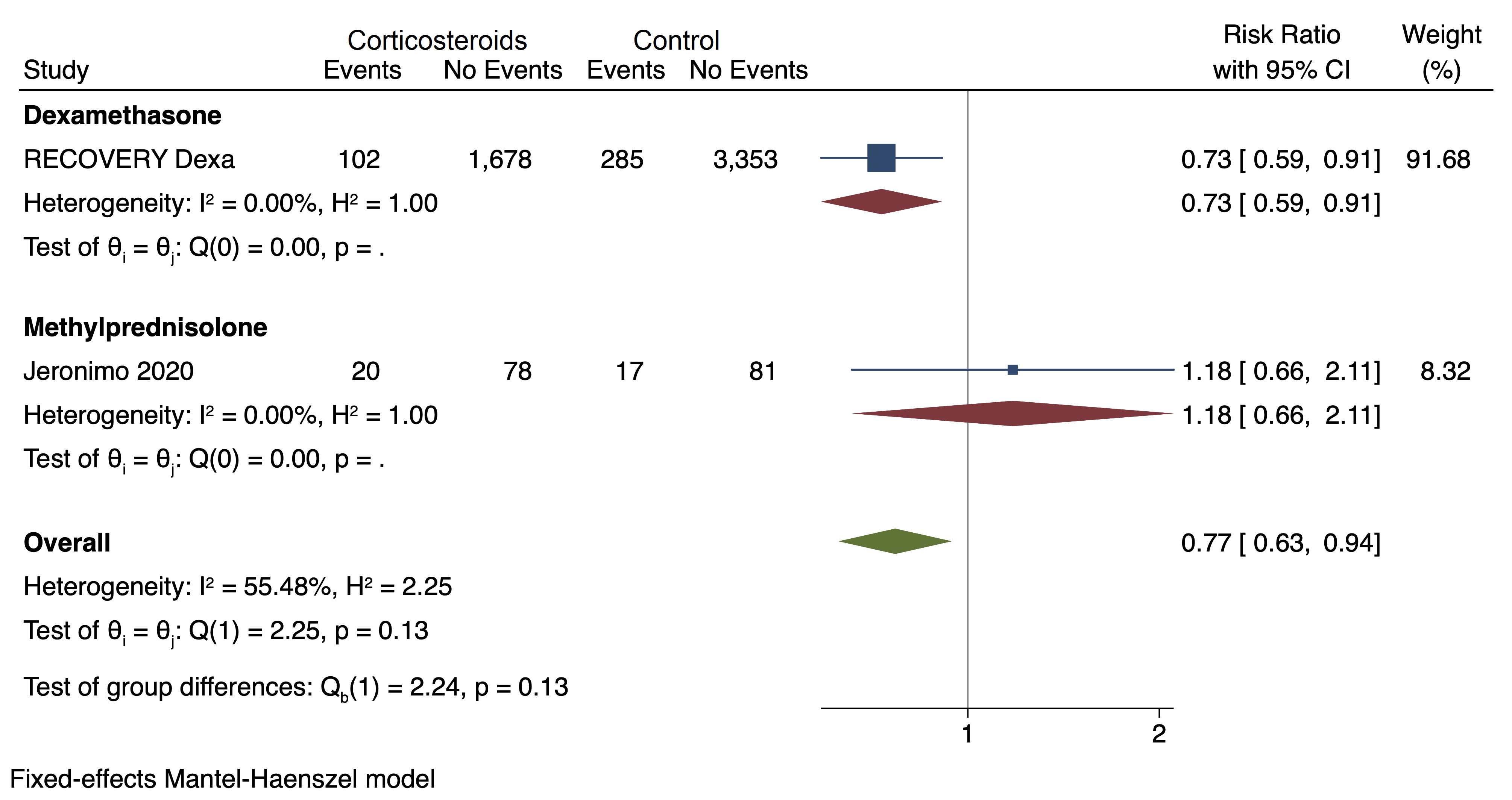

Supplement: S7 Fig — (TIFF) [file pone.0248132.s075.tiff]

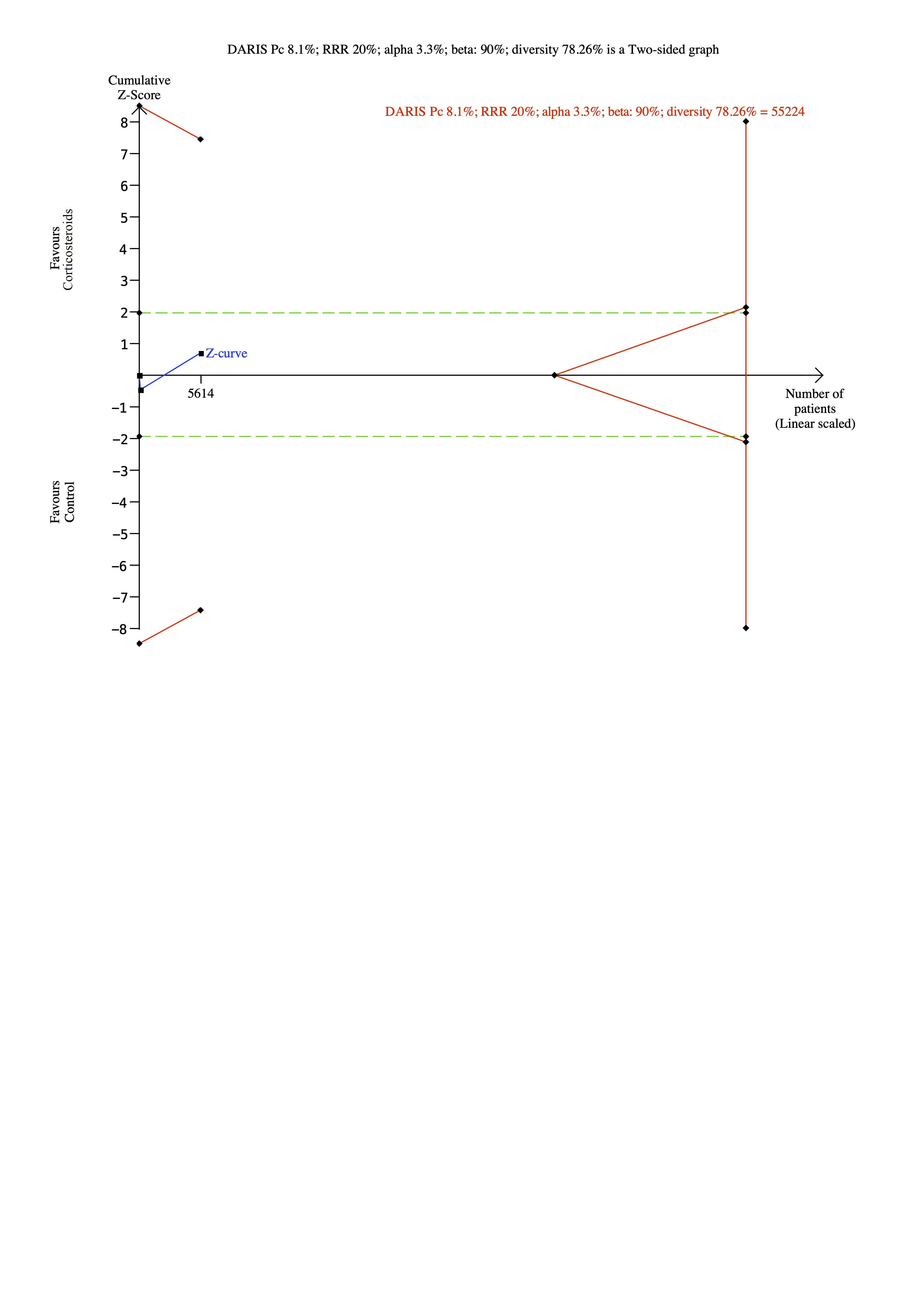

Supplement: S8 Fig — (TIFF) [file pone.0248132.s076.tiff]

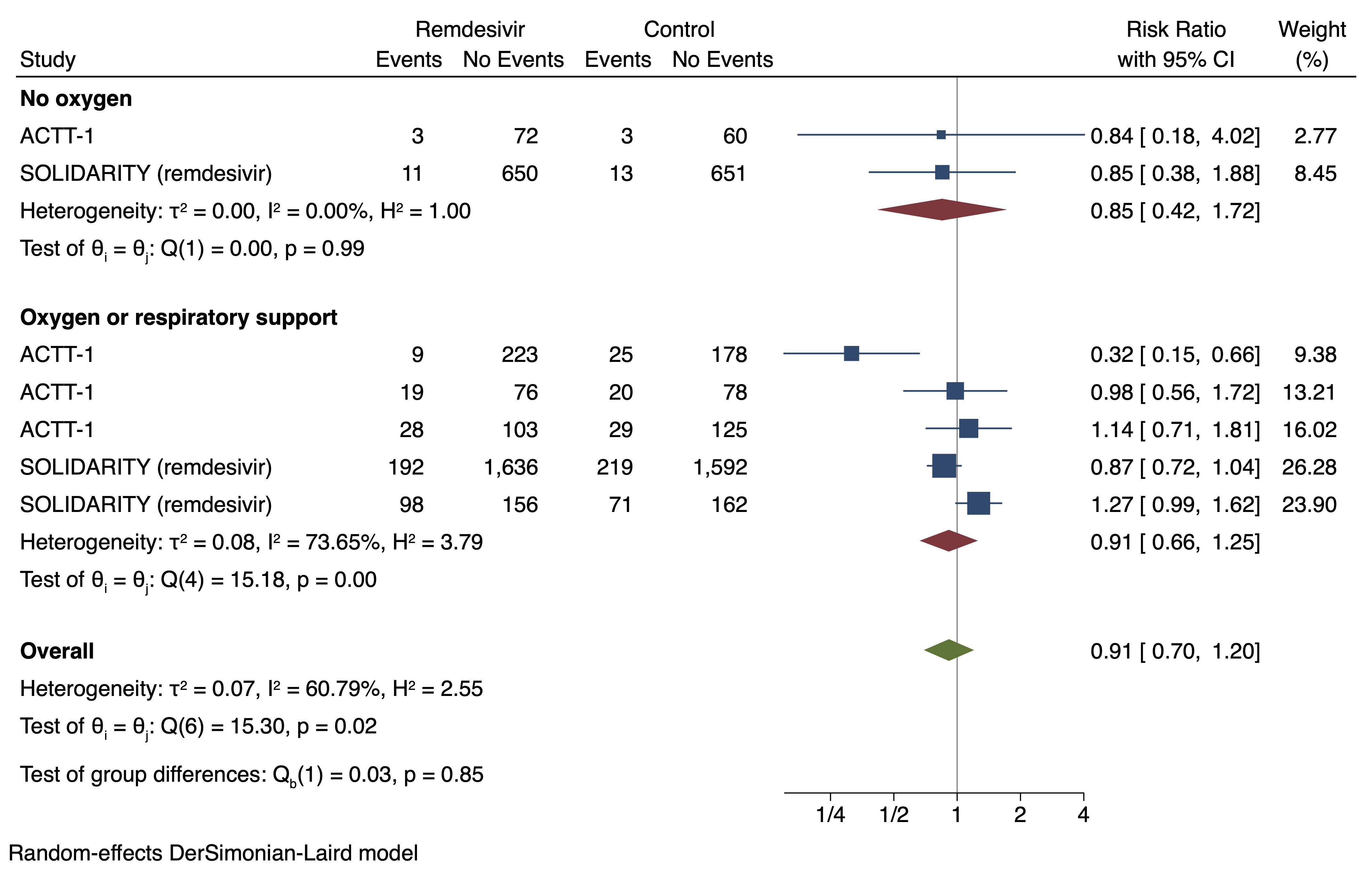

Supplement: S9 Fig — (TIFF) [file pone.0248132.s077.tiff]

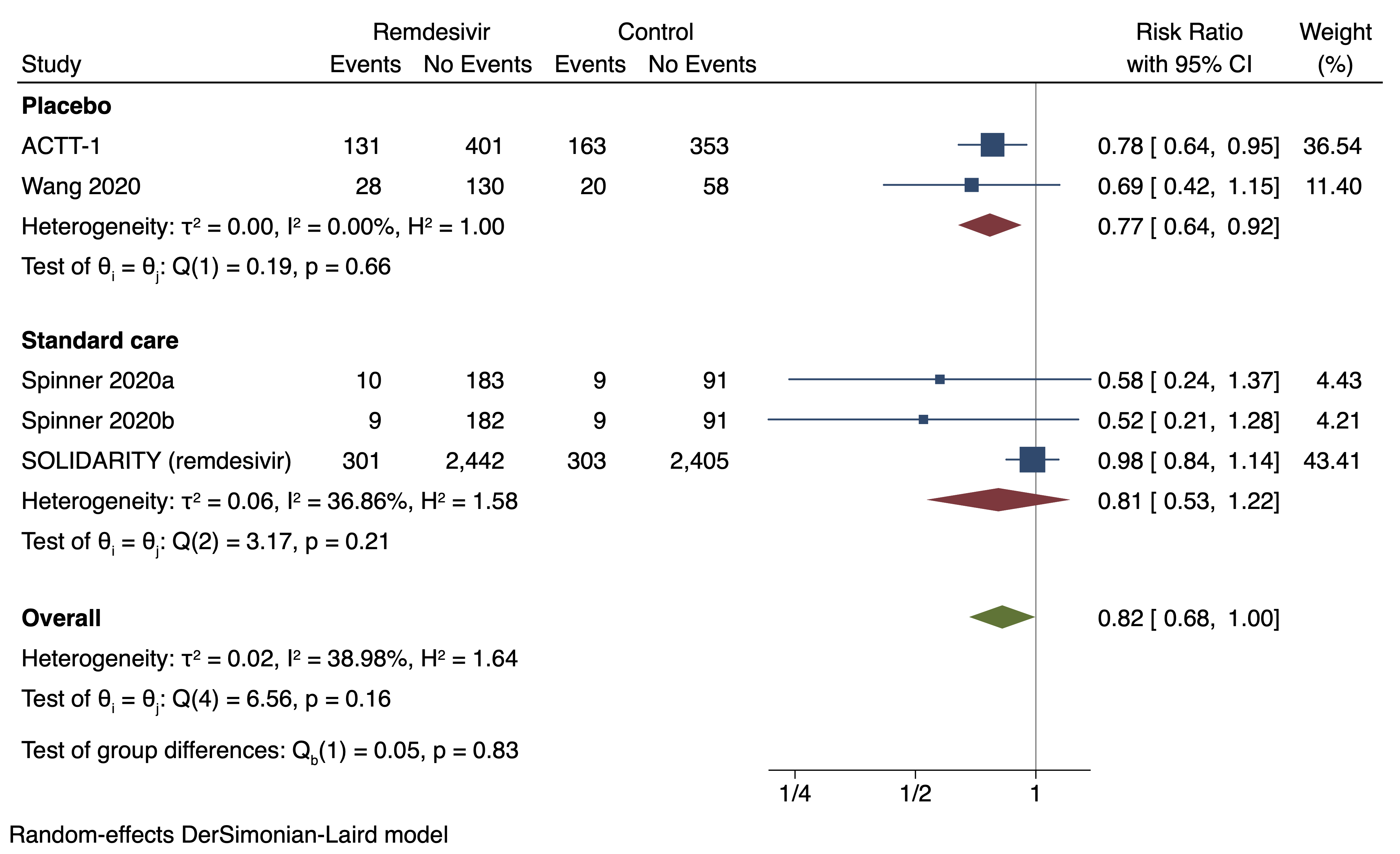

Supplement: S10 Fig — (TIFF) [file pone.0248132.s078.tiff]

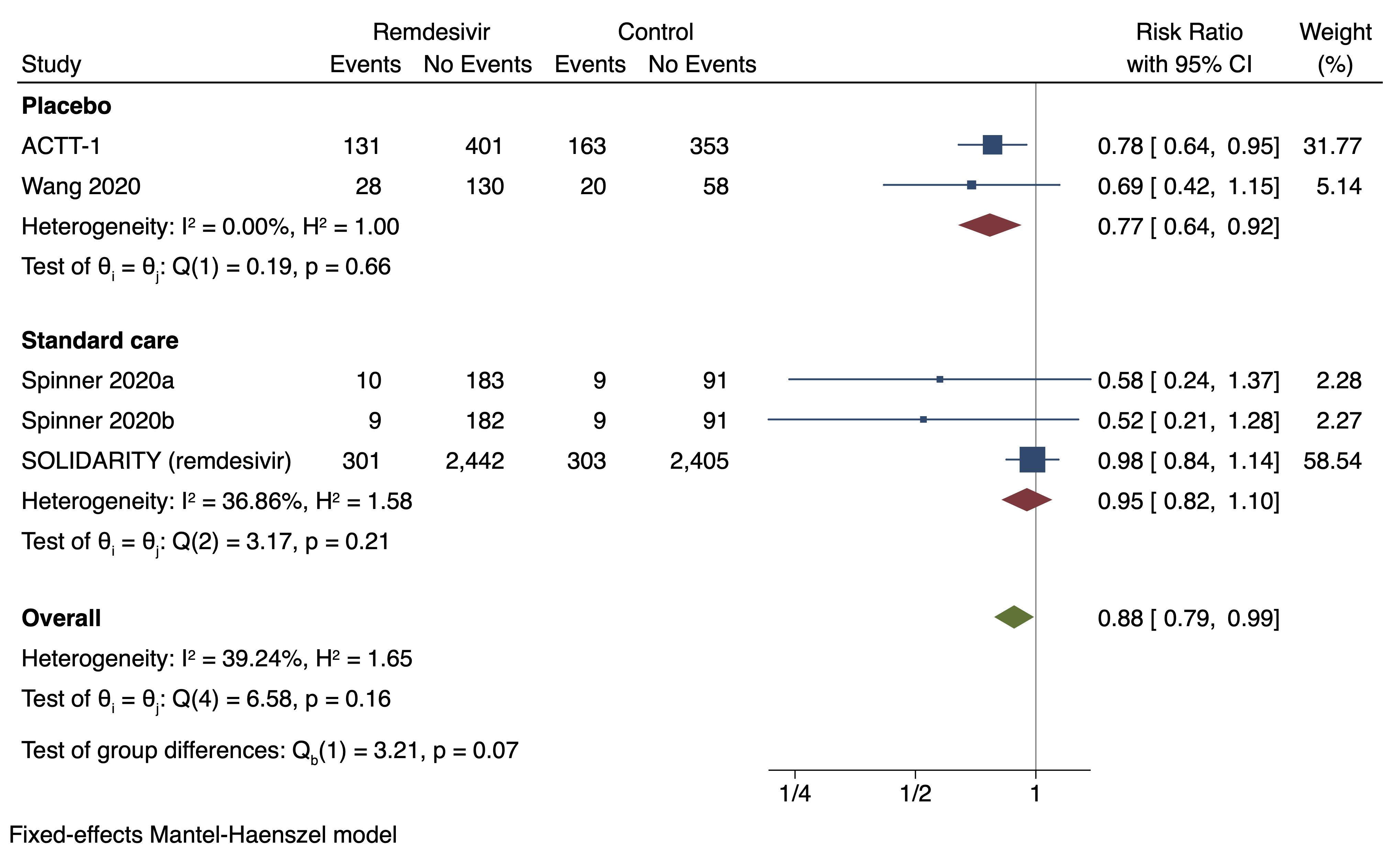

Supplement: S11 Fig — (TIFF) [file pone.0248132.s079.tiff]

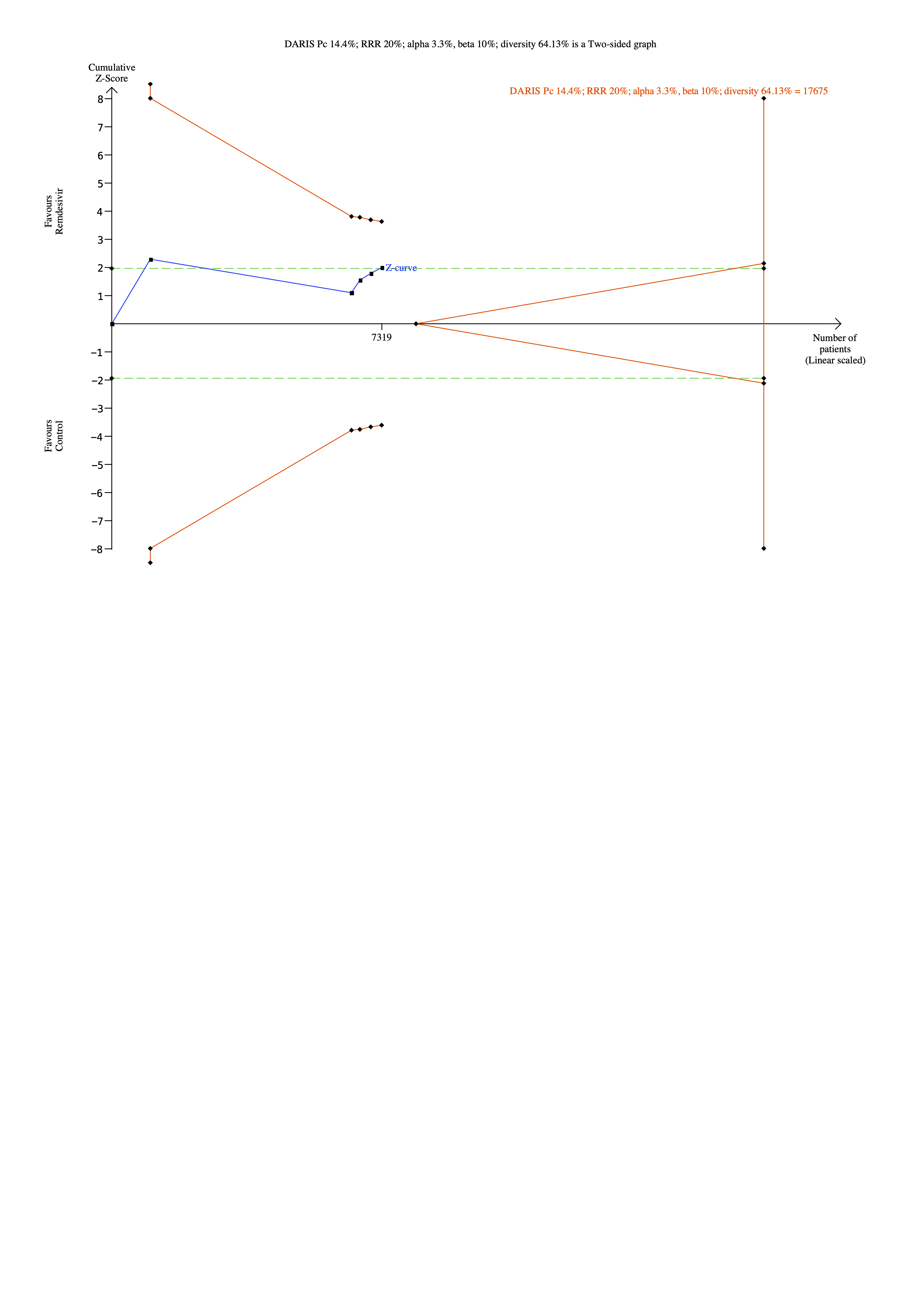

Supplement: S12 Fig — (TIFF) [file pone.0248132.s080.tiff]

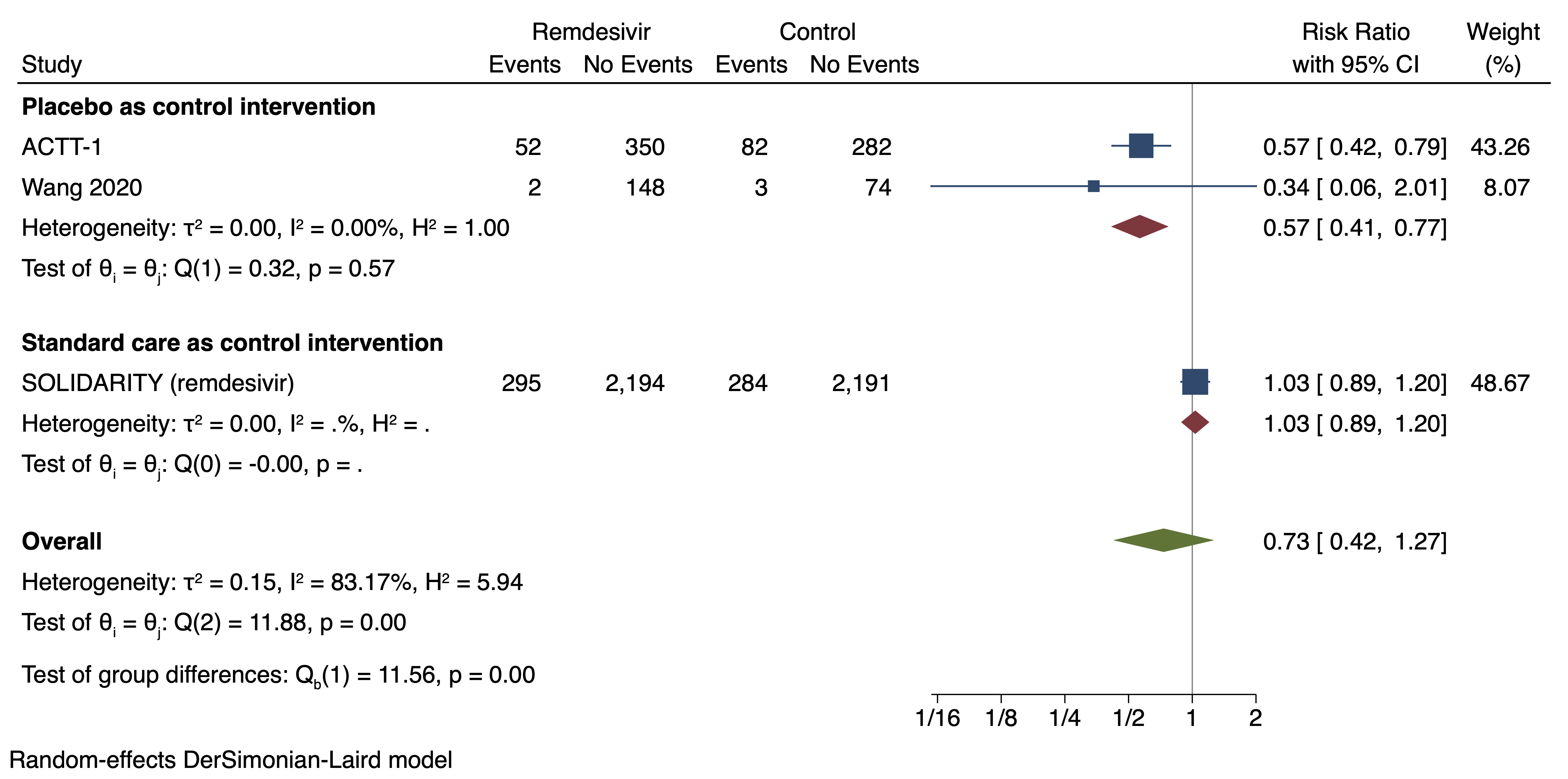

Supplement: S13 Fig — (TIFF) [file pone.0248132.s081.tiff]

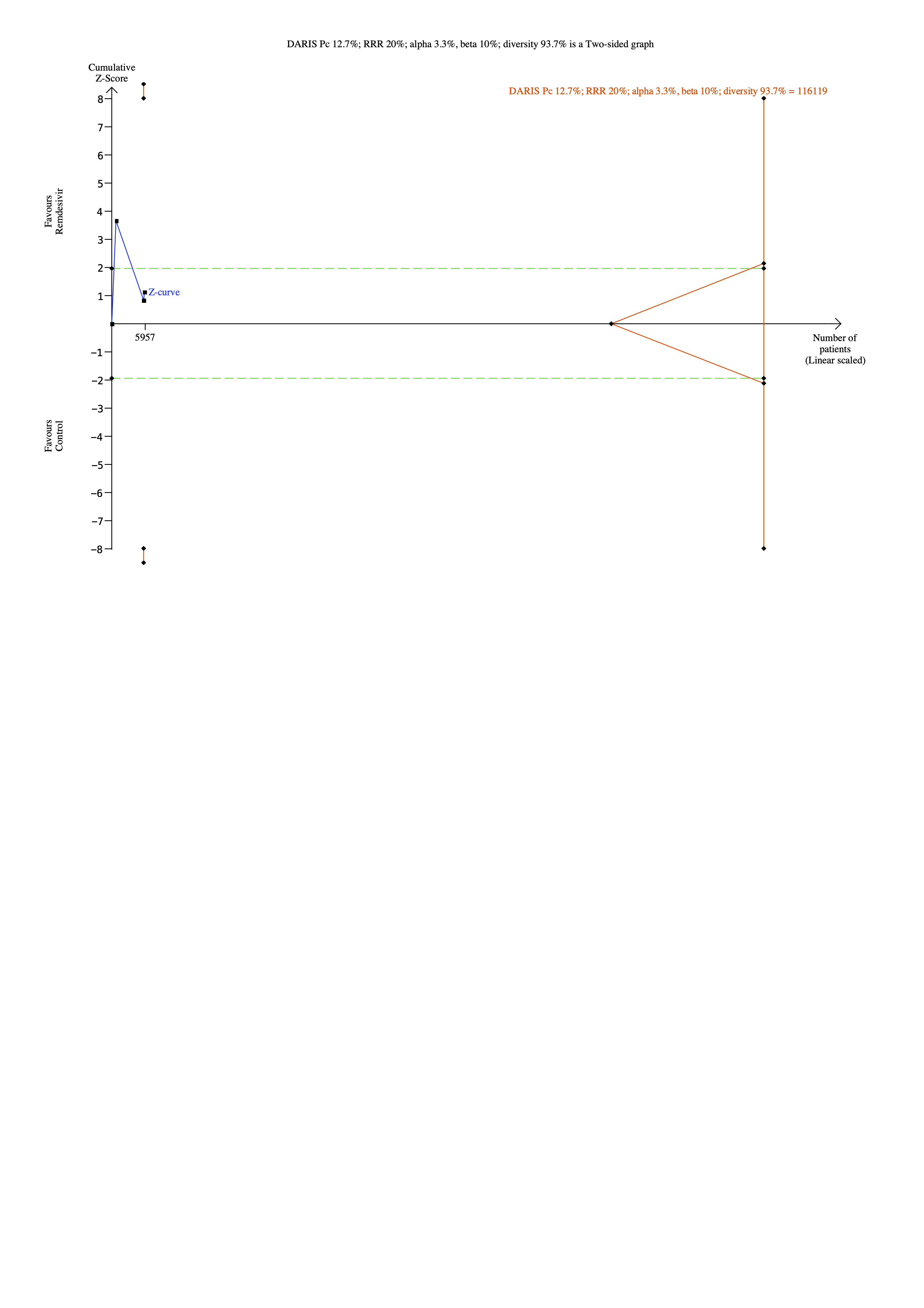

Supplement: S14 Fig — (TIFF) [file pone.0248132.s082.tiff]

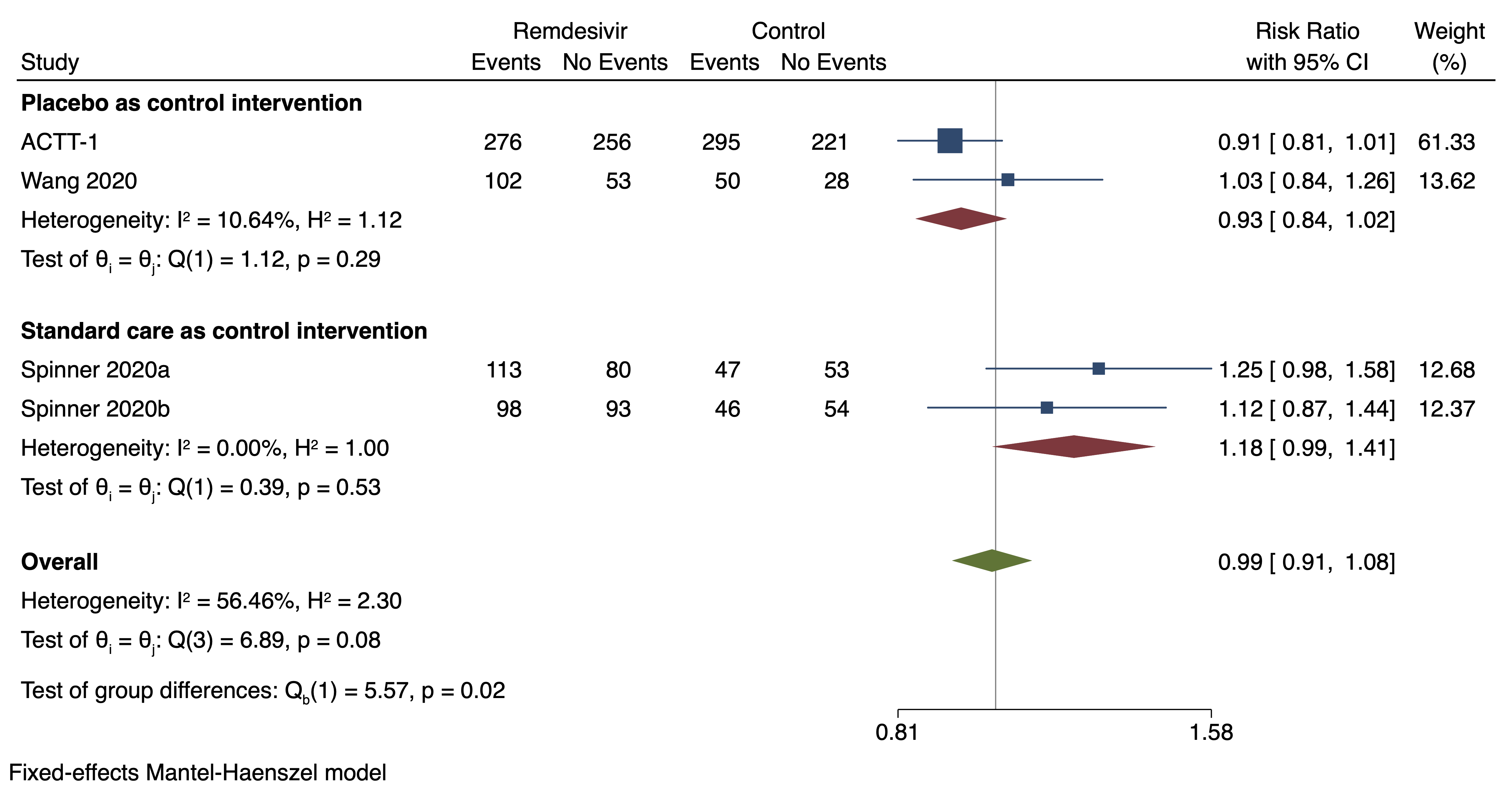

Supplement: S15 Fig — (TIFF) [file pone.0248132.s083.tiff]

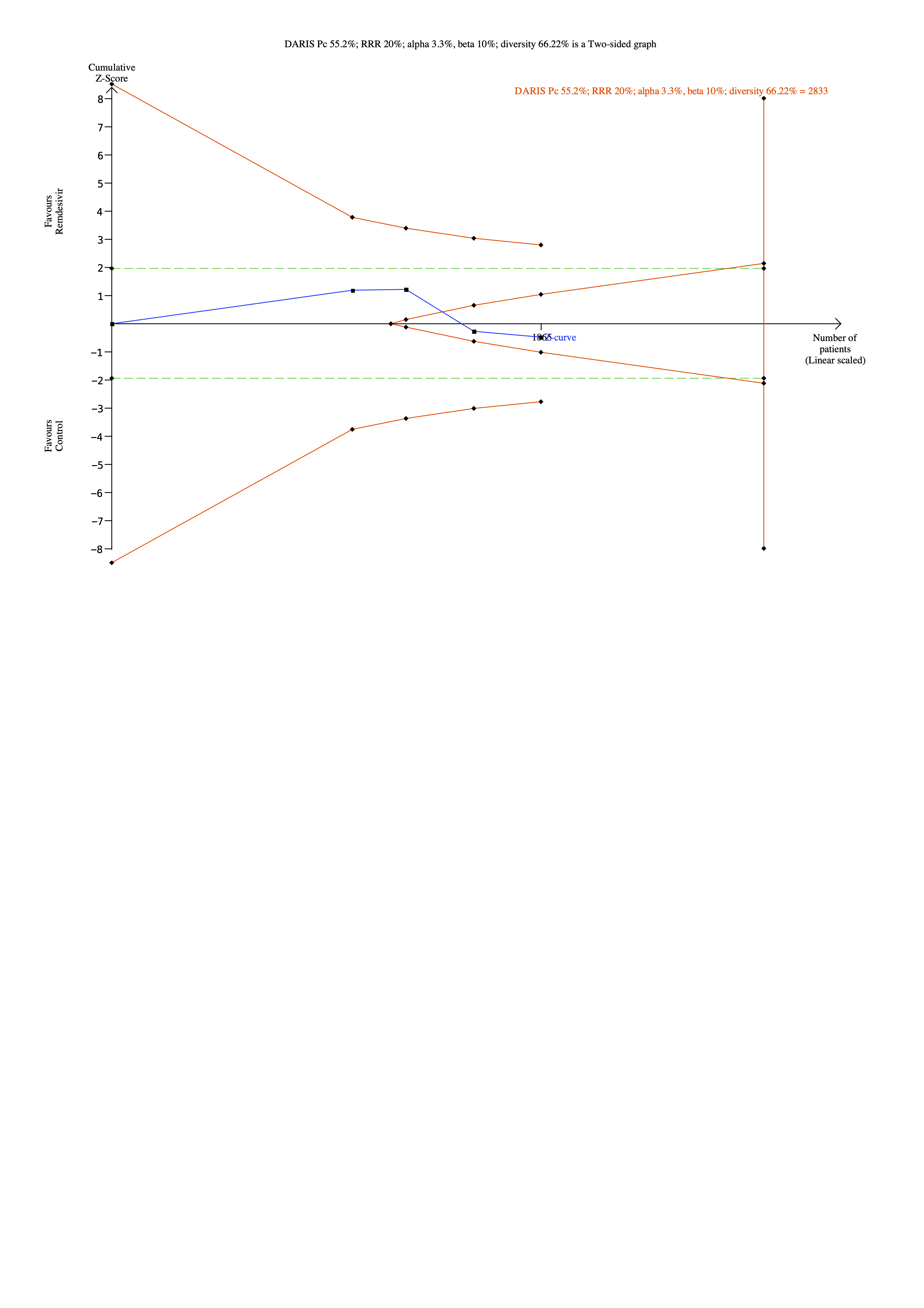

Supplement: S16 Fig — (TIFF) [file pone.0248132.s084.tiff]

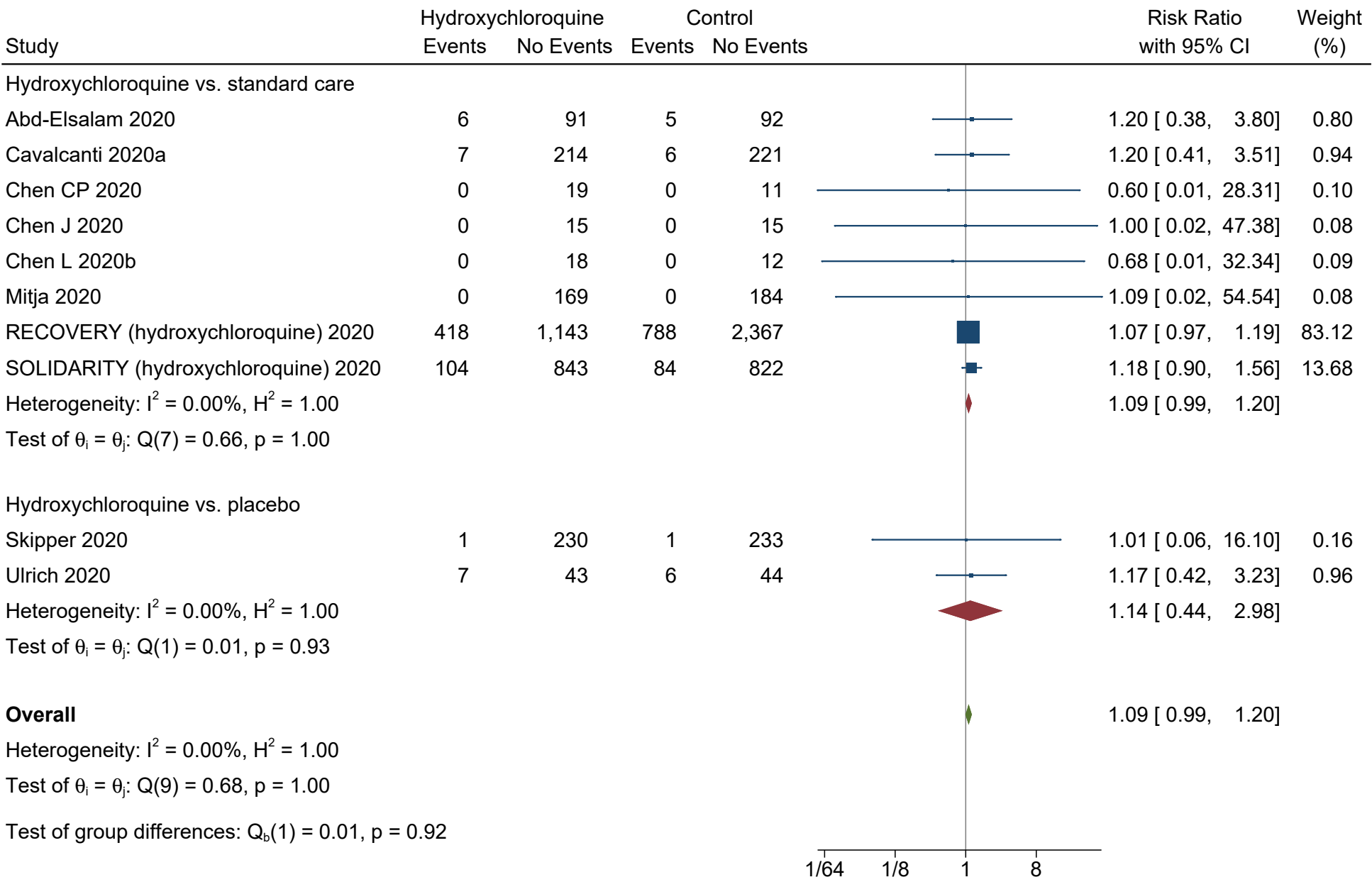

Supplement: S17 Fig — (PDF) [file pone.0248132.s085.pdf]

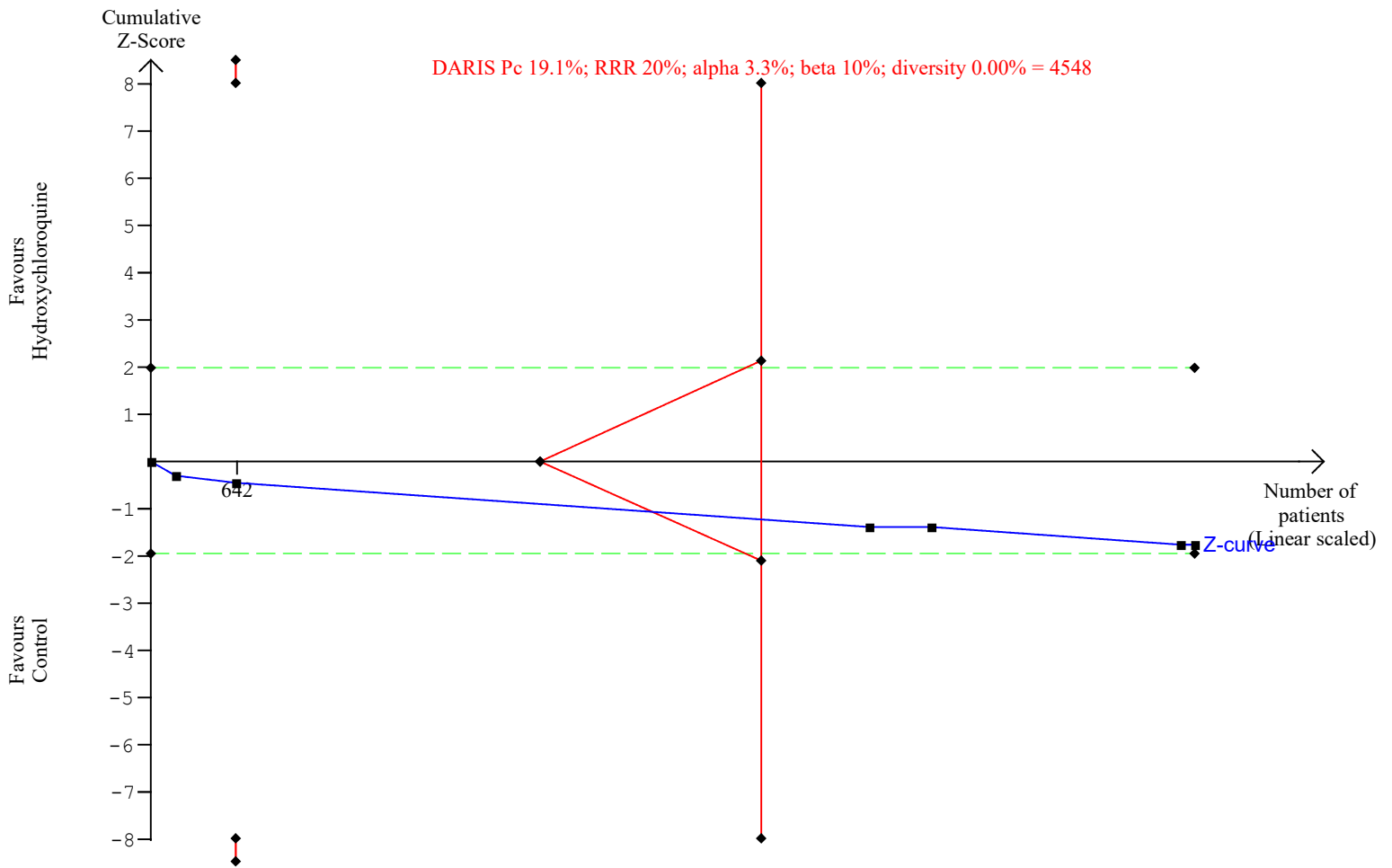

Supplement: S18 Fig — (PDF) [file pone.0248132.s086.pdf]

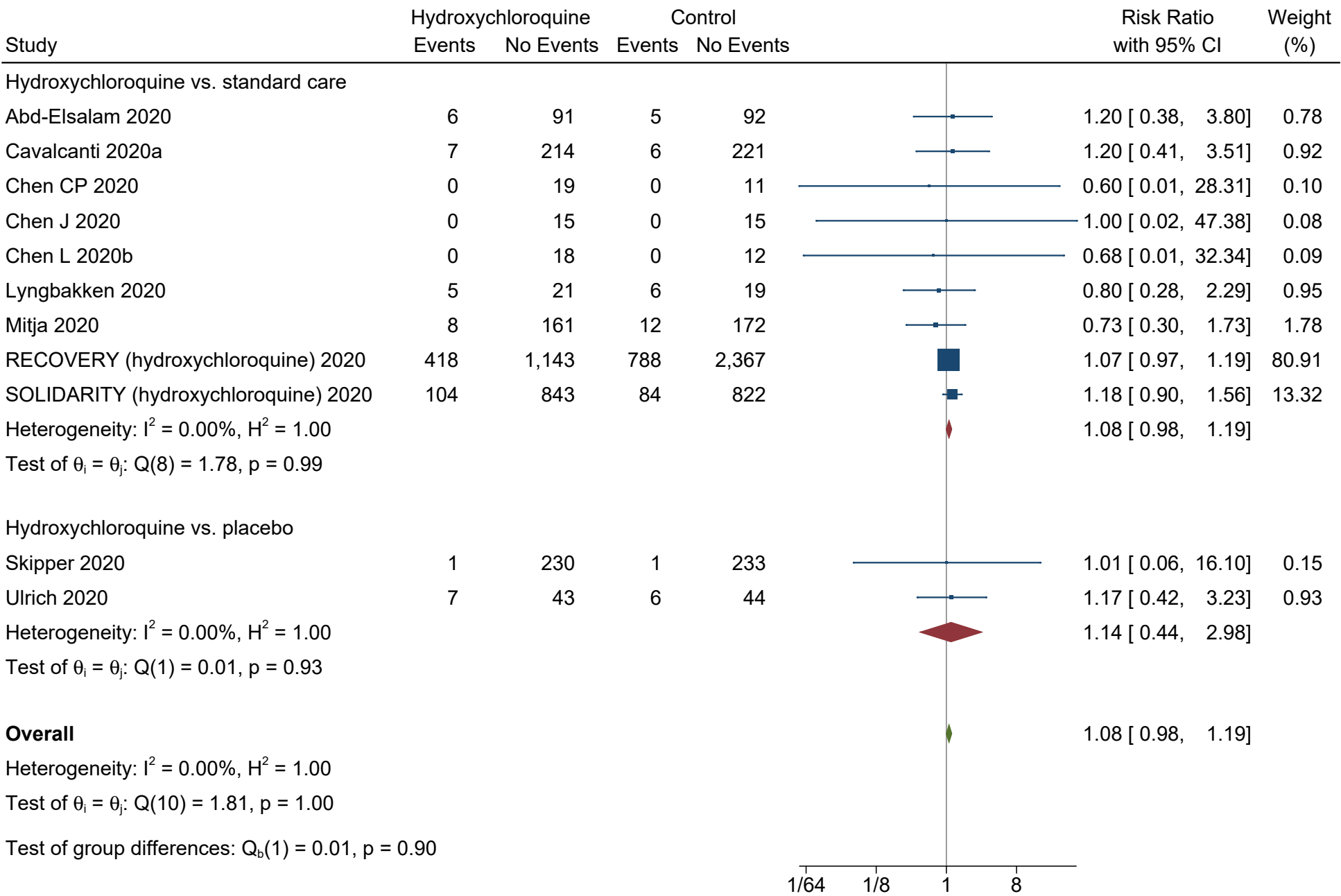

Supplement: S19 Fig — (PDF) [file pone.0248132.s087.pdf]

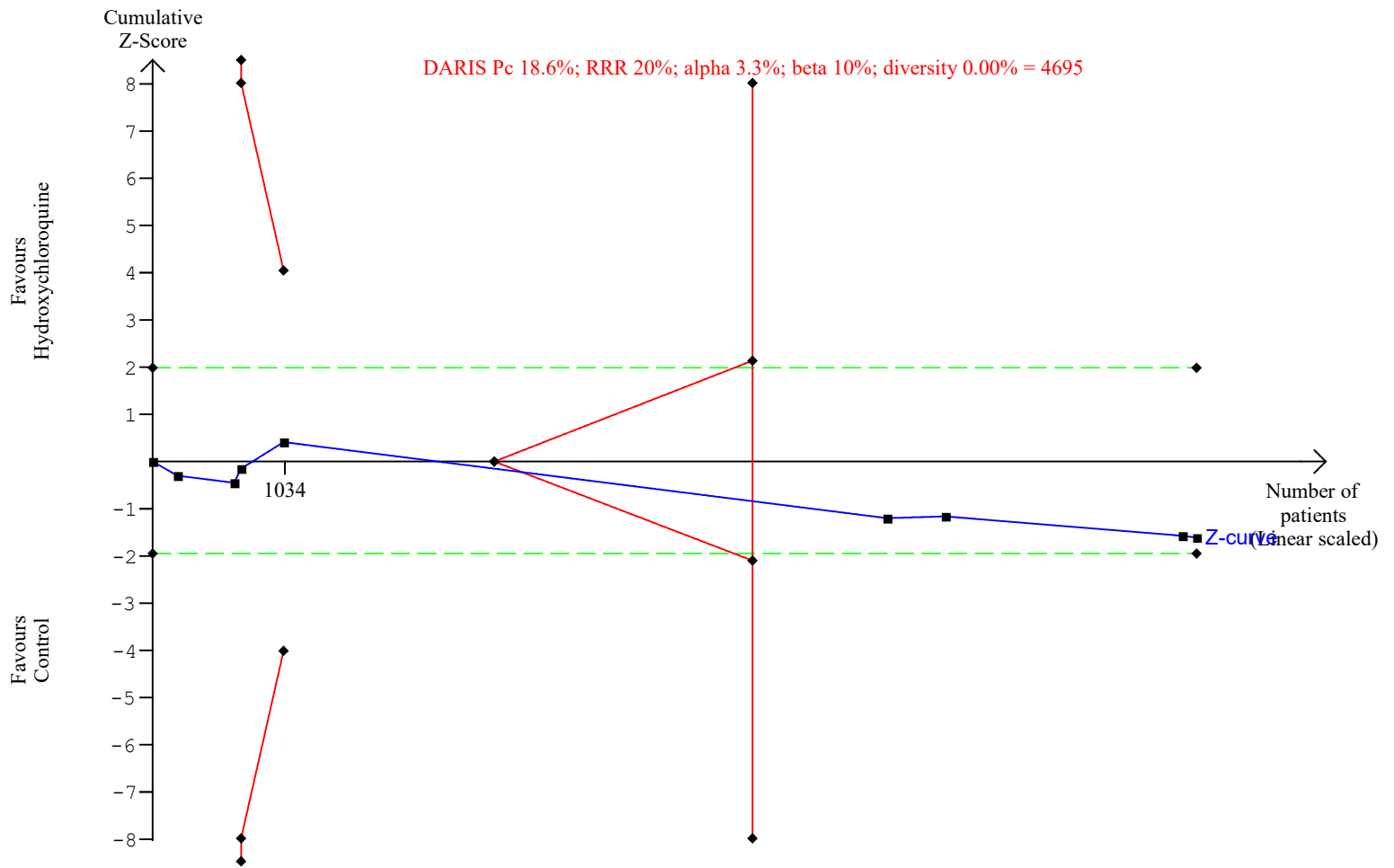

Supplement: S20 Fig — (PDF) [file pone.0248132.s088.pdf]

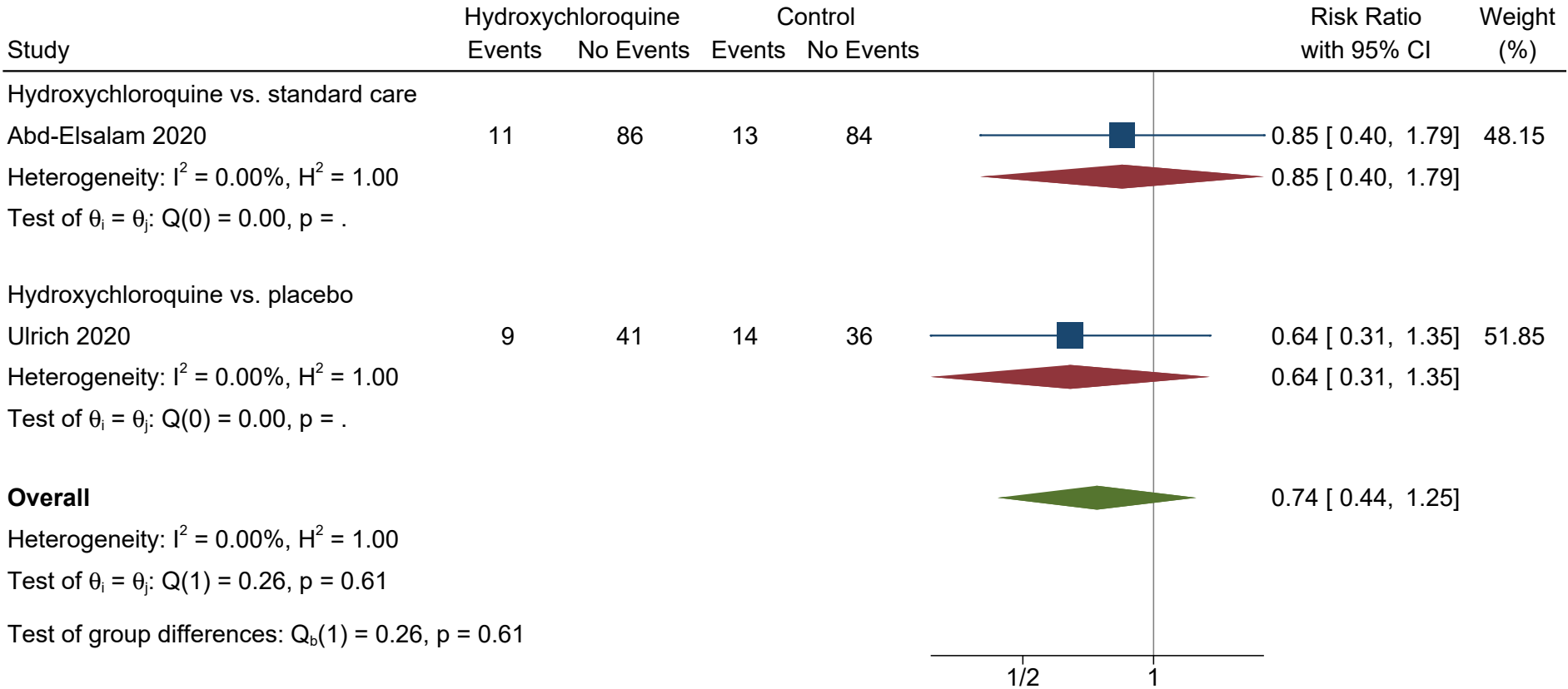

Fixed-effects Mantel-Haenszel model

Supplement: S21 Fig — (PDF) [file pone.0248132.s089.pdf]

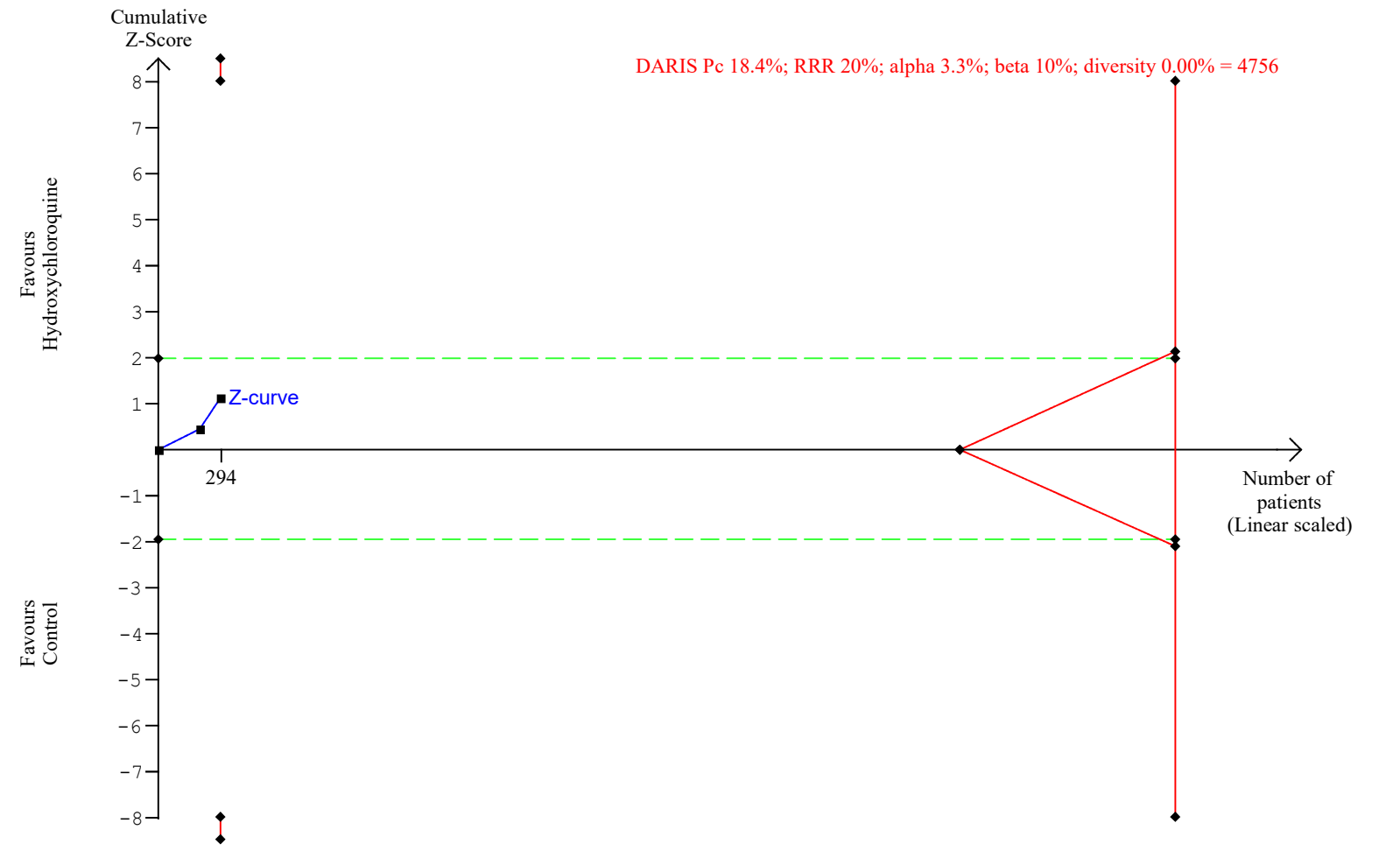

Supplement: S22 Fig — (PDF) [file pone.0248132.s090.pdf]

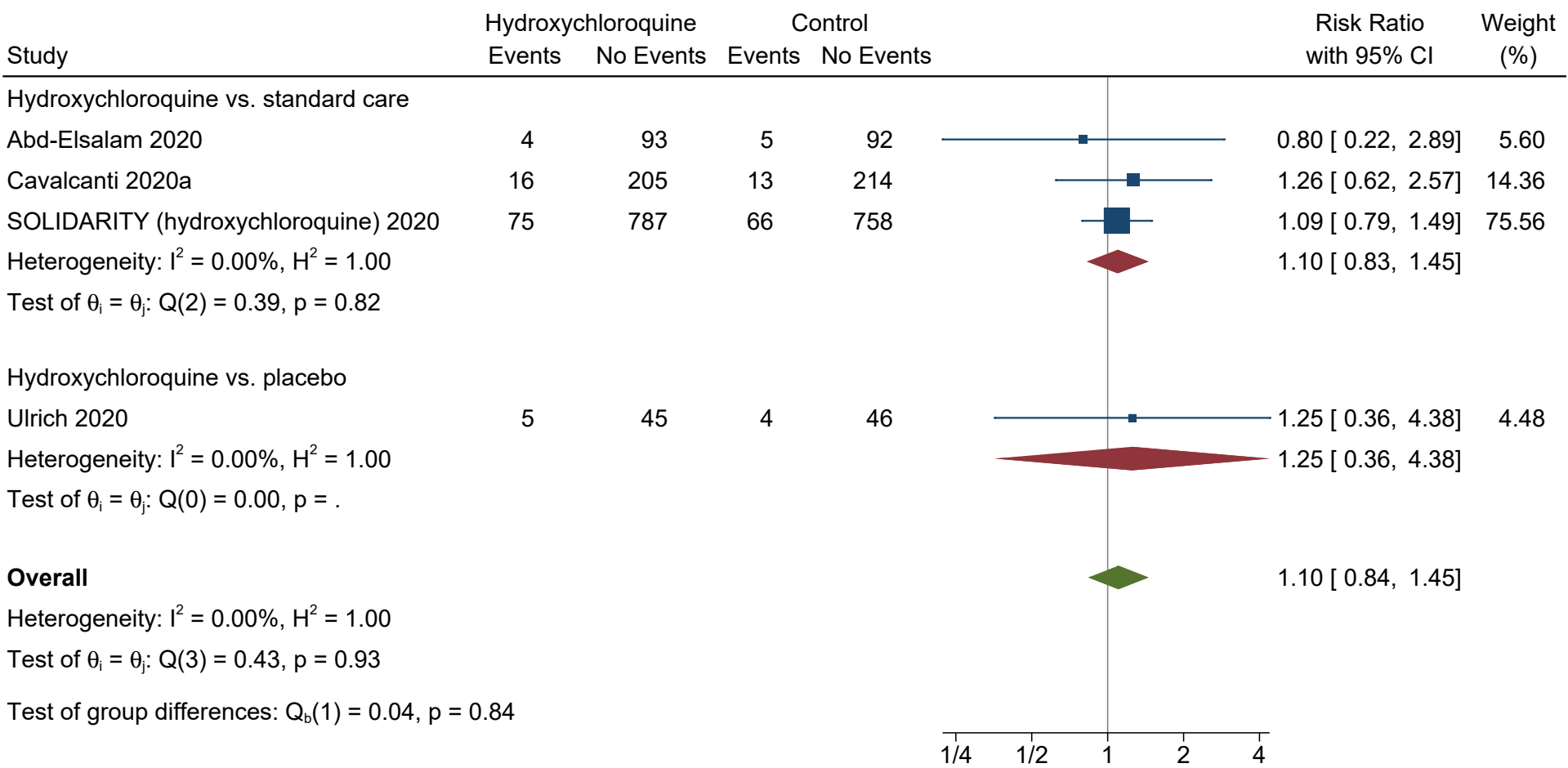

Fixed-effects Mantel-Haenszel model

Supplement: S23 Fig — (PDF) [file pone.0248132.s091.pdf]

DARIS Pc 7.3%; RRR 20%; alpha 3.3%; beta 10%; diversity 0.00% is a Two-sided graph

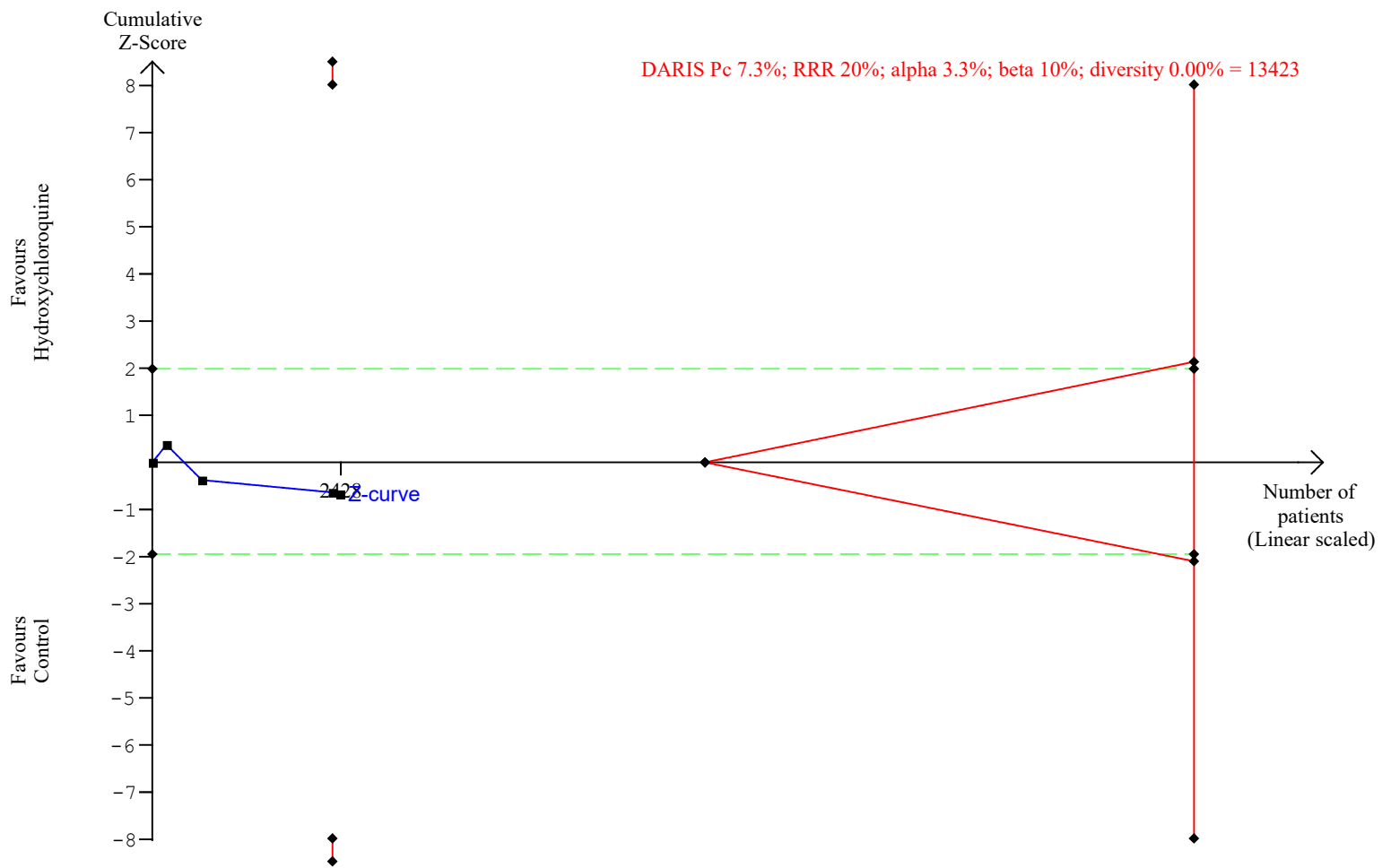

Supplement: S24 Fig — (PDF) [file pone.0248132.s092.pdf]

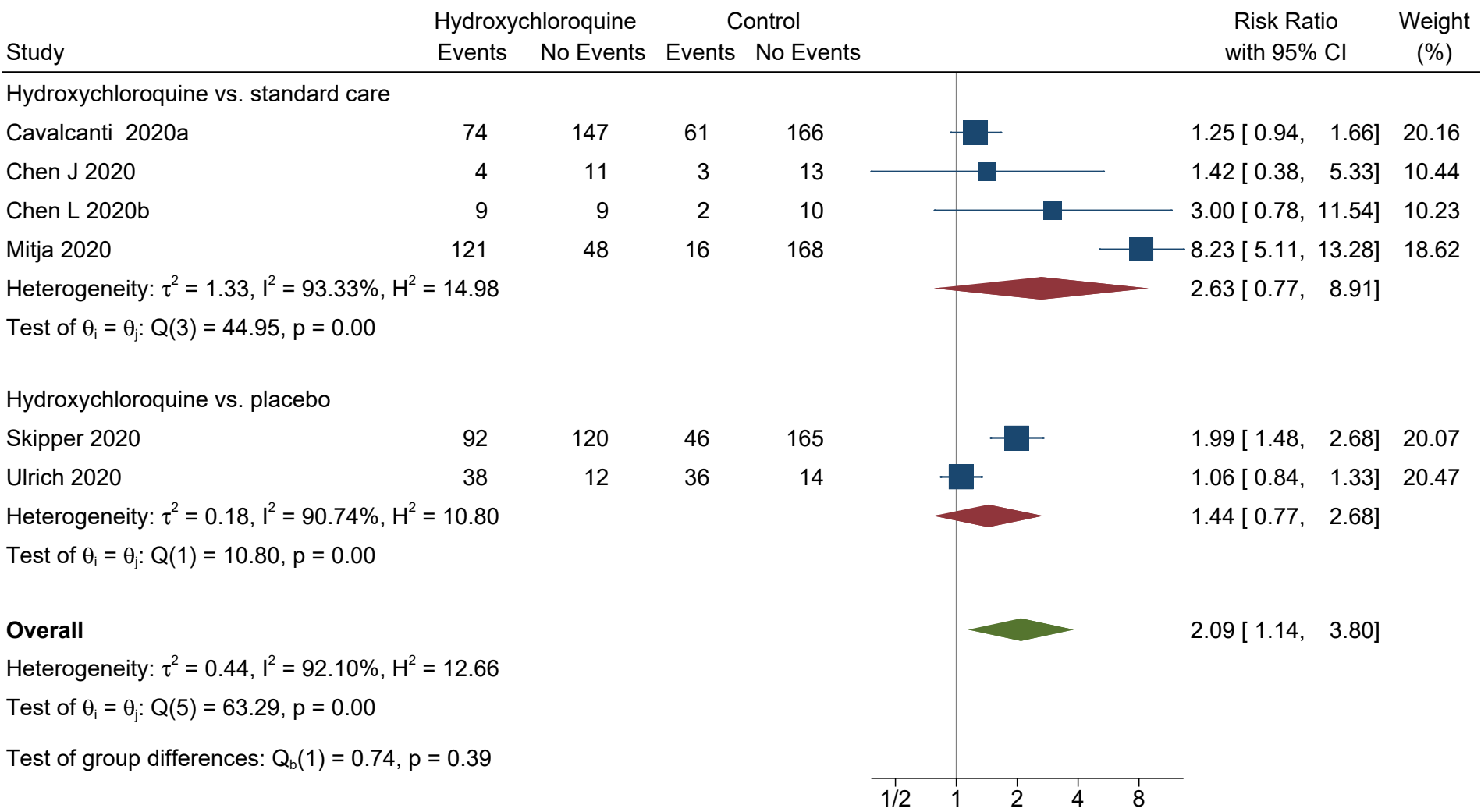

Supplement: S25 Fig — (PDF) [file pone.0248132.s093.pdf]

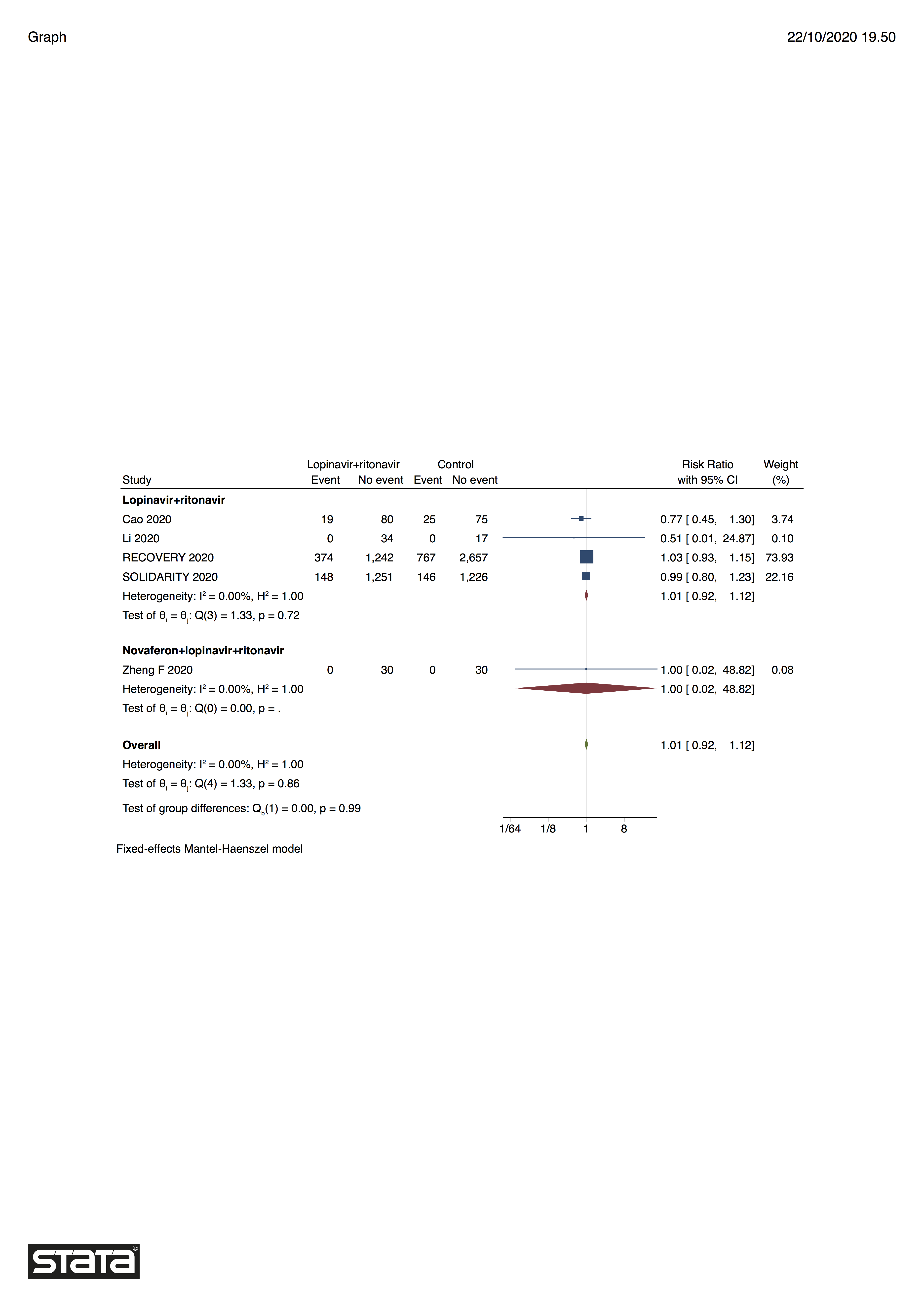

Supplement: S26 Fig — (TIFF) [file pone.0248132.s094.tiff]

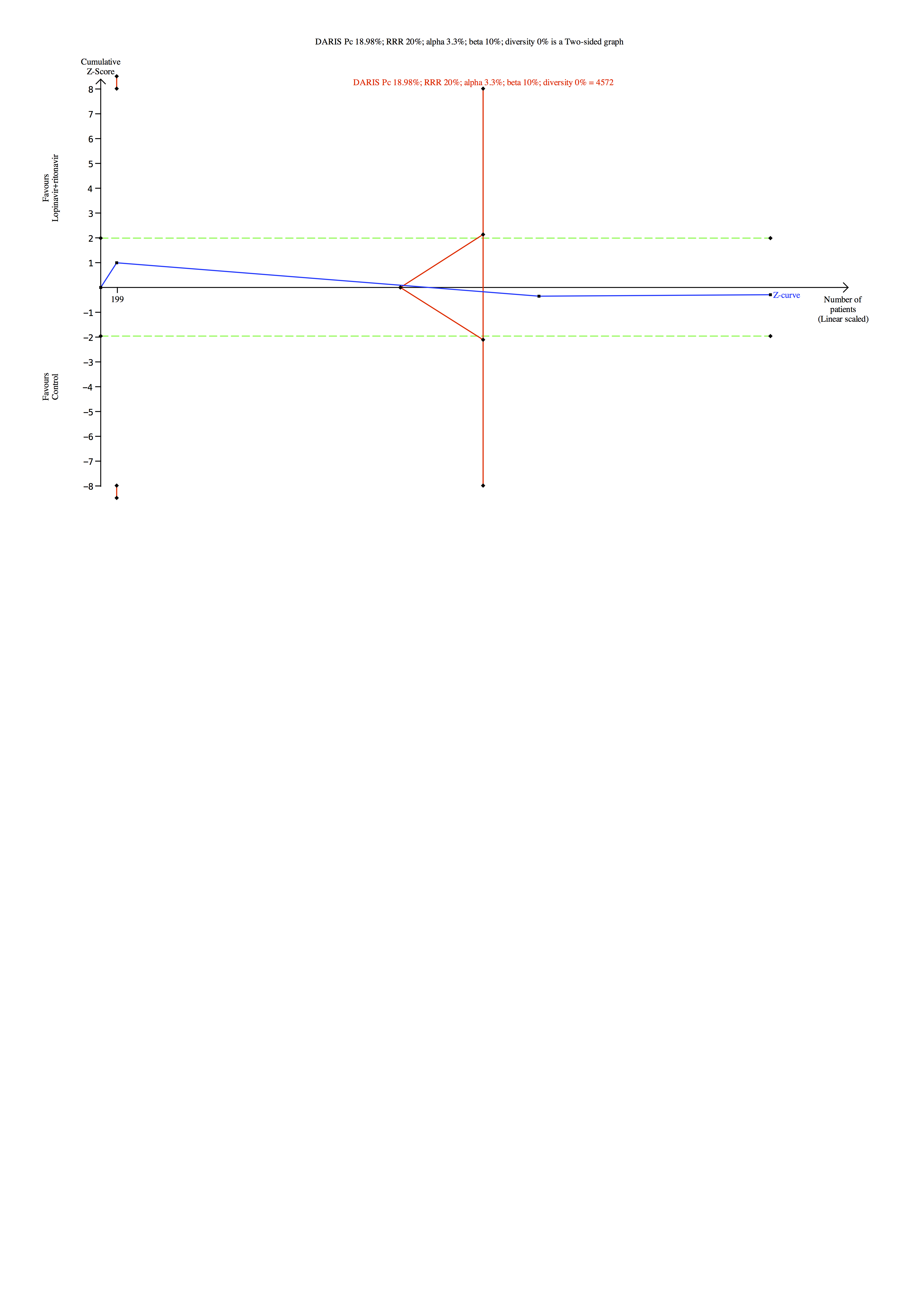

Supplement: S27 Fig — (TIFF) [file pone.0248132.s095.tiff]

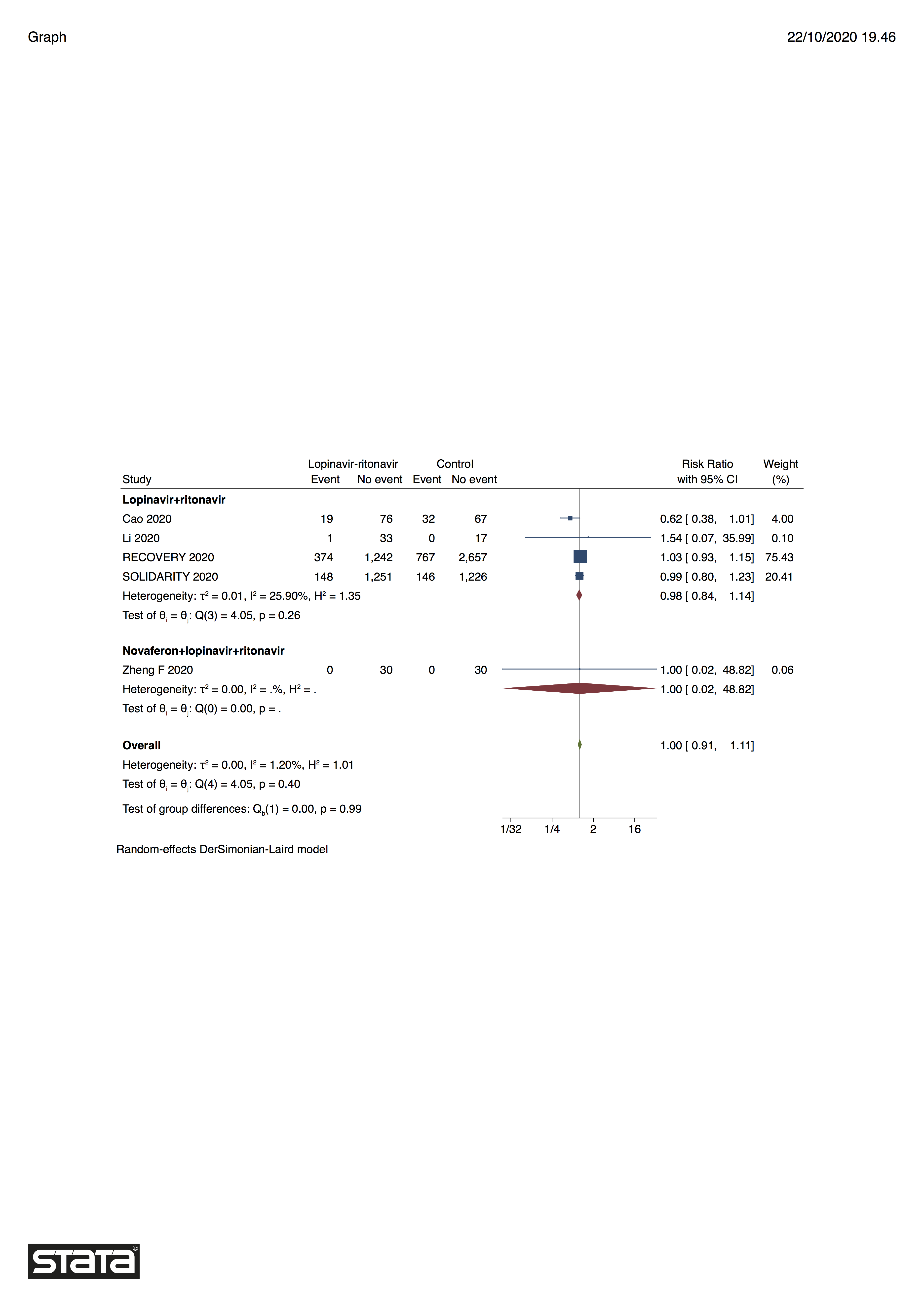

Supplement: S28 Fig — (TIFF) [file pone.0248132.s096.tiff]

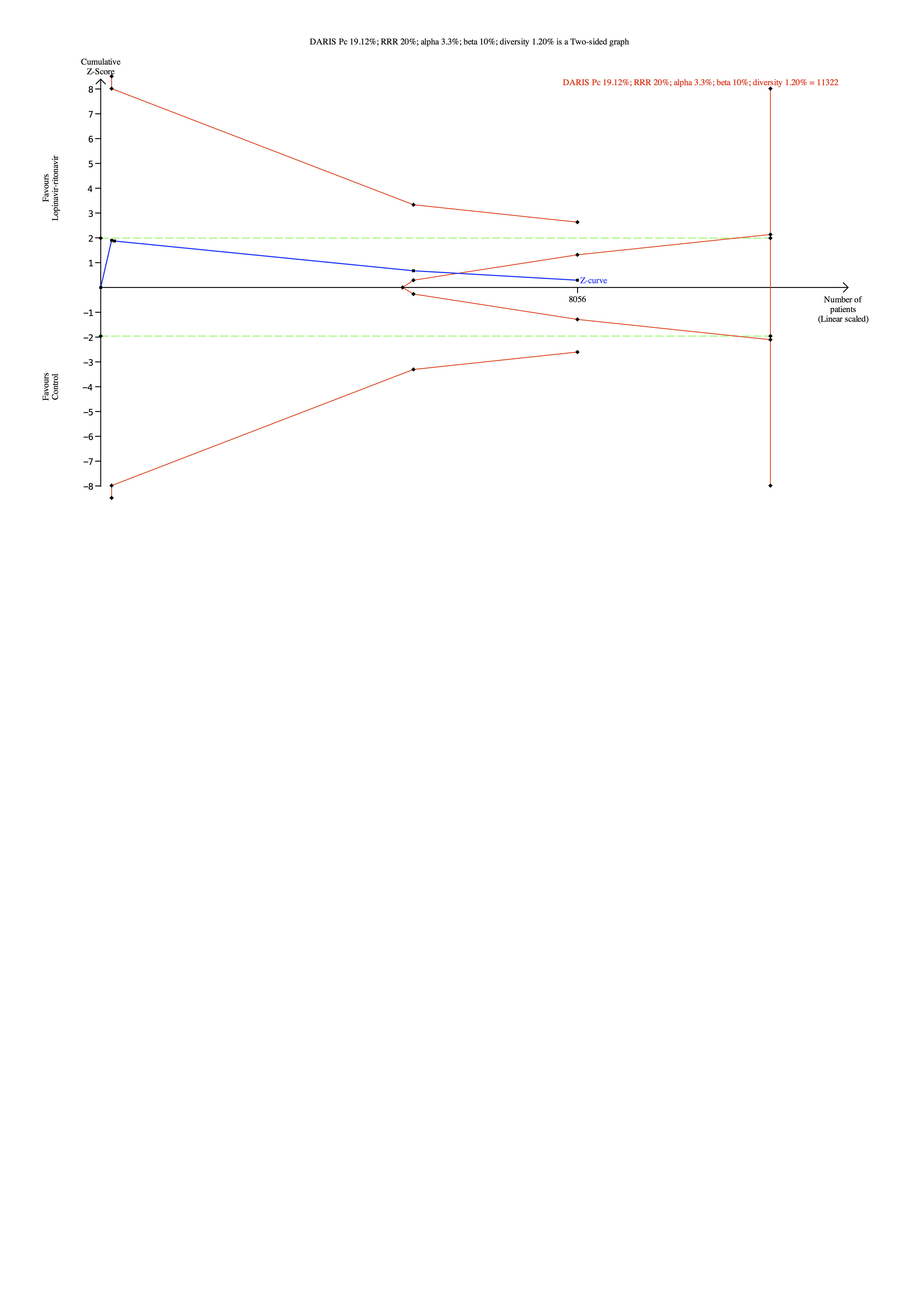

Supplement: S29 Fig — (TIFF) [file pone.0248132.s097.tiff]

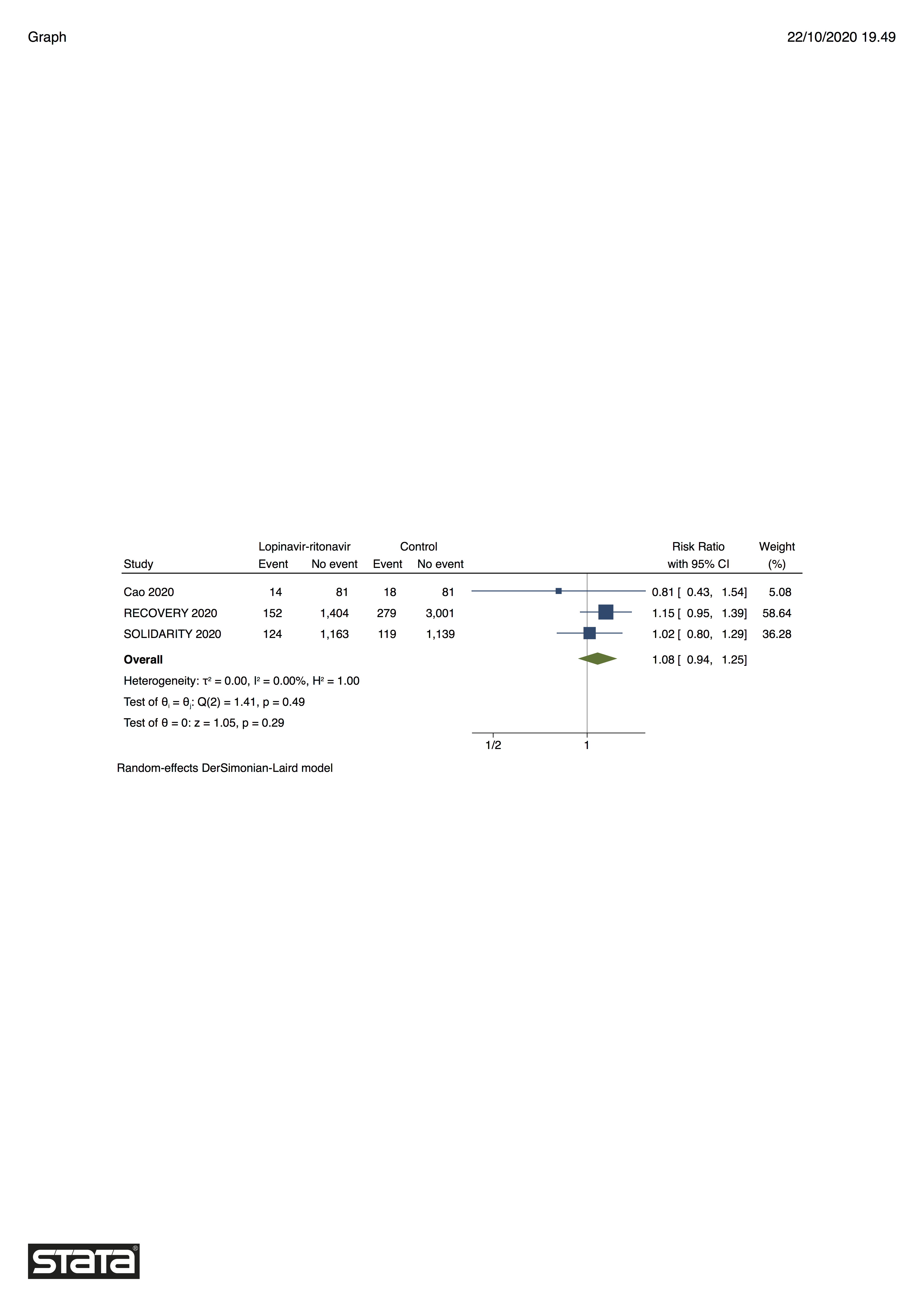

Supplement: S30 Fig — (TIFF) [file pone.0248132.s098.tiff]

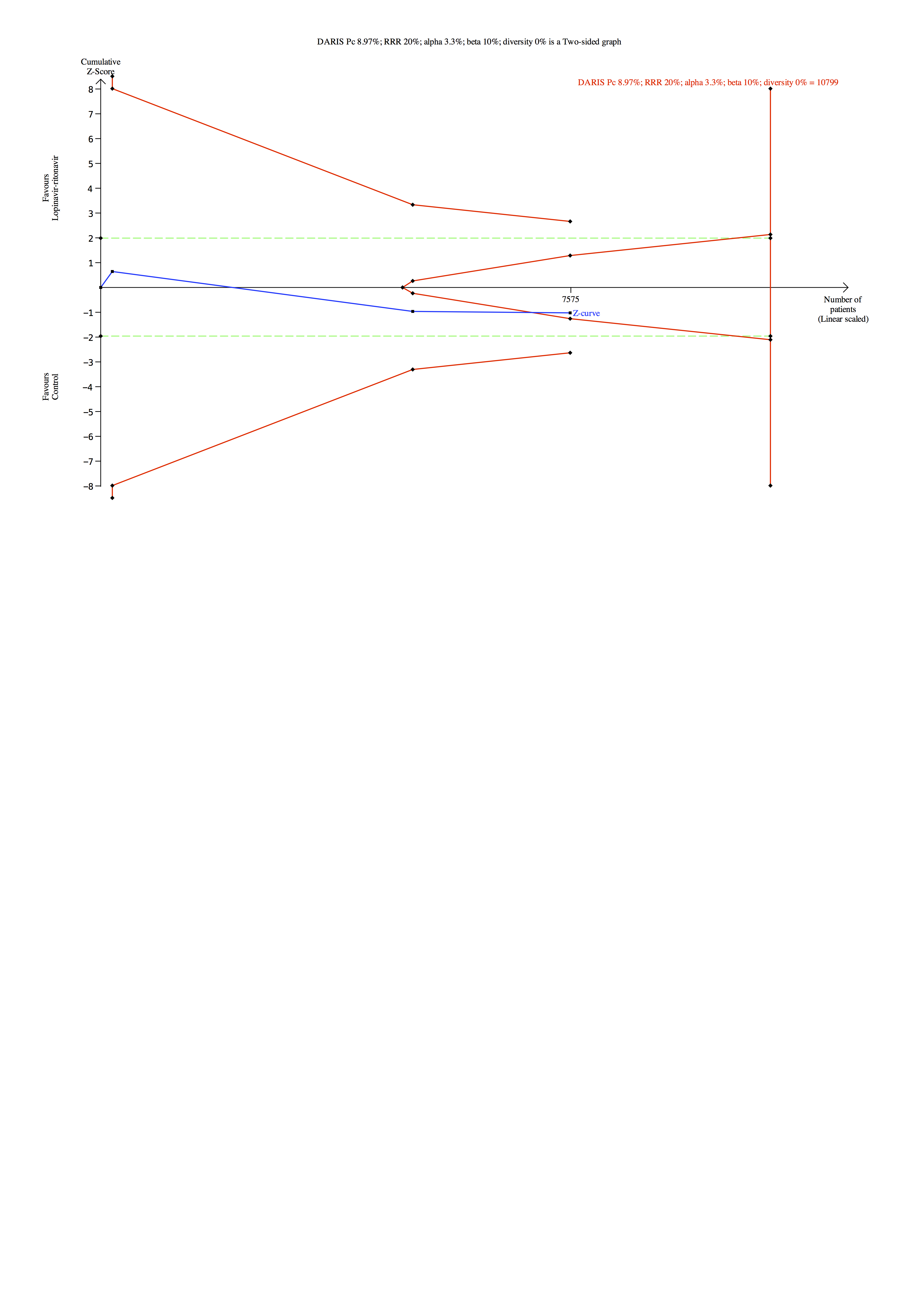

Supplement: S31 Fig — (TIFF) [file pone.0248132.s099.tiff]

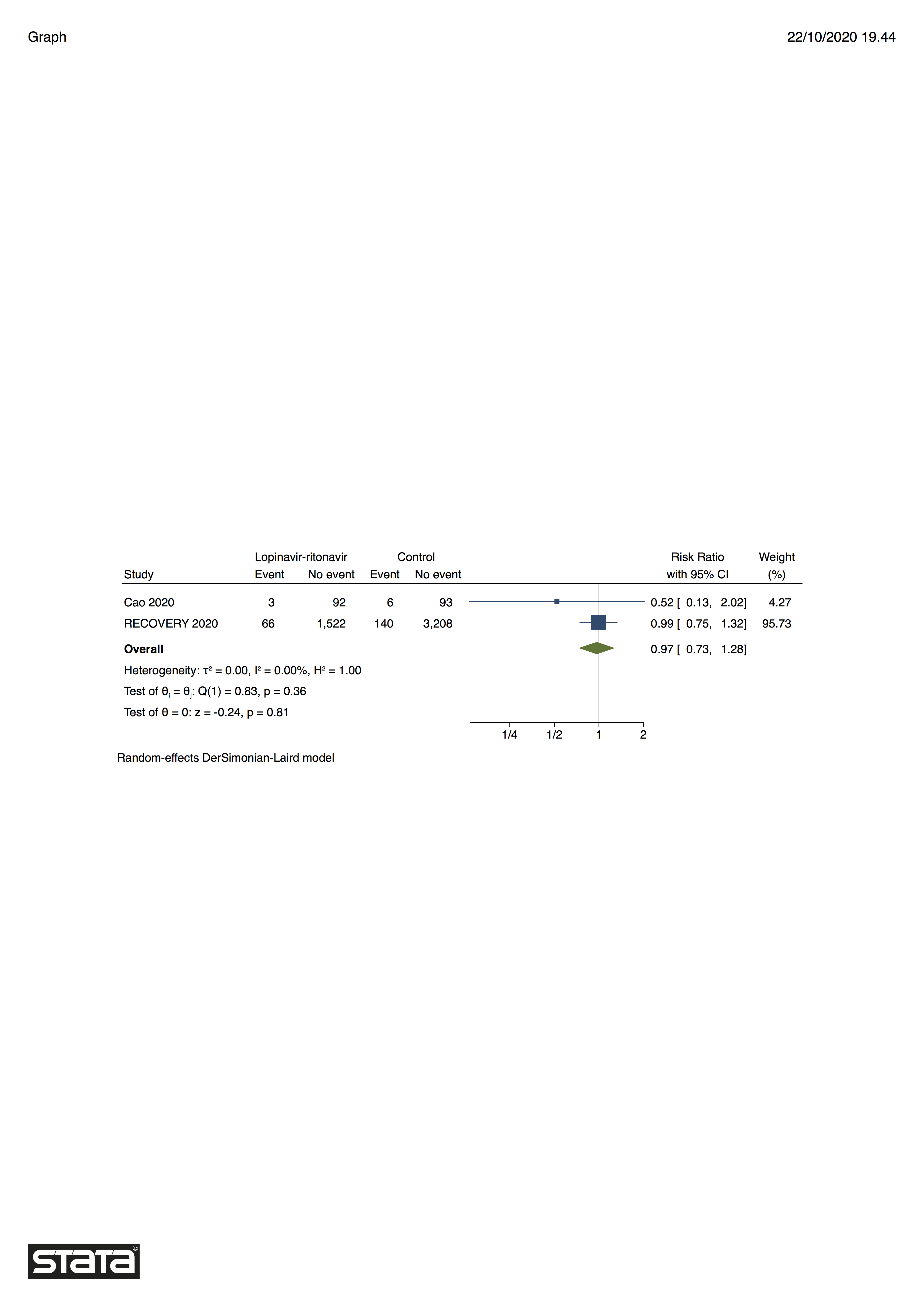

Supplement: S32 Fig — (TIFF) [file pone.0248132.s100.tiff]

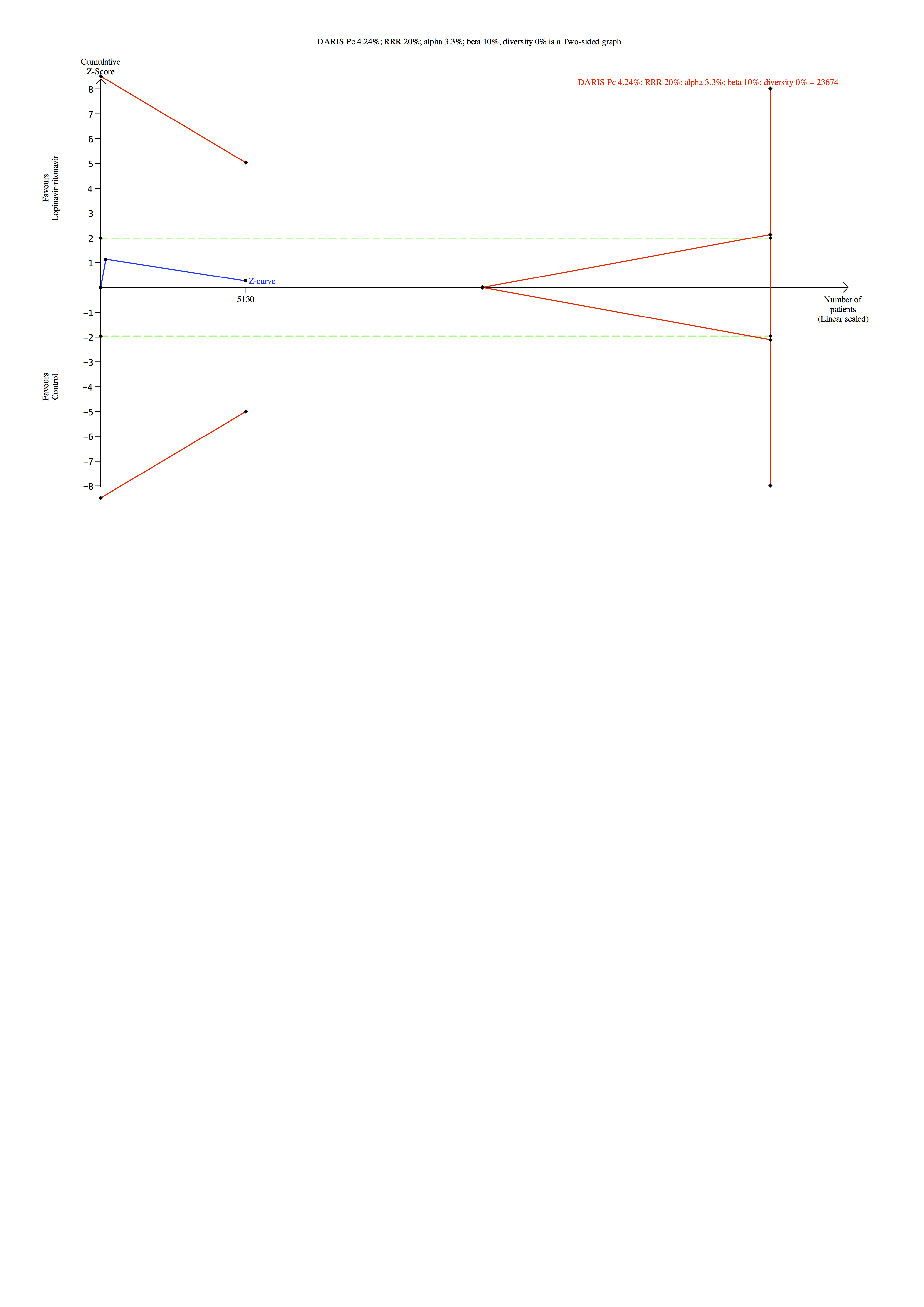

Supplement: S33 Fig — (TIFF) [file pone.0248132.s101.tiff]

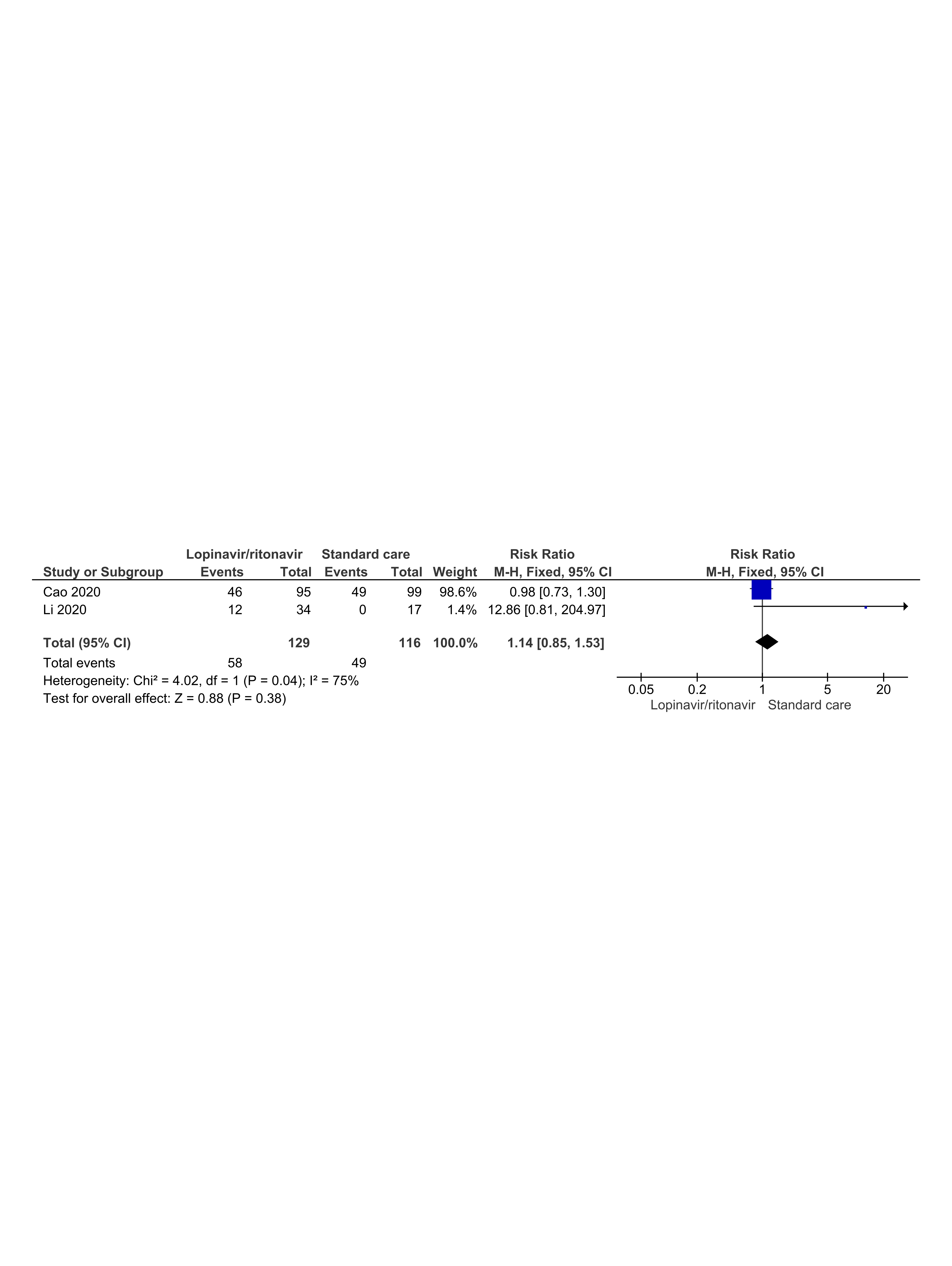

Supplement: S34 Fig — (TIFF) [file pone.0248132.s102.tiff]

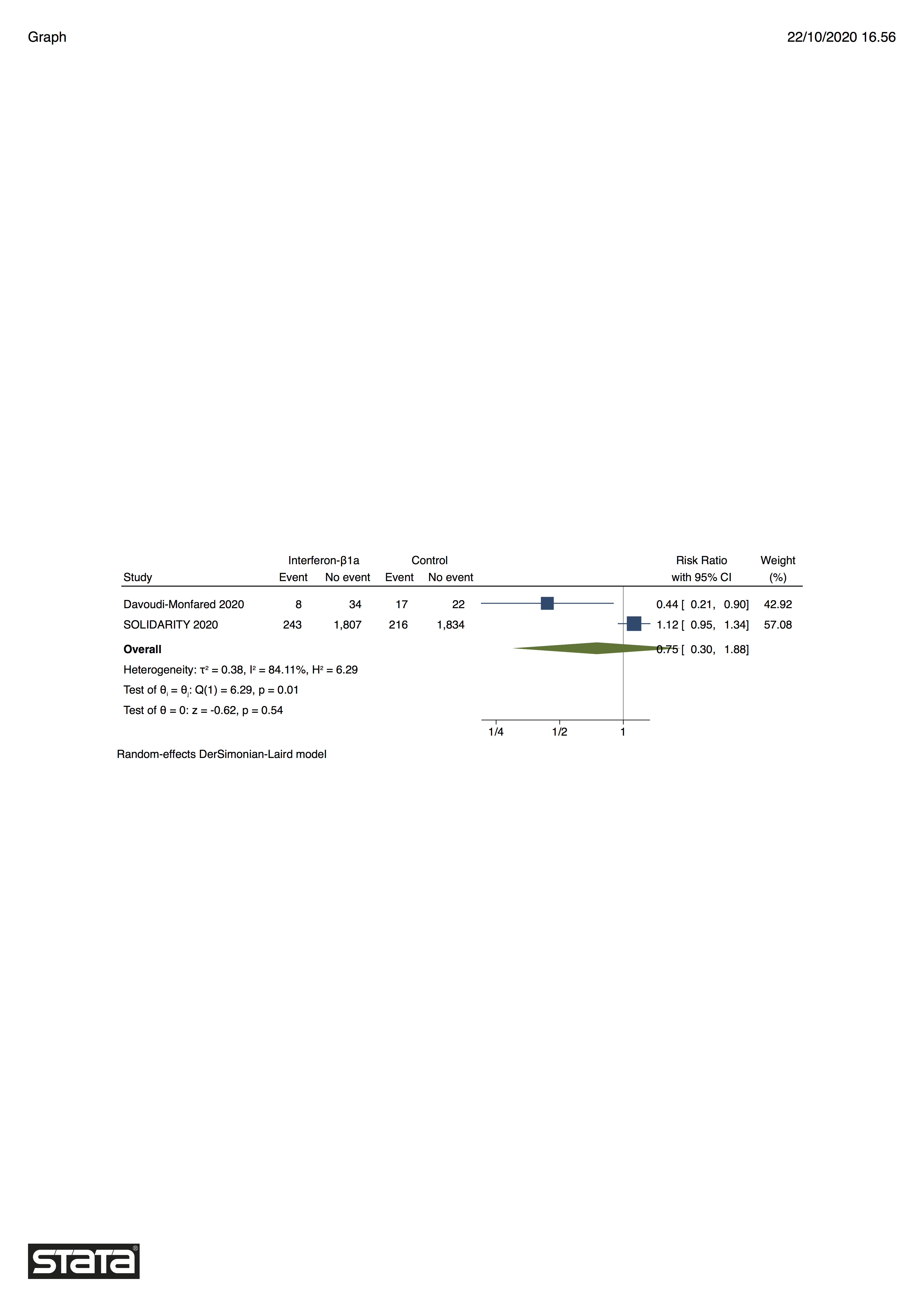

Supplement: S35 Fig — (TIFF) [file pone.0248132.s103.tiff]

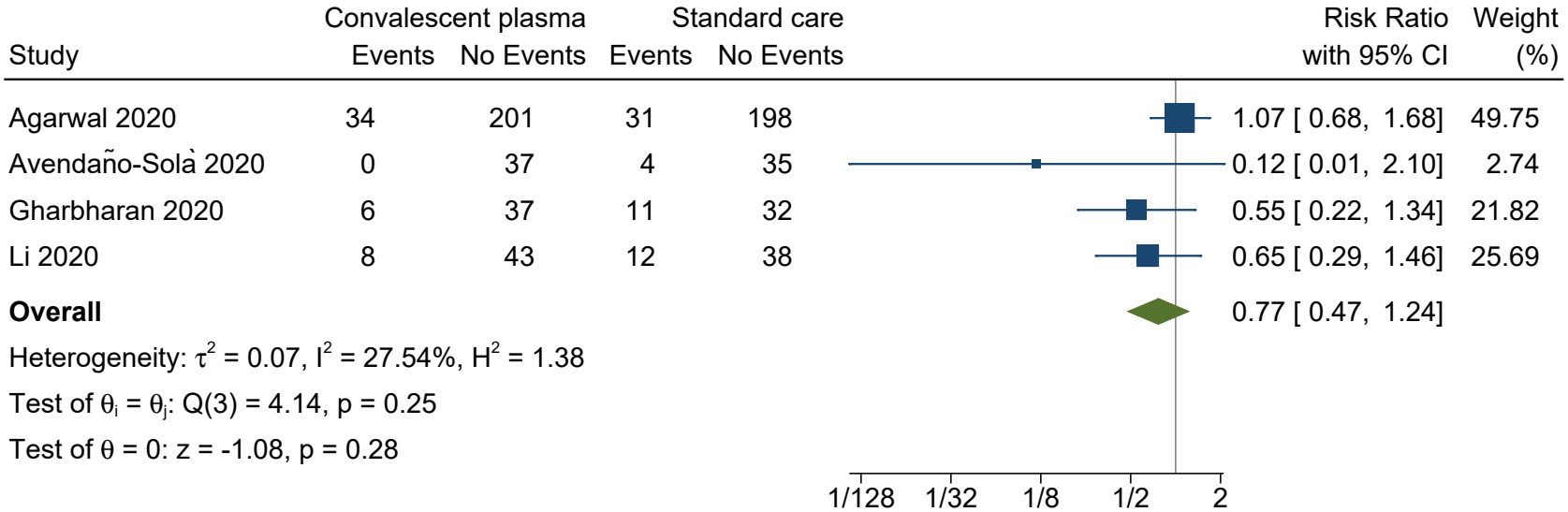

Random-effects DerSimonian-Laird model

Supplement: S37 Fig — (PDF) [file pone.0248132.s105.pdf]

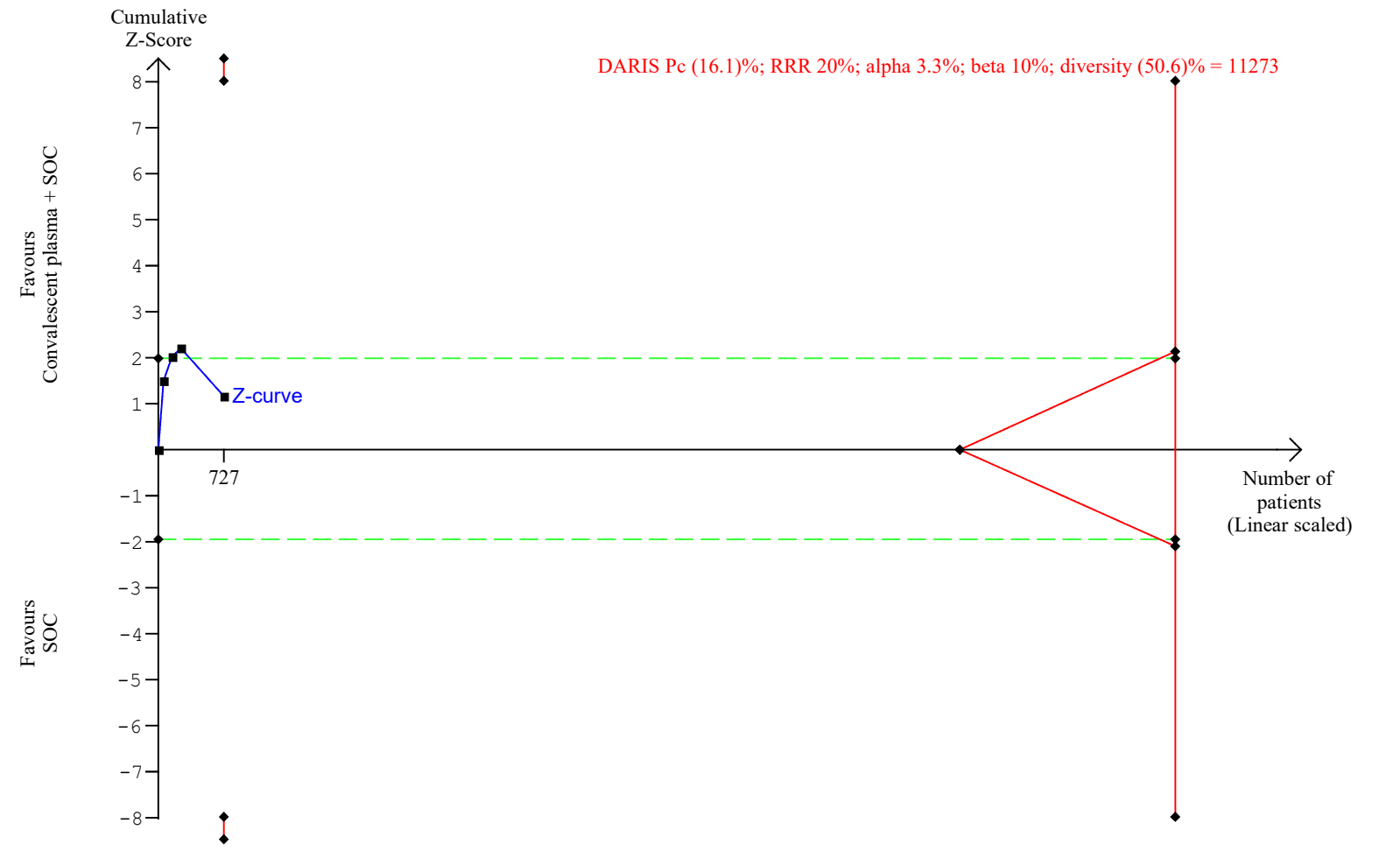

Supplement: S38 Fig — (PDF) [file pone.0248132.s106.pdf]

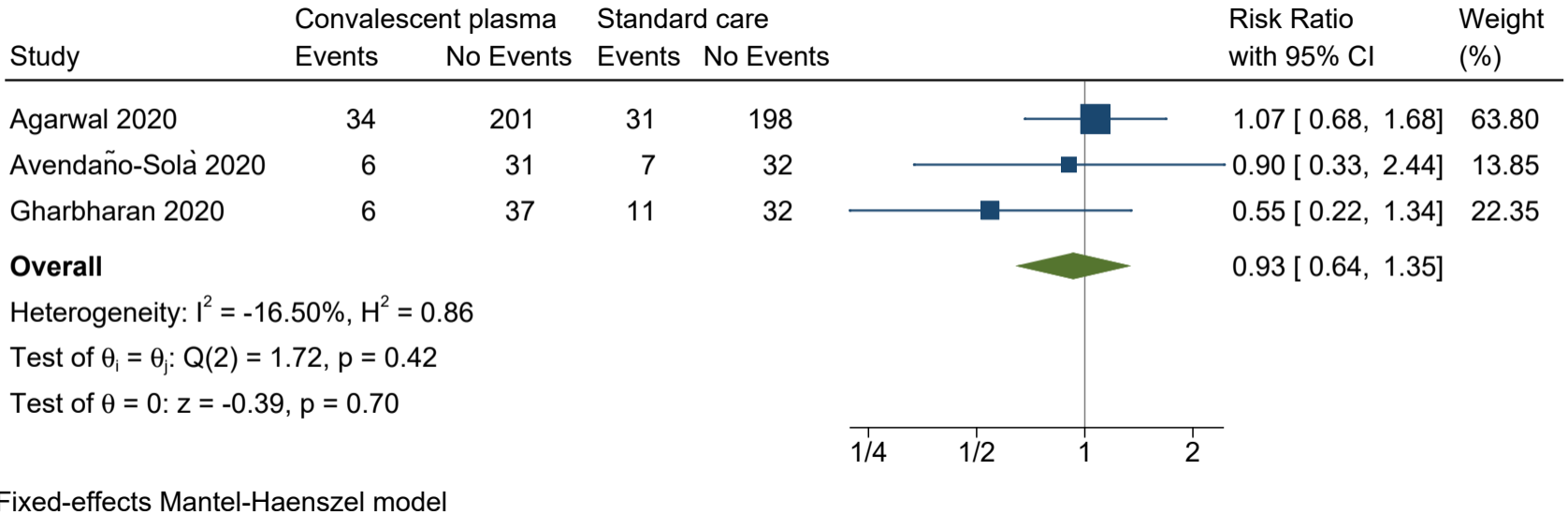

Supplement: S39 Fig — (PDF) [file pone.0248132.s107.pdf]

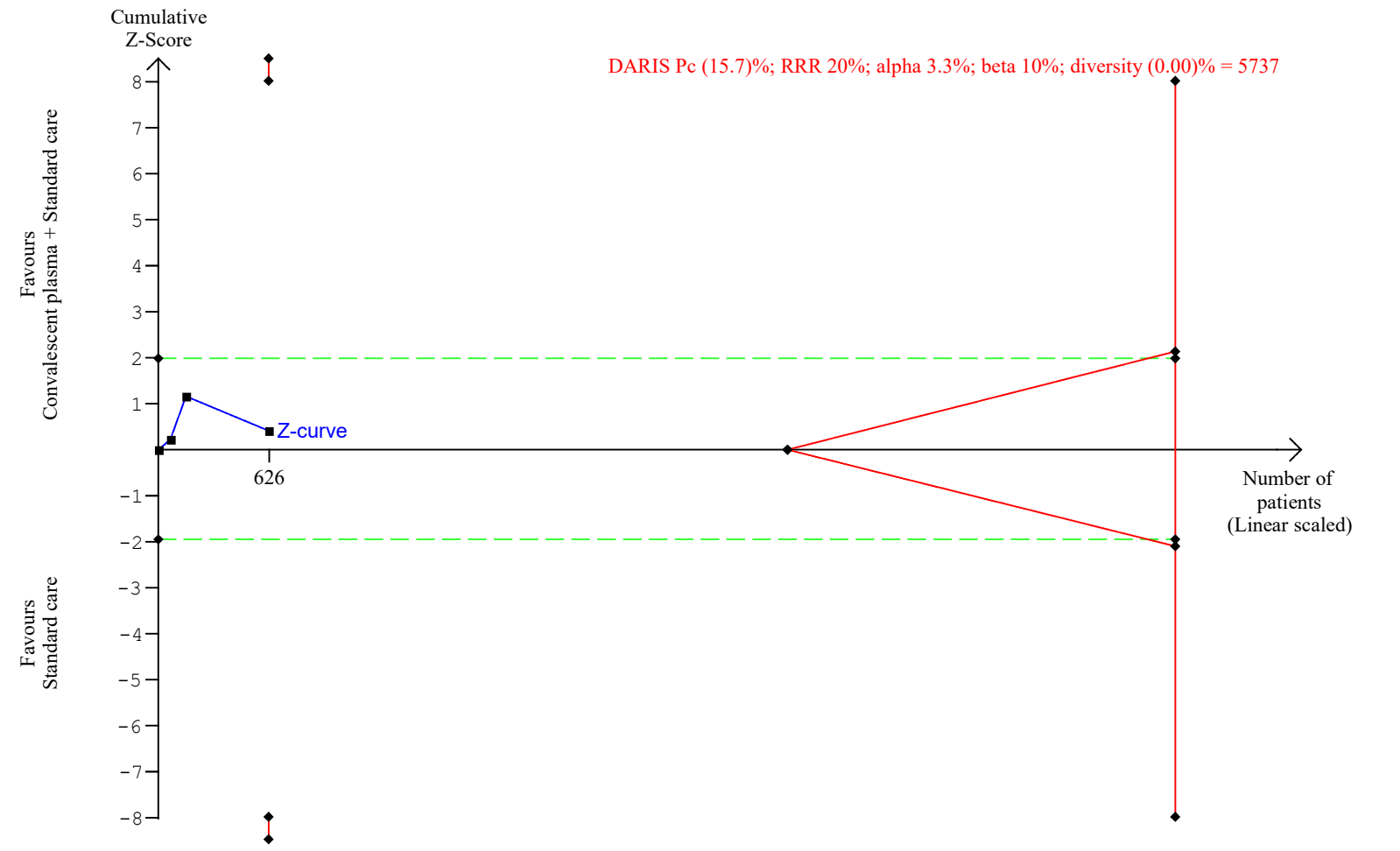

Supplement: S40 Fig — (PDF) [file pone.0248132.s108.pdf]

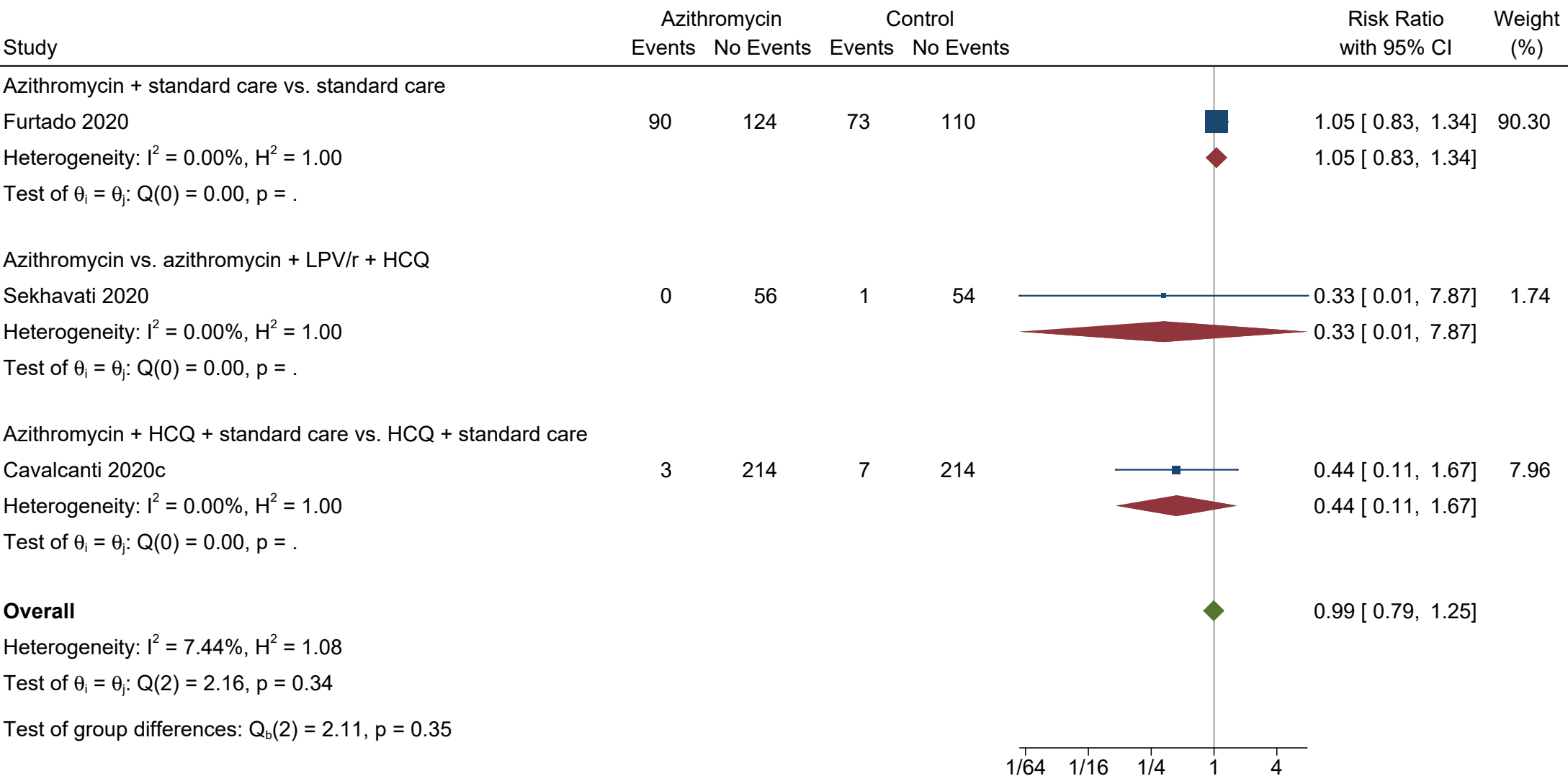

Supplement: S41 Fig — (PDF) [file pone.0248132.s109.pdf]

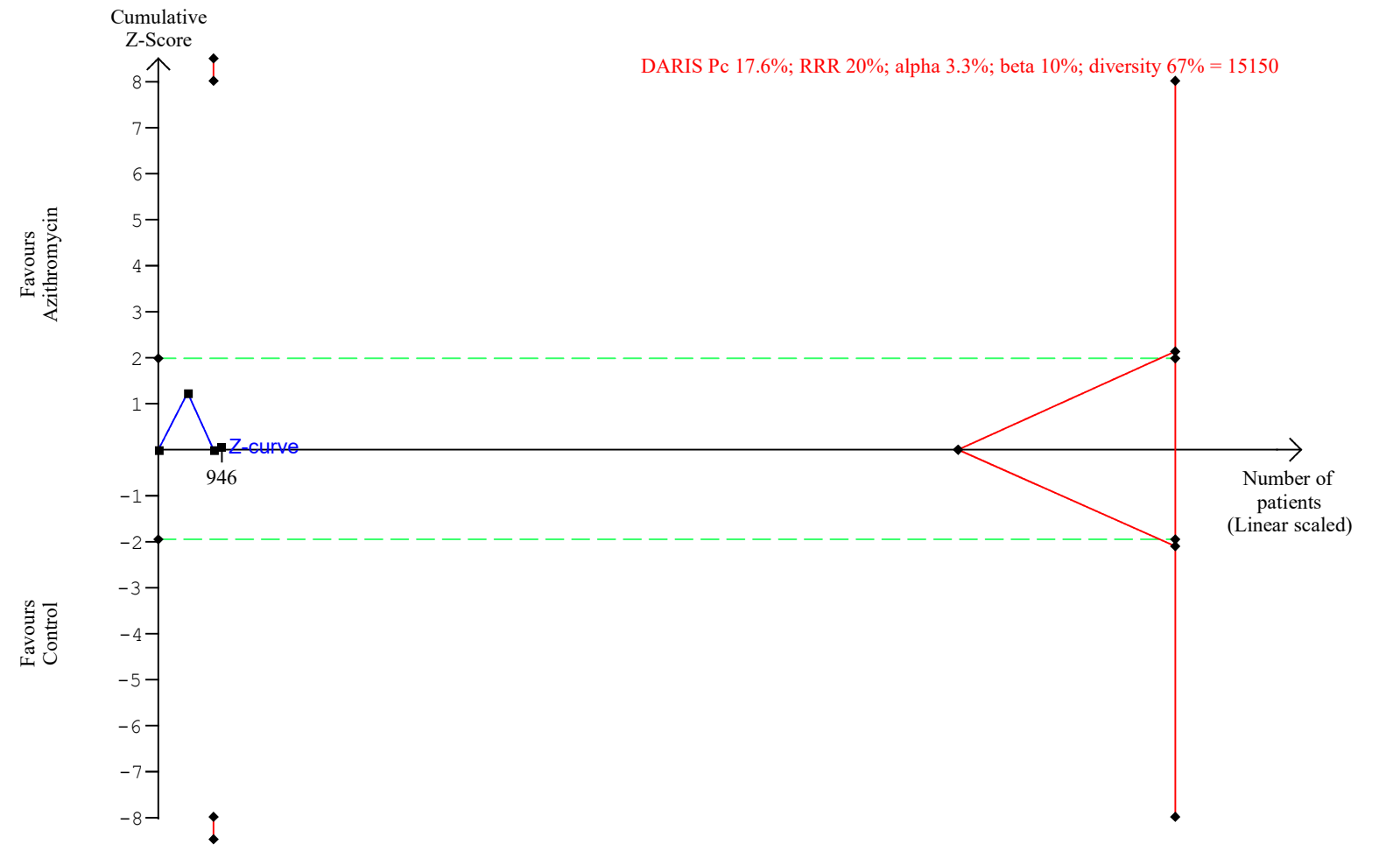

Supplement: S42 Fig — (PDF) [file pone.0248132.s110.pdf]

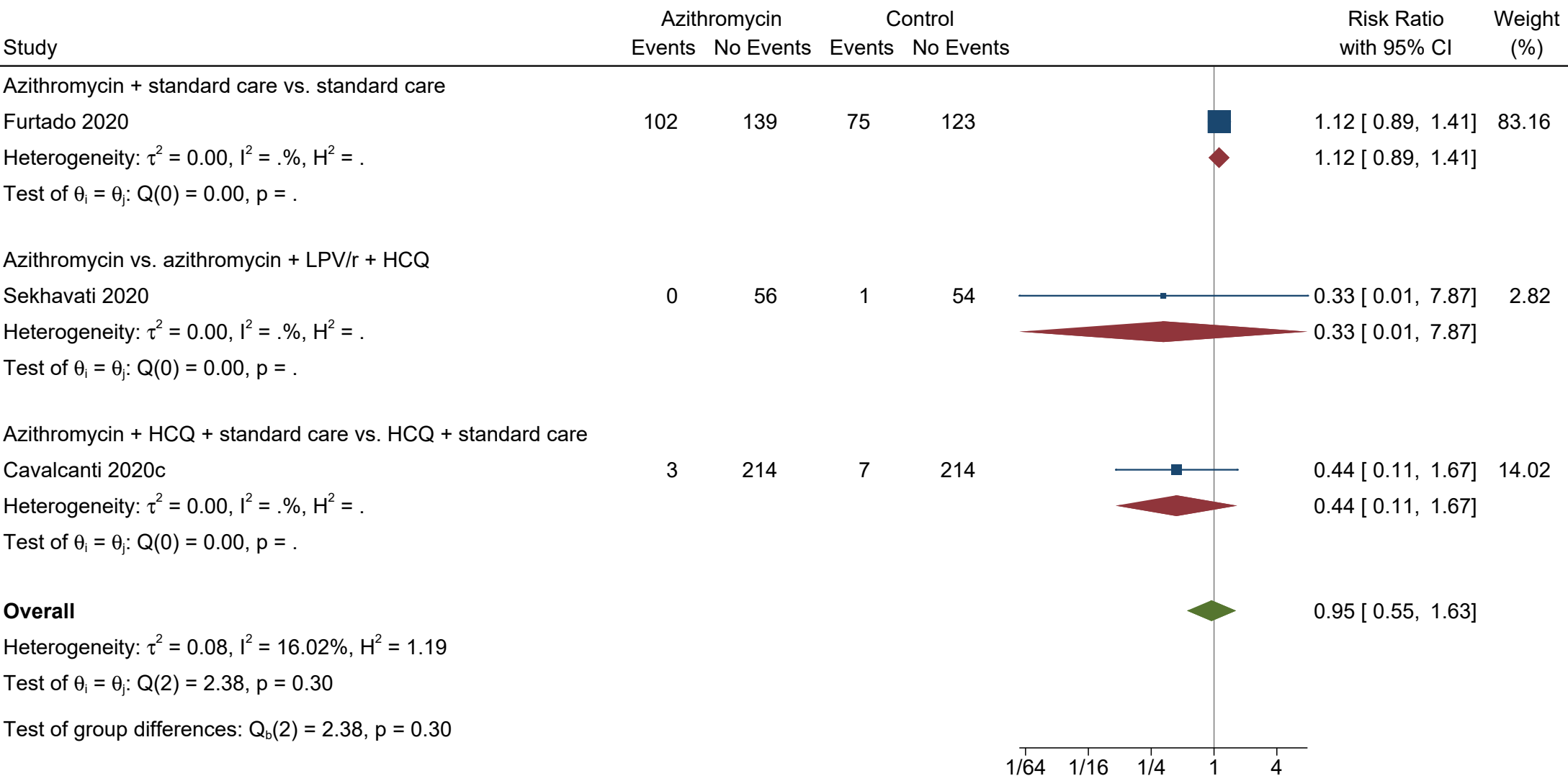

Supplement: S43 Fig — (PDF) [file pone.0248132.s111.pdf]

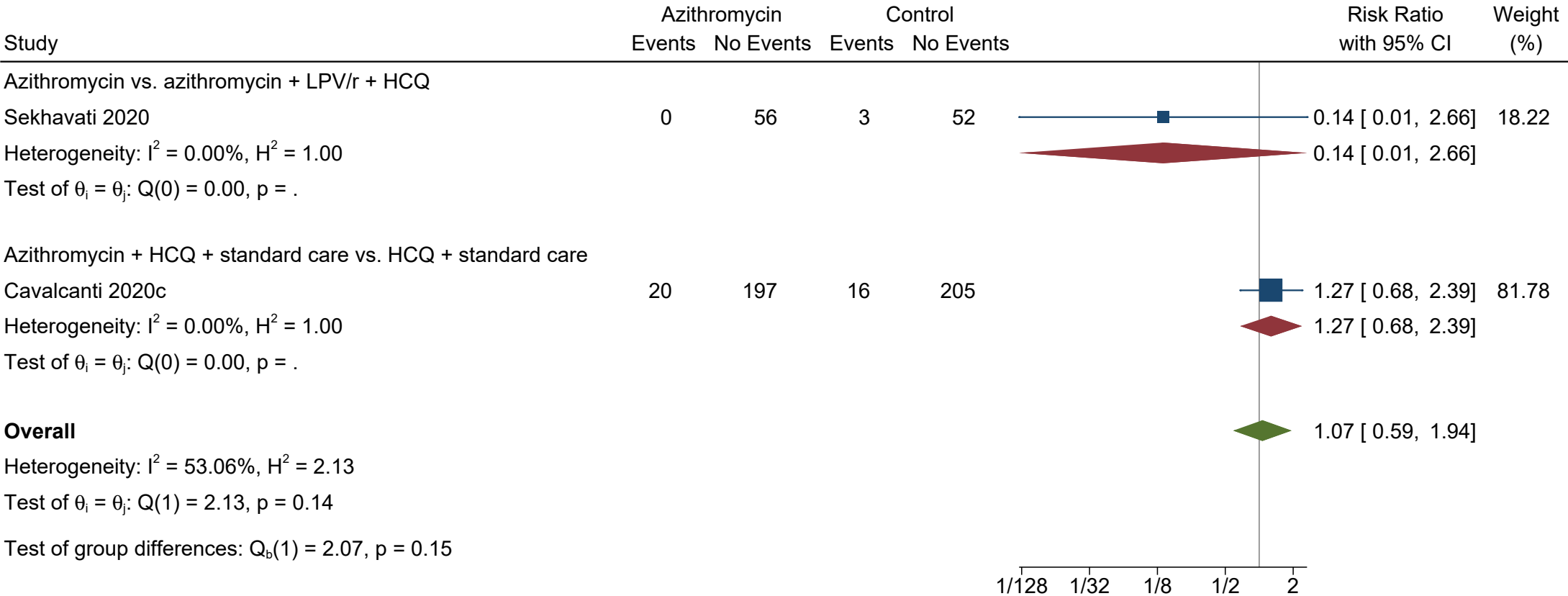

Fixed-effects Mantel-Haenszel model

Supplement: S44 Fig — (PDF) [file pone.0248132.s112.pdf]

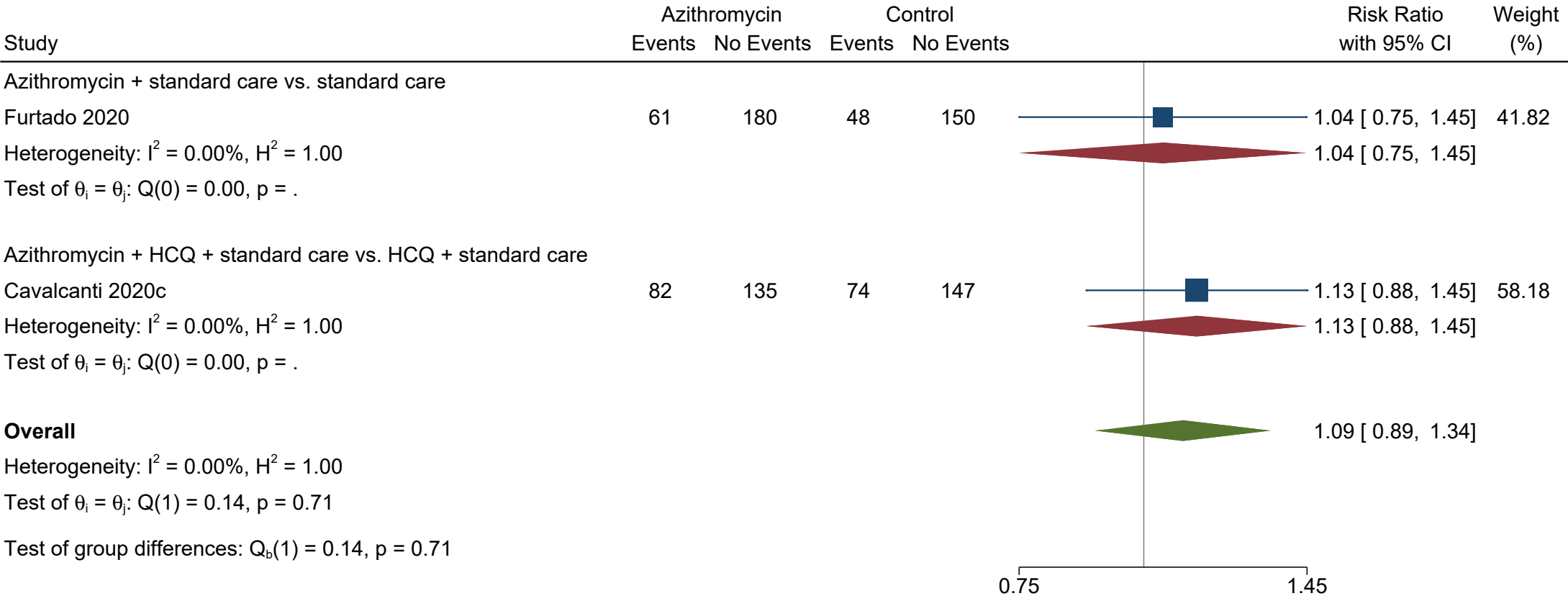

Fixed-effects Mantel-Haenszel model

Supplement: S45 Fig — (PDF) [file pone.0248132.s113.pdf]

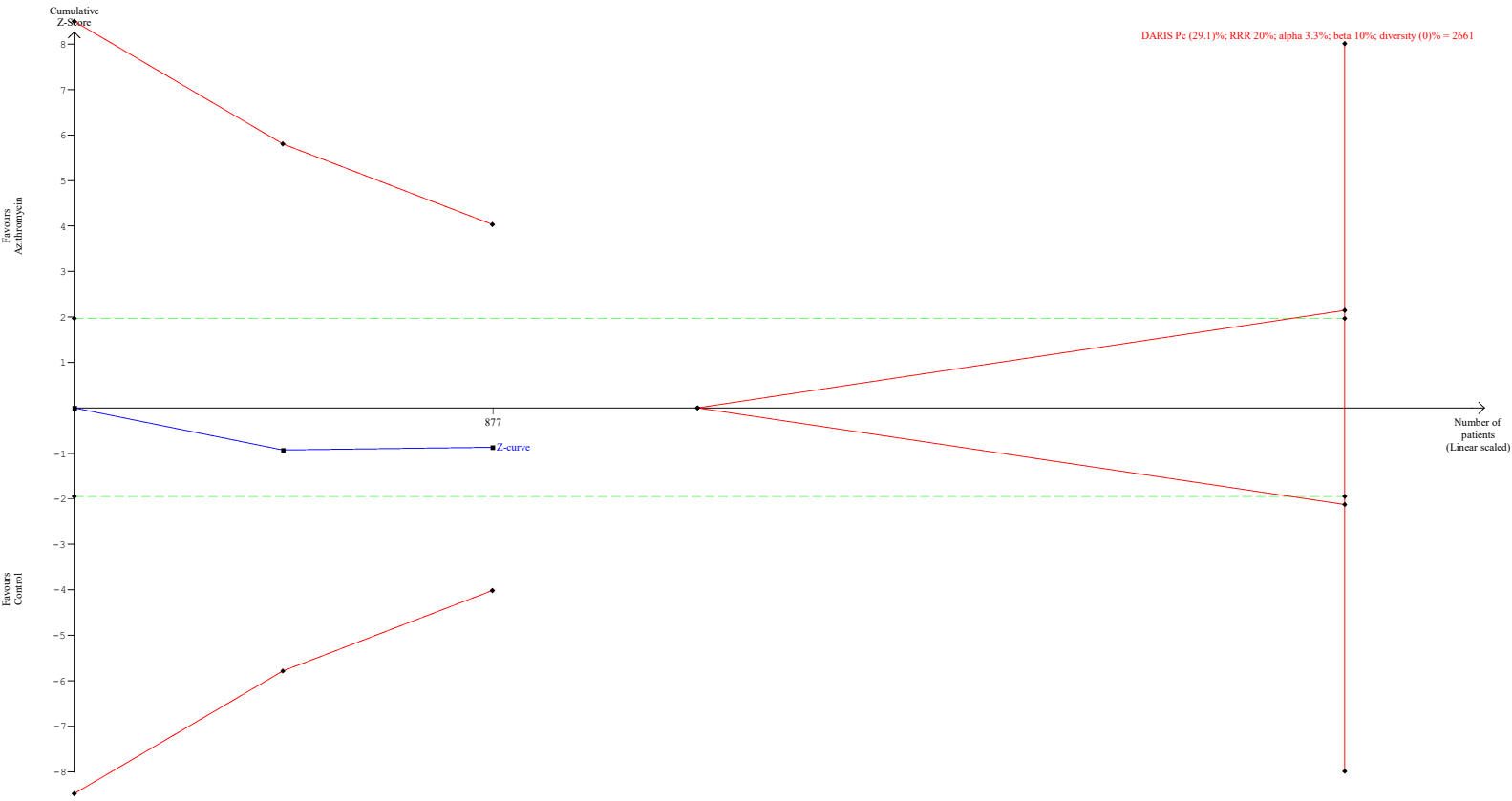

Supplement: S46 Fig — (PDF) [file pone.0248132.s114.pdf]

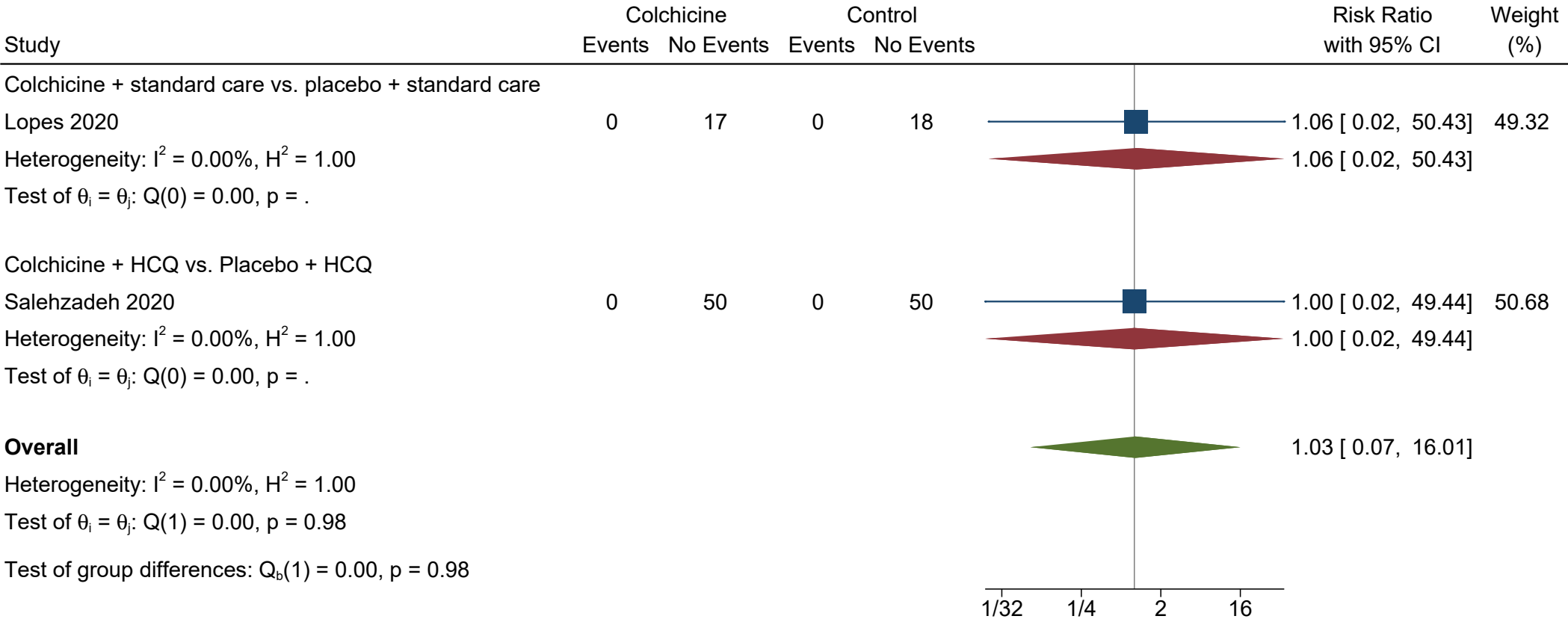

Supplement: S47 Fig — (PDF) [file pone.0248132.s115.pdf]

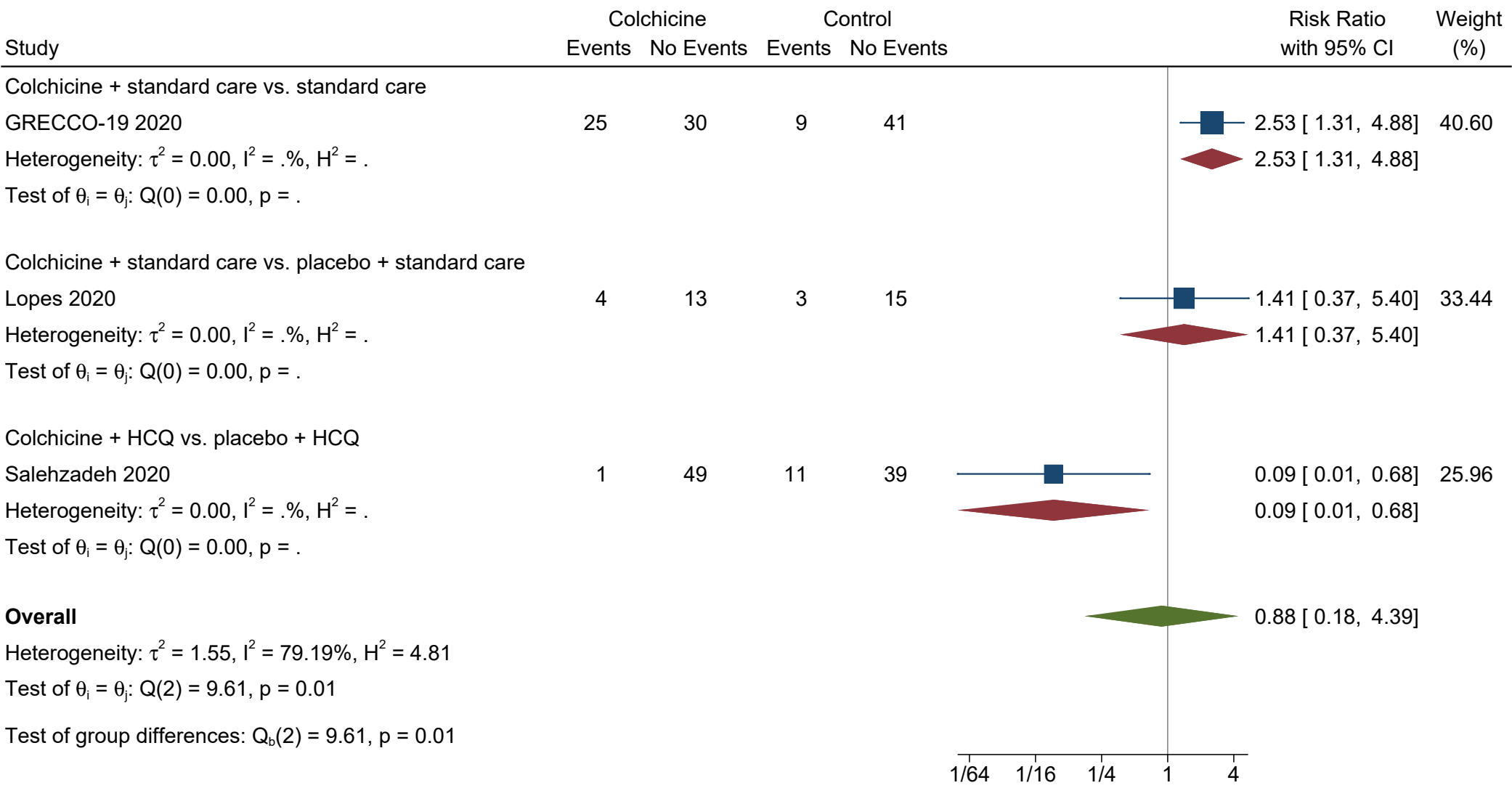

Supplement: S48 Fig — (PDF) [file pone.0248132.s116.pdf]

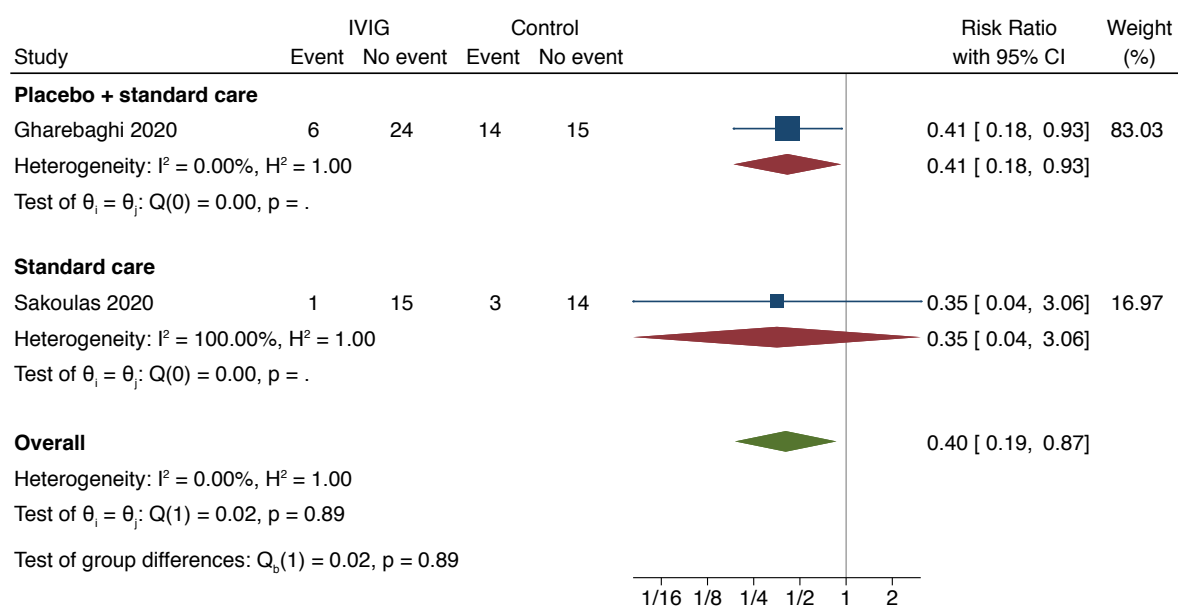

Fixed-effects Mantel-Haenszel model

Supplement: S49 Fig — (PDF) [file pone.0248132.s117.pdf]

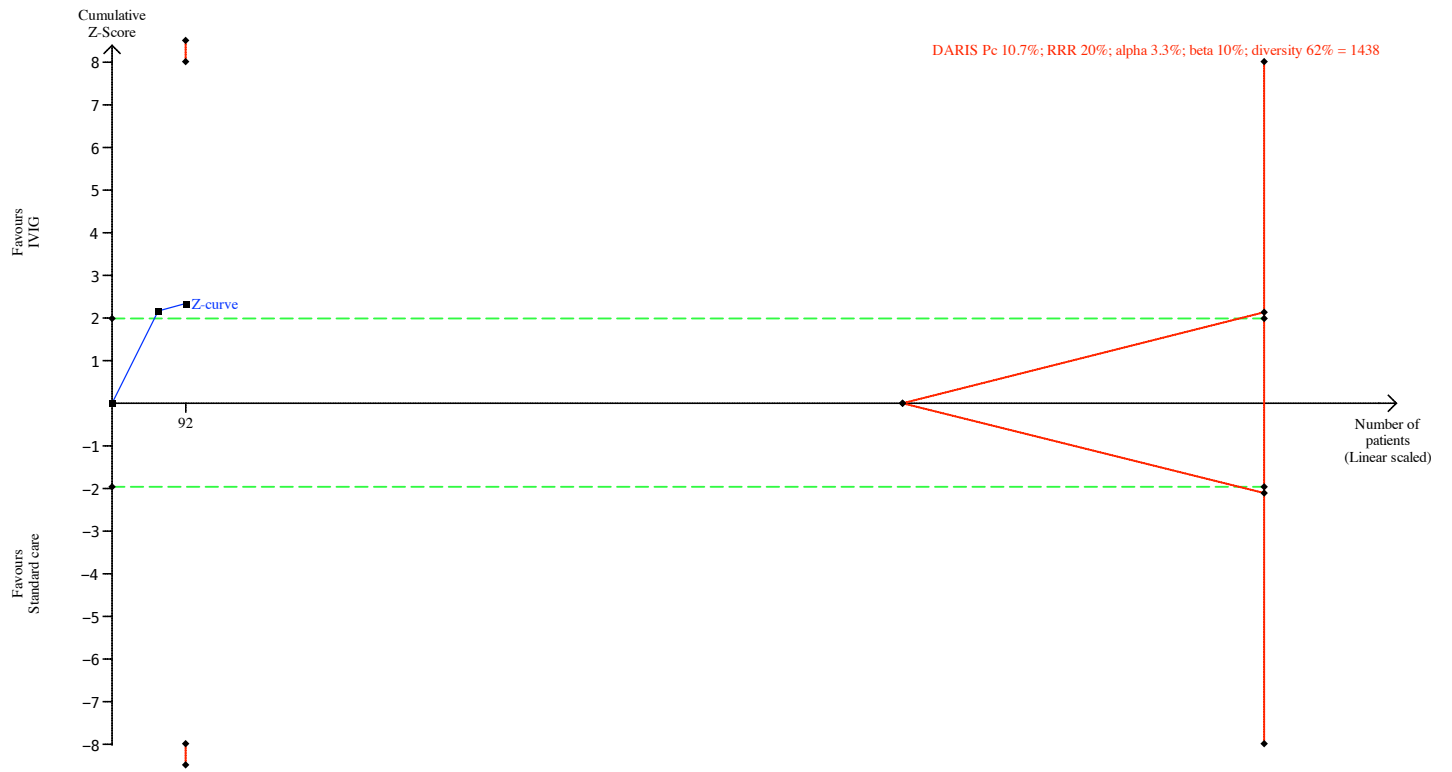

Supplement: S50 Fig — (PDF) [file pone.0248132.s118.pdf]

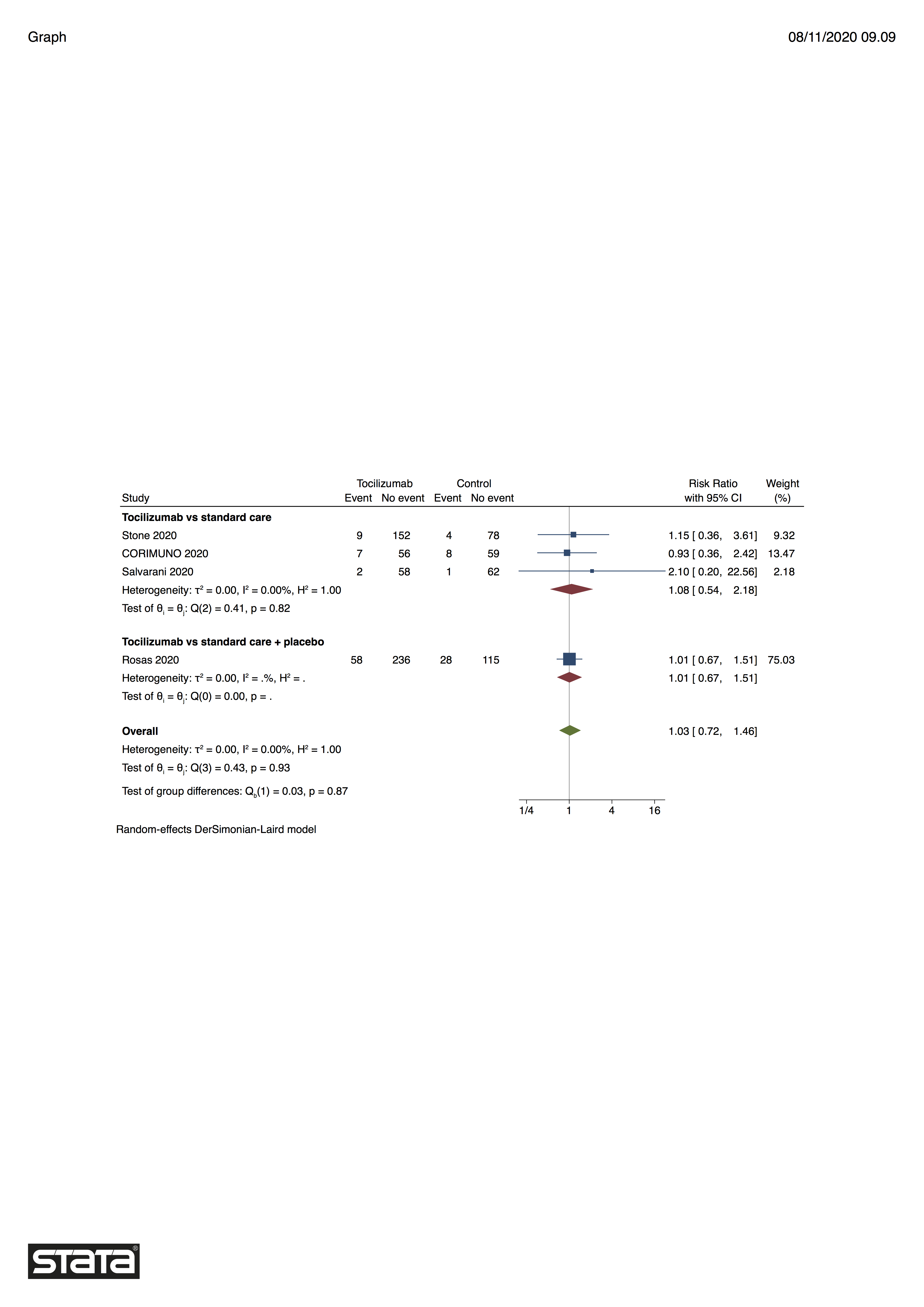

Supplement: S53 Fig — (TIFF) [file pone.0248132.s121.tiff]

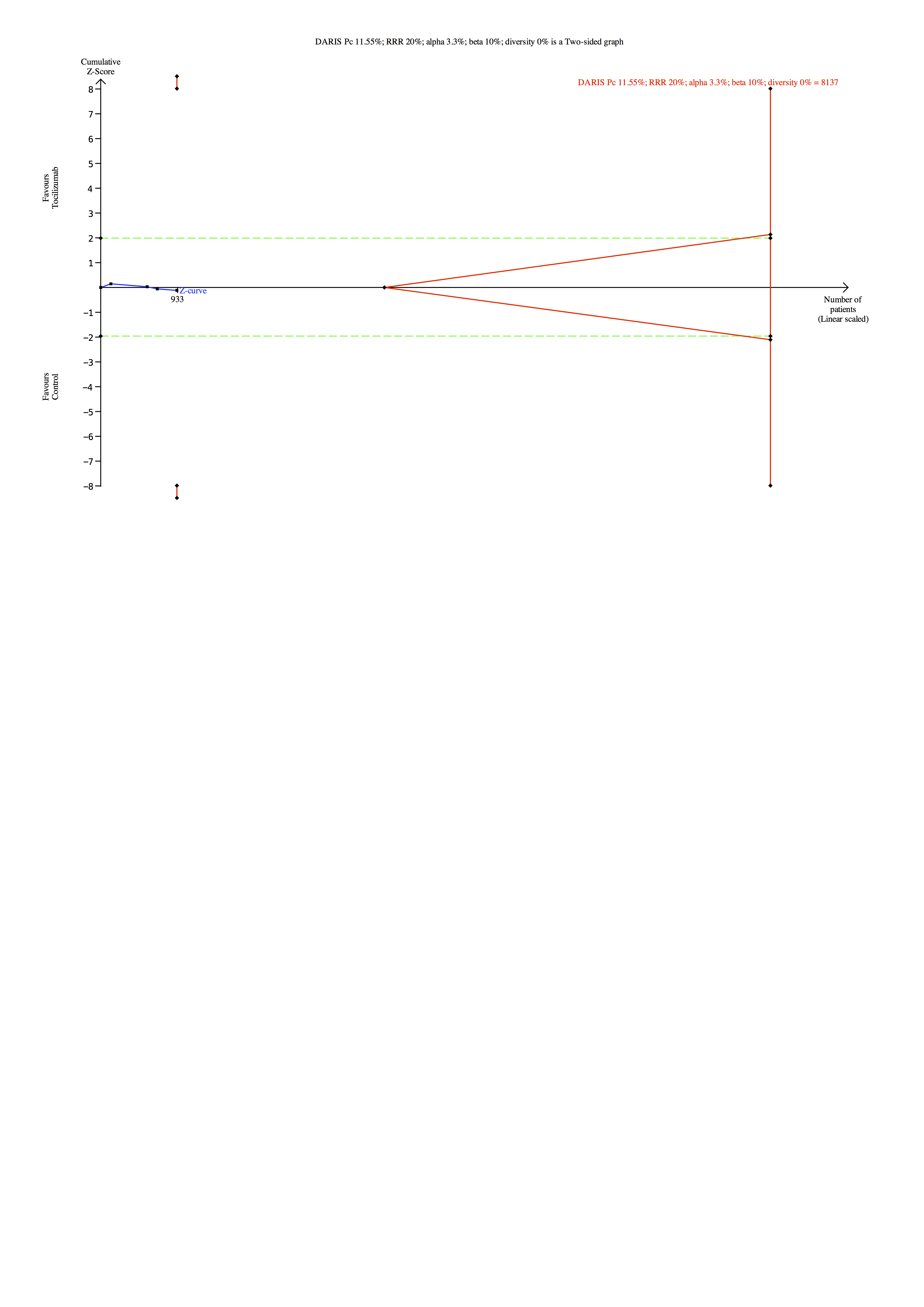

Supplement: S54 Fig — (TIFF) [file pone.0248132.s122.tiff]

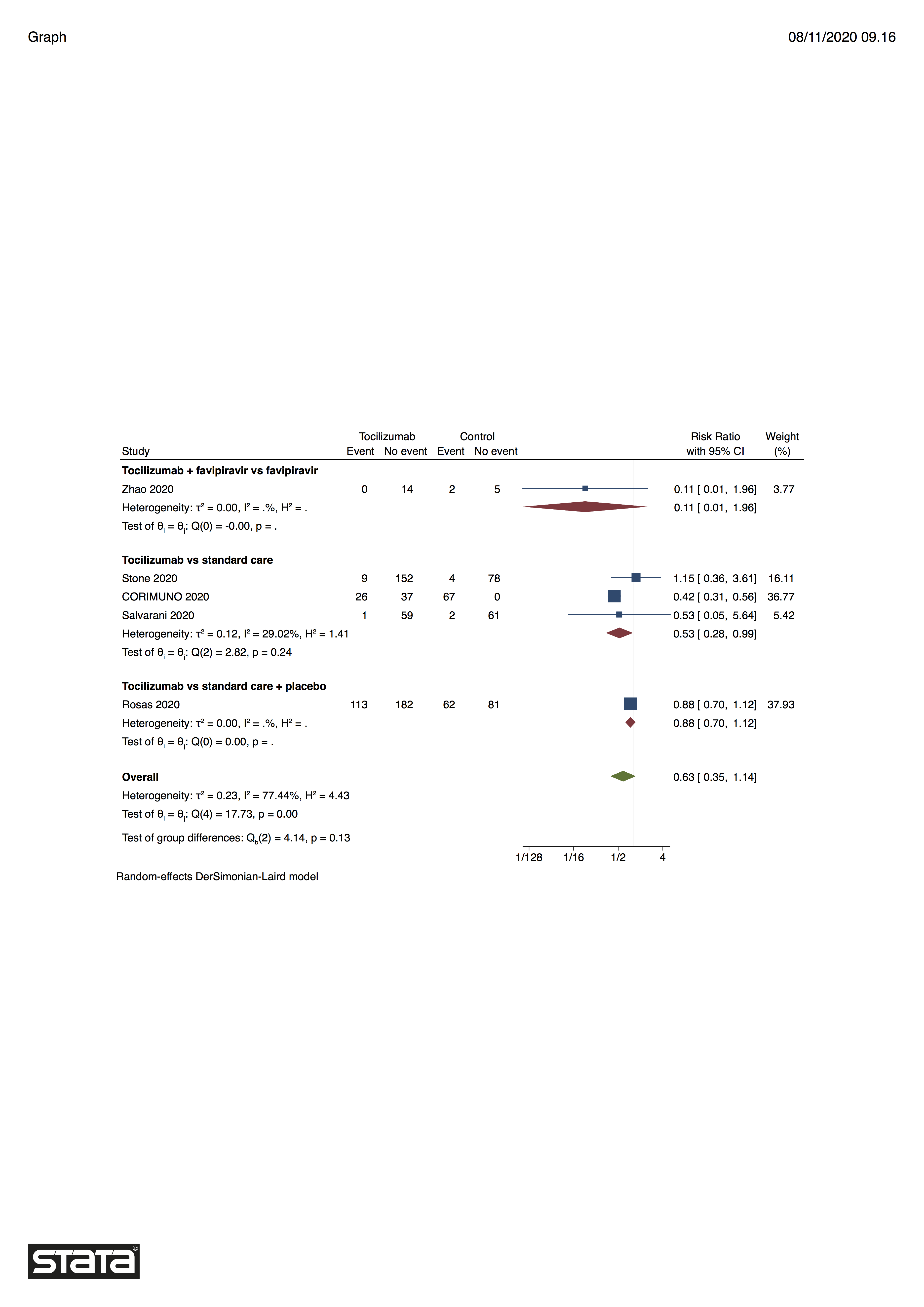

Supplement: S55 Fig — (TIFF) [file pone.0248132.s123.tiff]

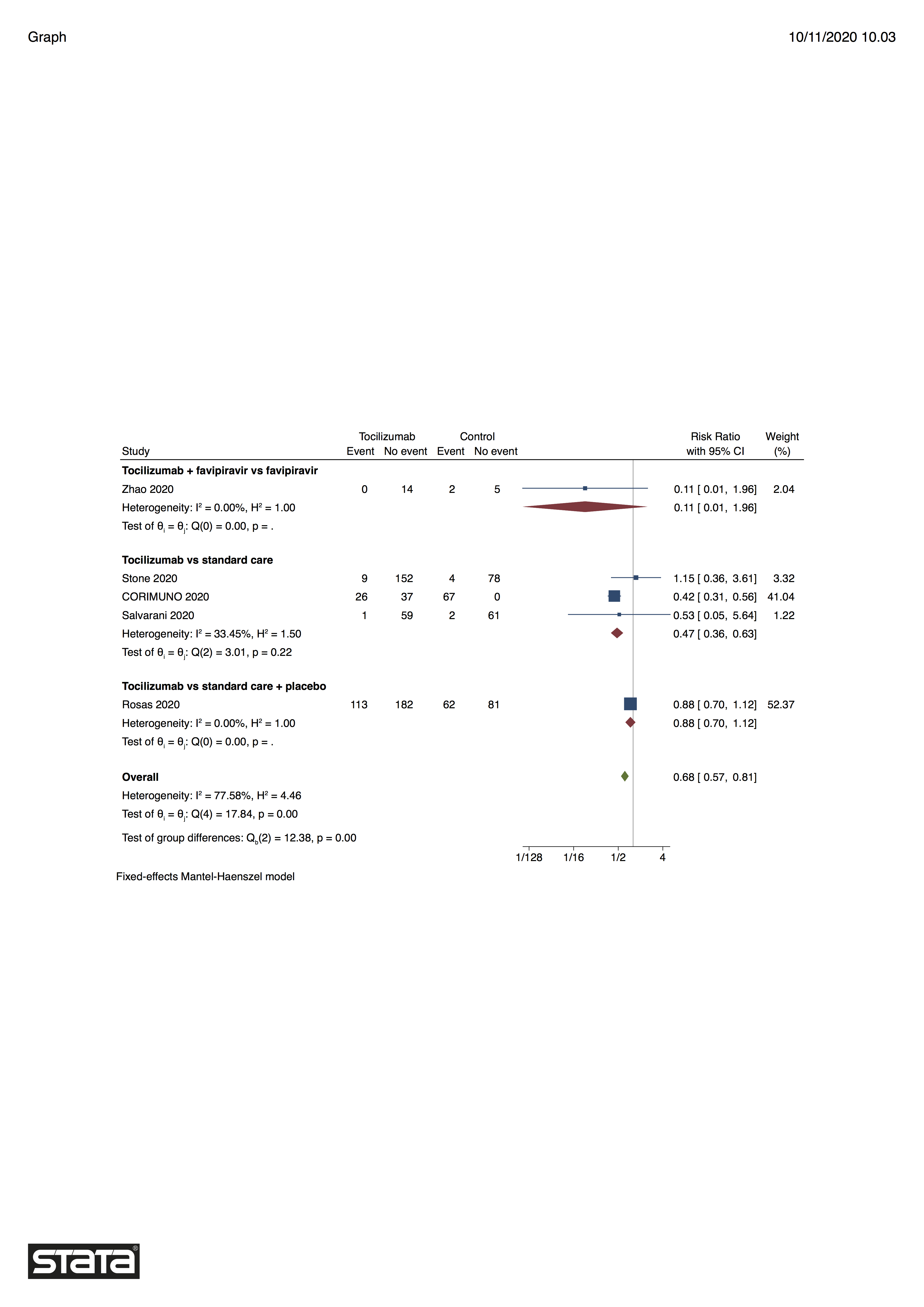

Supplement: S56 Fig — (TIFF) [file pone.0248132.s124.tiff]

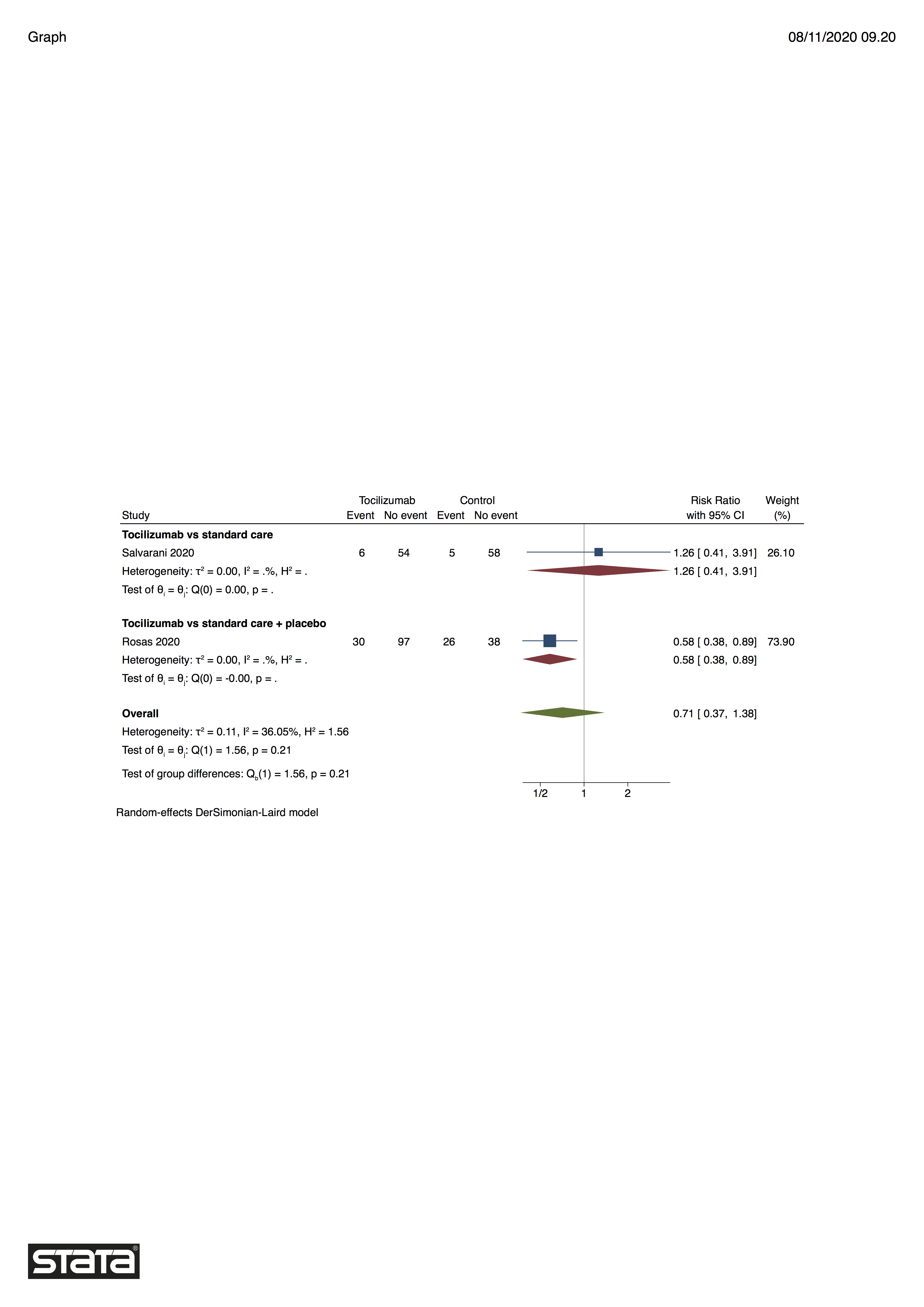

Supplement: S57 Fig — (TIFF) [file pone.0248132.s125.tiff]

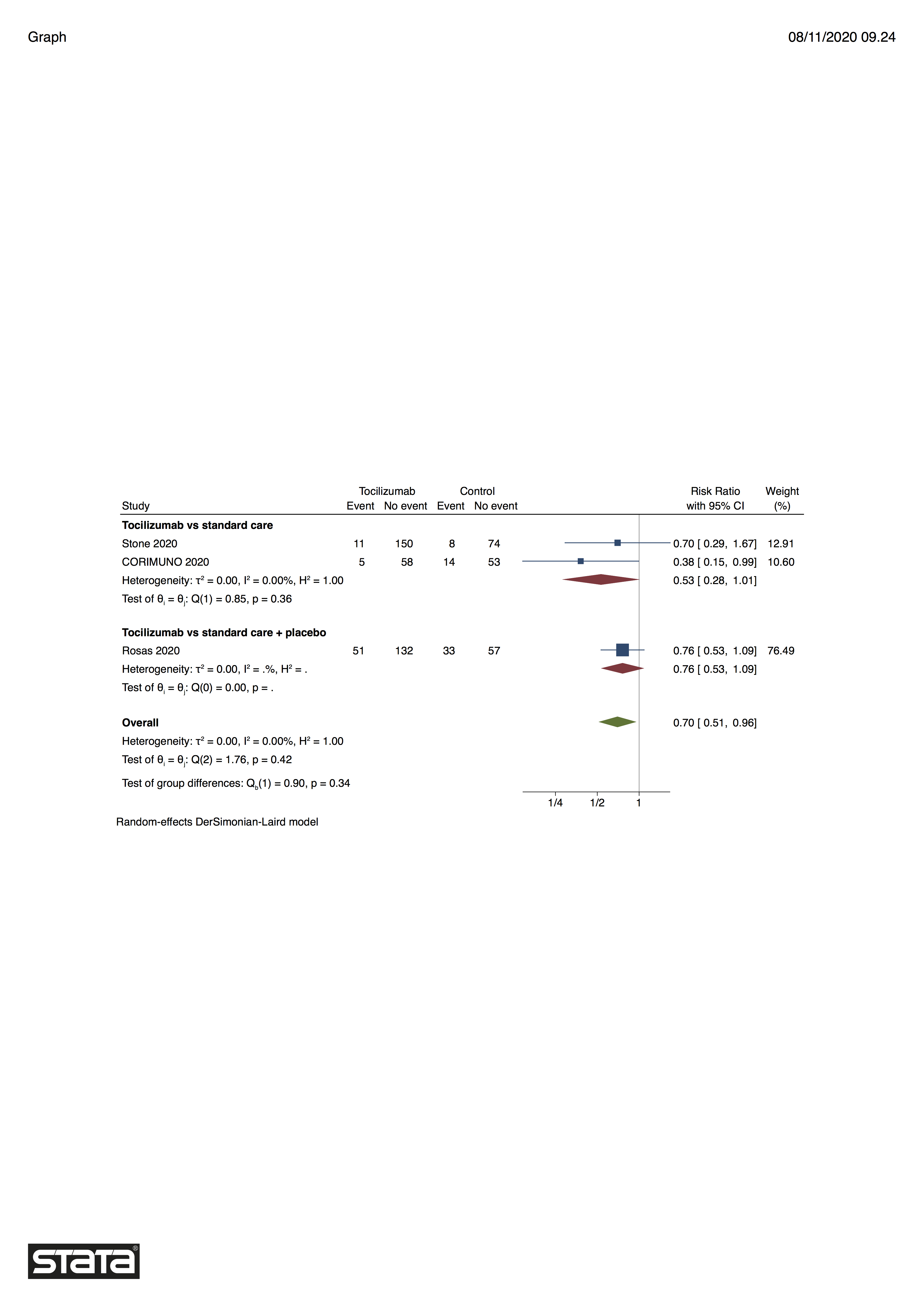

Supplement: S58 Fig — (TIFF) [file pone.0248132.s126.tiff]

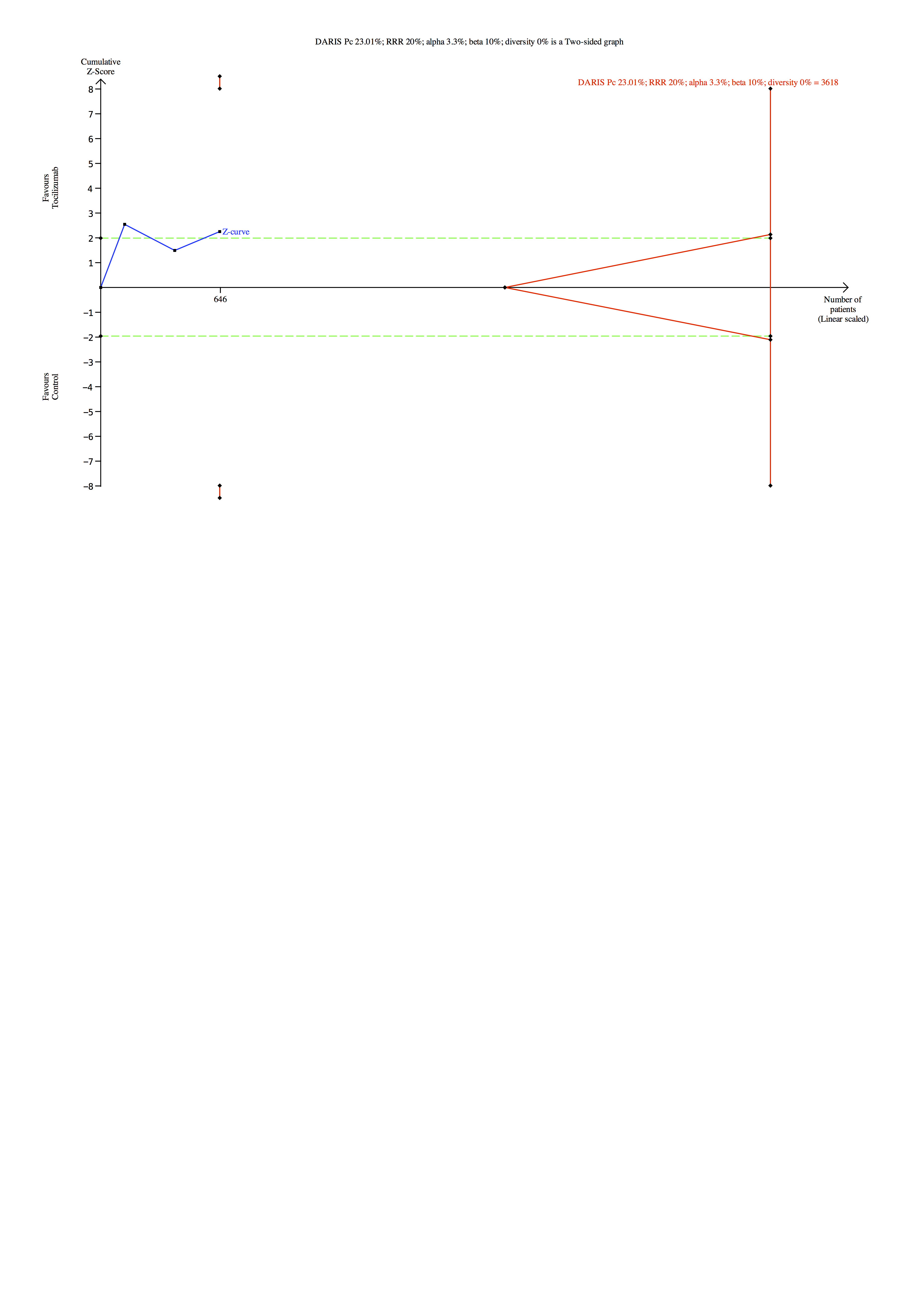

Supplement: S59 Fig — (TIFF) [file pone.0248132.s127.tiff]

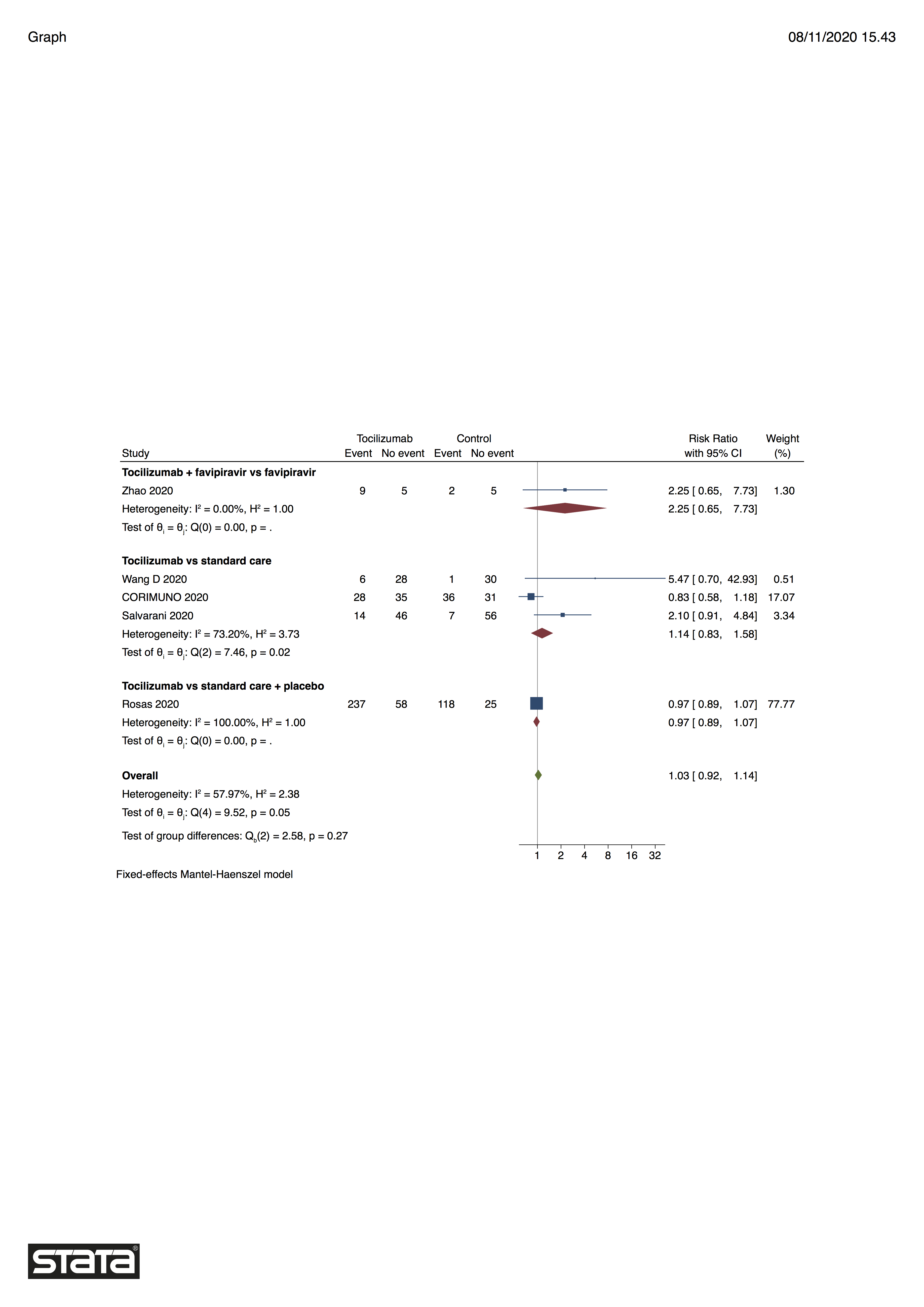

Supplement: S60 Fig — (TIFF) [file pone.0248132.s128.tiff]

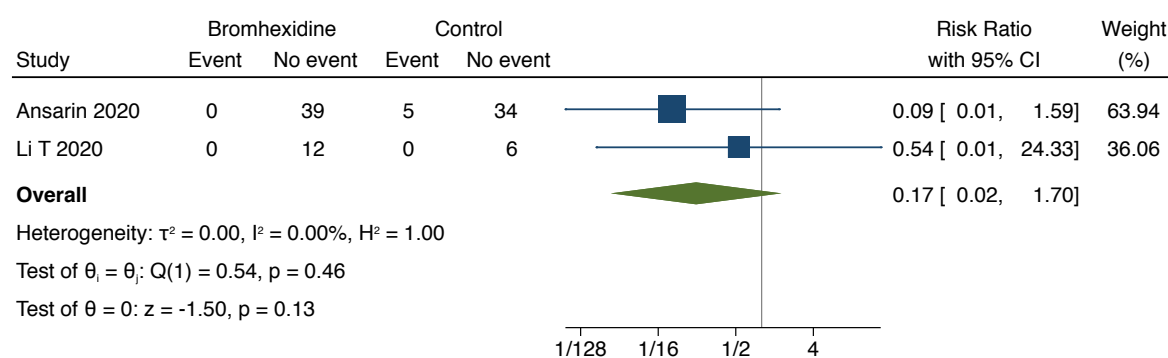

Random-effects DerSimonian-Laird model

Supplement: S61 Fig — (PDF) [file pone.0248132.s129.pdf]

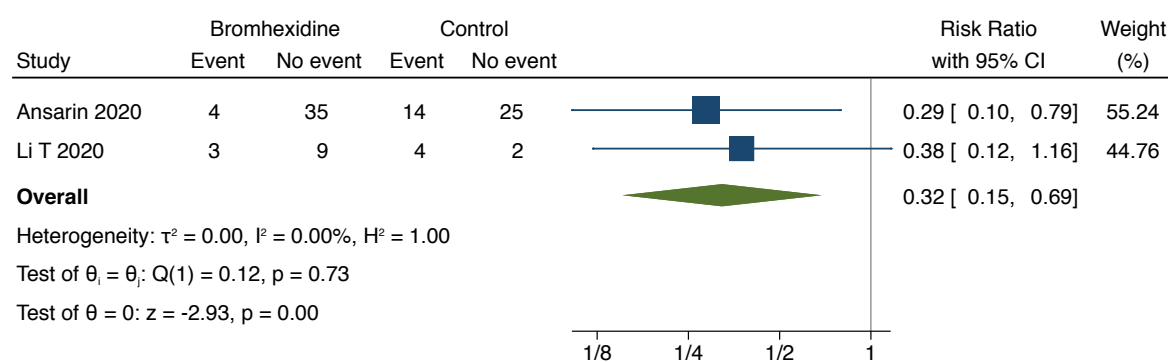

Random-effects DerSimonian-Laird model

Supplement: S62 Fig — (PDF) [file pone.0248132.s130.pdf]
